# Variable Ca‐C_aryl_ Hapticity and its Consequences in Arylcalcium Dimers

**DOI:** 10.1002/advs.202304765

**Published:** 2023-09-15

**Authors:** Kyle G. Pearce, Chiara Dinoi, Ryan J. Schwamm, Laurent Maron, Mary F. Mahon, Michael S. Hill

**Affiliations:** ^1^ Department of Chemistry University of Bath Claverton Down Bath BA2 7AY UK; ^2^ Université de Toulouse et CNRS INSA UPS UMR 5215 LPCNO 135 Avenue de Rangueil Toulouse F‐31077 France

**Keywords:** biaryls, calcium, density functional theory, main group chemistry

## Abstract

The dimeric β‐diketiminato calcium hydride, [(^Dipp^BDI)CaH]_2_ (^Dipp^BDI = HC{(Me)CN‐2,6‐*i*‐Pr_2_C_6_H_3_}_2_), reacts with *ortho*‐, *meta*‐ or *para*‐tolyl mercuric compounds to afford hydridoarylcalcium compounds, [(^Dipp^BDI)_2_Ca_2_(μ‐H)(μ‐*o*‐,*m*‐,*p*‐tolyl)], in which dimer propagation occurs either via μ_2_‐η^1^‐η^1^ or μ_2_‐η^1^‐η^6^ bridging between the calcium centers. In each case, the orientation and hapticity of the aryl units is dependent upon the position of the methyl substituent. While wholly organometallic *meta*‐ and *para*‐tolyl dimers, [(^Dipp^BDI)Ca(*m*‐tolyl)]_2_ and [(^Dipp^BDI)Ca(*p*‐tolyl)]_2_, can be prepared and are stable, the *ortho*‐tolyl isomer is prone to isomerization to a calcium benzyl analog. Computational analysis of this latter process with density functional theory (DFT) highlights an unusual mechanism invoking the generation of an intermediate dicalcium species in which the group 2 centers are bridged by a toluene dianion formed by the formal attachment of the original hydride anion to the initially generated *ortho*‐tolyl substituent. Use of a more sterically encumbered aryl substituent, {3,5‐*t*‐Bu_2_C_6_H_3_}, facilitates the selective formation of [(^Dipp^BDI)Ca(μ‐H)(μ‐3,5‐*t*‐Bu_2_C_6_H_3_)Ca(^Dipp^BDI)], which can be converted into the unsymmetrically‐substituted σ‐aryl calcium complexes, [(^Dipp^BDI)Ca(μ‐Ph)(μ‐3,5‐*t*‐Bu_2_C_6_H_3_)Ca(^Dipp^BDI)] and [(^Dipp^BDI)Ca(μ‐*p*‐tolyl)(μ‐3,5‐*t*‐Bu_2_C_6_H_3_)Ca(^Dipp^BDI)] by reaction with the appropriate mercuric diaryl. Conversion of [(^Dipp^BDI)Ca(H)(Ph)Ca(^Dipp^BDI)] to afford [{{(^Dipp^BDI)Ca}_2_(μ_2_‐Cl)}_2_(C_6_H_5_‐C_6_H_5_)], comprising a biphenyl dianion, is also reported. Although this latter transformation is serendipitous, AIM analysis highlights that, in a related manner to the *ortho*‐tolyl to benzyl isomerization, the requisite C–C coupling may be facilitated in an “across dimer” fashion by the experimentally‐observed polyhapto engagement of the aryl substituents with each calcium.

## Graphical abstract

A combined synthetic and computational study demonstrates that symmetrical and unsymmetrical β‐diketiminato calcium aryl dimers are accessible through reaction of a molecular calcium hydride and the appropriate mercuric aryl reagent. The resultant compounds display ligand‐dependent variations in the binding mode of the organometallic substituent, either μ_2_‐η^1^‐η^1^ or μ_2_‐η^1^‐η^6^, with higher hapticity interactions implicated in unusual “cross dimer” isomerization processes.

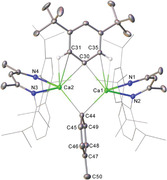

## 1 Introduction

Although it is now more than 120 years since the discovery of Grignard's eponymous reagents,^[^ ^1^ ^]^ organomagnesium compounds continue to prevail as some of the most generally useful and widely employed organometallics in chemical synthesis.^[^ ^2^ ^]^ In contrast, and despite sporadic reports of analogous RCaX species dating from a similar time period,^[^ ^3^ ^]^ organic derivatives of calcium remained largely neglected throughout much of the 20^th^ century and it is only during the past 20 years that a defined and characteristic chemistry has started to emerge.^[^ ^4^ ^]^ The groups of Anwander and Westerhausen have provided particularly notable recent contributions through their respective development and structural elucidation of dimethylcalcium and a wide variety of di‐ and monoarylcalcium reagents.^[^ ^5^ ^]^ Reports of subsequent onward reactivity for such species, however, remain relatively sparse,^[^ ^5^, ^6^ ^]^ most likely due to their limited tractability in comparison to magnesium‐derived systems.

**Scheme 1:**
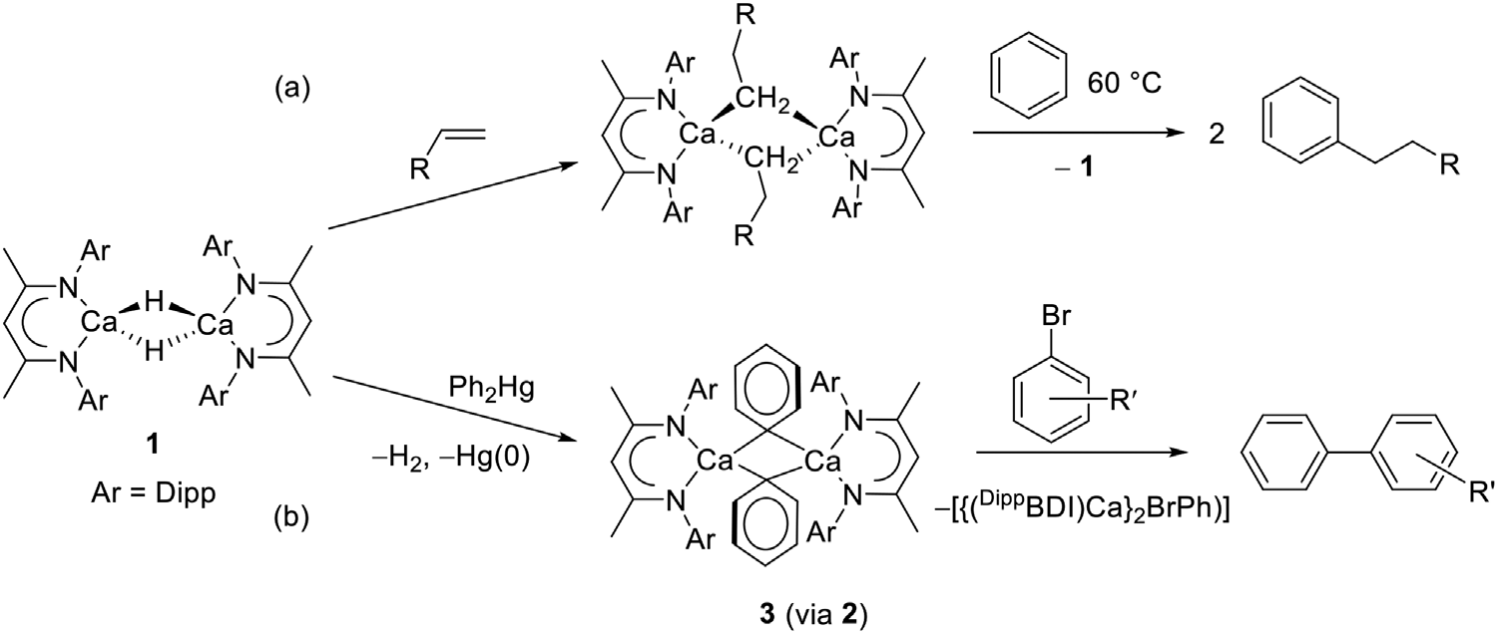
Use of compound **1** in the a) preparation of alkyl calcium complexes and the subsequent nucleophilic alkylation of benzene. b) synthesis of the calcium phenyl (**3**) and its reaction with aryl bromides to prepare biaryl molecules.

In parallel with these advances, the last 20 years have seen the more general emergence of calcium‐based reagents, primarily as Earth‐abundant and inexpensive vectors for homogeneous catalysis.^[^ ^7^ ^]^ Central to these latter efforts has been the use of a variety of multidentate uninegative supporting anions (L) as spectator ligands in the study of heteroleptic derivatives, LCaX (where X =, e.g., amide, phosphide, alkyl, hydride). While the primary requirements of L are to enhance LCaX solubility in non‐coordinating solvents and suppress otherwise deleterious Schlenk‐type equilibration, its steric demands must be appropriately and conveniently perturbed to maintain an appropriate level of kinetic reactivity at the Ca–X bonded unit. The β‐diketiminate (BDI) class of ligand has, thus, been particularly prominent in these advances.^[^ ^8^ ^]^ While recent developments have progressed to more sterically encumbered variants,^[^ ^9^ ^]^ the ^Dipp^BDI ligand (^Dipp^BDI = HC{(Me)CNDipp}_2_, where Dipp = 2,6‐*i*‐Pr_2_C_6_H_3_) has proved a reliable and readily accessible workhorse that has facilitated a wide variety of new chemistry. Reaction of the otherwise base‐free hydride complex, [(^Dipp^BDI)CaH]_2_ (**1**), with terminal alkenes has, for example, allowed access to a broad palette of dimeric σ‐alkyls, [(^Dipp^BDI)CaR]_2_.^[^ ^10^ ^]^ These compounds are sufficiently potent sources of hydride and alkyl anions, respectively, to effect the direct nucleophilic exchange of benzene C–H bonds (**Scheme** **1**a).^[^ ^10^, ^11^ ^]^

More recently, we have reported that the μ‐phenyl derivatives, [(^Dipp^BDI)Ca(μ‐H)(μ‐Ph)Ca(^Dipp^BDI)] (**2**) and [(^Dipp^BDI)Ca(μ‐Ph)]_2_ (**3**) are accessible via sequential reactions of compound **1** with Ph_2_Hg (Scheme 1b).^[^ ^12^ ^]^ Although the use of the mercuric reagent is a non‐ideal, though effective, synthetic expedient, these β‐diketiminato‐calcium phenyl derivatives allowed uncatalyzed access to biaryl molecules by direct S_N_Ar displacement of halide from arylbromides.^[^ ^6b^ ^]^ In this contribution, we report our investigation of the synthesis of alternative hydridoarylcalcium complexes, documenting a structural dependence between η^1^‐ and η^6^‐aryl coordination, synthetic access to unsymmetrical calcium aryl complexes of the form, [(^Dipp^BDI)Ca(μ‐Ar)(μ‐Ar')Ca(^Dipp^BDI)], as well as the serendipitous isolation of a biaryl‐coordinated calcium species and an assessment of the potential significance of variable aryl ligand hapticity for the future development of this chemistry.

## 2 Results and Discussion

**Scheme 2:**
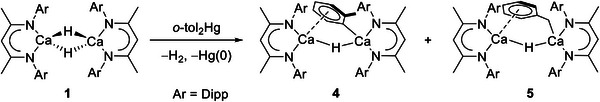
Synthesis of **4** and **5** from [(^Dipp^BDI)CaH]_2_ and (*o*‐tolyl)_2_ Hg.

Although the dimeric solid‐state structures of compounds **2** and **3** were both propagated by Ca‐μ_2_‐C_Ph_‐Ca interactions, the bridging phenyl substituents of the two compounds adopted contrasting orientations with respect to the calcium centers.^[^ ^12^ ^]^ Whereas the solitary phenyl group of **2** was effectively orthogonal to the Ca‐H‐Ca‐C_Ph_ least squares plane (88.4°), the corresponding angle subtended with the plane defined by the Ca‐C_Ph_‐Ca‐C_Ph_ heterocycle in compound **3** was only 24.88°. This latter unusual feature was attributed by density functional theory (DFT) calculations to a stabilizing interaction between the *ortho‐*CH bonds of the phenyl substituents and the coordinatively unsaturated calcium atoms. As an initial investigation of the potential generality of this *ortho‐*CH interaction, therefore, compound **1** was reacted with half an equivalent of (*o‐*tolyl)_2_ Hg at room temperature in benzene (**Scheme** **2**). In a manner reminiscent of that observed during the syntheses of compounds **2** and **3**, this reaction resulted in an immediate effervescence of H_2_ gas alongside the deposition of mercury metal. Two new sets of aryl environments could be identified in a 7:3 ratio in the resultant ^1^H NMR spectrum, which exhibited aromatic resonances at δ_H_ 6.79, 6.72, 6.60, 6.27 and 5.82, 5.65, 5.55 ppm, respectively (Figure S7, Supporting Information). The significantly lower frequency shifts and the apparent higher symmetry of the latter species, along with a corresponding 2H methylene signal observed at 1.90 ppm, allowed its tentative assignment as a benzylic calcium environment, which had apparently formed in competition with the initially envisaged *o‐*tolyl derivative.^[^ ^13^ ^]^ This supposition was subsequently confirmed by fractional crystallization of the reaction mixture and X‐ray diffraction analysis of both the *o‐*tolyl mixed hydride, [(^Dipp^BDI)Ca(H)(*o‐*tolyl)Ca(^Dipp^BDI)] (**4**), and its benzylic isomer (**5**).

**Figure 1:**
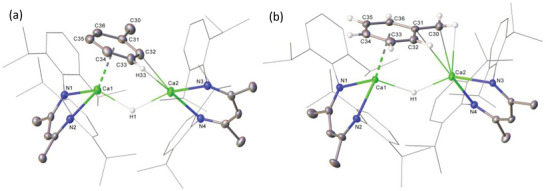
Displacement ellipsoid (30% probability) plots of the structures of a) compound **4** and b) compound **5**. For purposes of clarity, *N*‐Dipp substituents are presented as wireframes and hydrogen atoms (excepting H1 and those of the *o*‐tolyl (**4**) and benzyl (**5**) substituents) have been omitted along with the solvent and the minor disordered components in **5**. Selected bond lengths (Å) and angles (°): (**4**) Ca1‐N1 2.341(2), Ca1‐N2 2.355(2), Ca1‐C31 2.971(3), Ca1‐C32 2.870(3), Ca1‐C33 2.778(3), Ca1‐C34 2.859(3), Ca1‐C35 2.904(3), Ca1‐C36 2.947(3), Ca2‐N3 2.353(2), Ca2‐N4 2.353(2), Ca2‐H1 2.1927(6), Ca2‐C32 2.526(3), N1‐Ca1‐N2 79.83(7), N4‐Ca2‐N3 80.18(7). (**5**) Ca1‐N1 2.3436(12), Ca1‐N2 2.3542(12), Ca1‐C31 3.2233(15), Ca1‐C32 3.0836(16), Ca1‐C33 2.8801(17), Ca1‐C34 2.7599(17), Ca1‐C35 2.8197(15), Ca1‐C36 3.0036(15), Ca2‐N3 2.3511(11), Ca2‐N4 2.3437(12), Ca2‐C30 2.6036(18), Ca2‐C31 3.1744(15), N1‐Ca1‐N2 78.62(4), N4‐Ca2‐N3 78.62(4), C31‐C30‐Ca2 100.38(11).

The introduction of a methyl group into the *ortho‐*C position of compound **4** evidently disrupts any potential for the symmetrical C–H∙∙∙Ca interactions that were a defining feature of compound **3** in the solid state. The resultant structure (**Figure** **1**a), thus, comprises two differentiated calcium environments, where Ca1 is coordinated by two N–Ca contacts [2.341(1) and 2.355(2) Å], a bridging hydride ligand [2.0825(5) Å] and an η^6^‐arene interaction to the *o‐*tolyl moiety with a centroid to calcium distance of 2.5276(11) Å. Ca2 is also coordinated to two β‐diketiminate nitrogen atoms [2.353(2) Å] and a bridging hydride interaction [2.1927(6) Å], but displays a close contact with C32 [2.526(3) Å], identifiable as the *ipso‐*carbon delivered by the original *o*‐tolyl mercuric reagent. Although the calcium coordination environments of compound **5** are broadly comparable to those of **4** (Figure 1b), the η^6^‐arene to Ca1 centroid distance of 2.6150(9) Å is significantly longer. This feature may be attributed to the Ca2–C30 σ‐bond [2.6036(18) Å] that provides the interaction between calcium and the now benzylic C30 carbon, but which is elongated in comparison to the Ca‐C(*sp^2^*) σ‐aryl interaction observed in **4**. These bond lengths are, however, broadly commensurate with previously reported arene‐Ca contacts and benzylic C–Ca interactions.^[^ ^9^, ^12^, ^13^, ^14^ ^]^

Although we believe complex **5** is formed via isomerization from compound **4**, we have been unable to study this transformation experimentally due to the longer‐term solution instability of both compounds. For this reason, a DFT study at the B3PW91‐D3 level of theory was performed on the possible mechanistic pathway involved in this isomerization process. The optimized structures of **4** (**4_opt_**) and **5** (**5_opt_**) are shown in Figure S34 (Supporting Information). Calculated bond distances are listed in **Table** **1** and are compared with those experimentally observed.

**Table 1:** Comparison of calculated (**4_opt_** and **5**_opt_) and experimentally‐observed (**4** and **5**) bond distances.

Bond Distances	4	4_opt_	5	5_opt_
Ca1‐H1	2.0825(5)	2.11068	2.1283(5)	2.05789
Ca1‐C31	2.971(3)	3.09190	3.2233(15)	3.41617
Ca1‐C32	2.870(3)	2.87883	3.0836(16)	3.32707
Ca1‐C33	2.778(3)	2.74906	2.8801(17)	3.04486
Ca1‐C34	2.859(3)	2.85206	2.7599(17)	2.78625
Ca1‐C35	2.904(3)	2.99666	2.8197(15)	2.86094
Ca1‐C36	2.947(3)	3.11517	3.0036(15)	3.13835
Ca2‐H1	2.1927(6)	2.13925	2.1015(4)	2.07855
Ca2‐C32	2.526(3)	2.50270	–	–
Ca2‐C30	–	–	2.6036(18)	2.59159

The computed distances globally reproduce the values obtained experimentally, validating the level of the computational method employed. Variations higher than 0.04 Å between the experimental and computed bond distances were found for some Ca1–C(η^6^‐arene) bond lengths, providing Ca1–(η^6^‐arene) centroid distances of 2.593 Å versus 2.5276(11) Å for **4_opt_** and 2.765 Å versus 2.6150(9) Å for **5_opt_**. These disparities are ascribed to the presence of intermolecular short contacts within the crystal lattice of both **4** and **5**. In order to evaluate the effect of the introduction of a methyl group into the *ortho*‐C position of the arene ligand on the charges of the Ca centers, we computed the natural charges of **4** and **5** via an NBO analysis. As shown in Figure S34 (Supporting Information), the presence of two differentiated calcium environments in compounds **4** and **5** does not affect the charges of the two Ca atoms, which are practically identical in both compounds (+1.75 for Ca1 and Ca2 in **4_opt_** and +1.76 for Ca1 and Ca2 in **5_opt_**).

**Figure 2:**
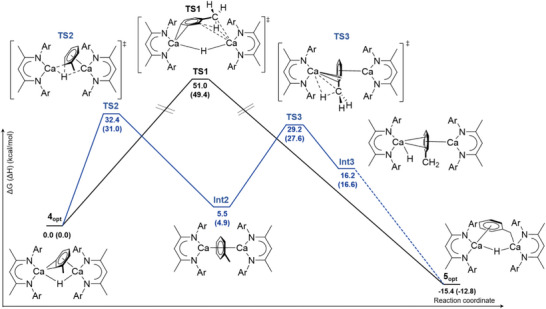
Gibbs free energy (enthalpy) profiles at the D3‐B3PW91 level of theory (6‐311++G** for Ca, 6–311G** N, O and 6–31G**) for the formation of complex **5** (**5**_**opt**_) starting from compound **4** (**4_opt_**). Black: Gibbs free energy (enthalpy) profile of the direct one‐step isomerization, involving the transfer of one H atom from the methyl group to the *ipso*‐carbon of the *o*‐tolyl ligand. Blue: Gibbs free energy (enthalpy) profile of the three‐step isomerization, involving i) the reaction of the bridging Ca_2_(μ‐H) hydride ligand with the *ipso*‐carbon of the bridging *o*‐tolyl group, followed by ii) the transfer of one hydride from the methyl group to one Ca center and by iii) the isomerization of the bridging C_7_H_7_^−^ anion toward the formation of the benzylic hydride product **5_opt_**.

In order to rationalize the formation of complex **5**, we focused on the mechanism involved in the isomerization reaction between **4** and **5**. As shown in the Gibbs free energy (enthalpy) profiles in **Figure** **2**, two mechanistic pathways have been computed, displaying either a direct one‐step H transfer within the *o*‐tolyl ligand (black profile) or a three‐step process involving the bridging Ca_2_(μ‐H) hydride ligand (blue profile). The geometries of all the optimized structures in Figure 2 are shown in Figure S35 (Supporting Information), together with the most significant bond lengths and NPA charges. The formation of **5** is an exergonic process by 15.4 kcal mol^−1^, indicating that the benzylic isomer is more stable than its *o*‐tolyl derivative. Starting from compound **4**, the direct one‐step transfer of one H atom from the methyl group to the *ipso*‐carbon of the *o*‐tolyl ligand requires a transition state (**TS1**, ∆*G* = 51.0 kcal mol^−1^) that is unfeasibly high given the applied experimental conditions. Although this possibility may, thus, be discounted, a second computed pathway induced by the nucleophilic attack of the bridging Ca_2_(μ‐H) hydride anion at the *ipso*‐carbon of the *o*‐tolyl anion provides **Int2** with a C_7_H_8_^2−^ dianion, which bridges two (^Dipp^BDI)Ca^+^ units (Figure 2, blue pathway). This reaction is endothermic by 5.5 kcal mol^−1^ but invokes a more accessible transition state (**TS2**) of 32.4 kcal mol^−1^. Notably, an effective reverse of this reaction leading to the formation of the mixed μ‐H, μ‐Ph dicalcium dimer (**2**), has been recently proposed by Harder and coworkers to rationalize biphenyl dianion formation from a putative benzene dianion intermediate (vide infra).^[^ ^9^, ^15^ ^]^ Our current deductions, therefore, indicate a wider generality of such processes and, in common with an analogous benzene dianion strontium derivative reported in the same study,^[^ ^15^ ^]^ the structure of **Int2** presents a slightly puckered C_7_H_8_^2−^ dianion in a flattened boat form (max. C – C – C – C torsion angle: 15.9°). The Ca – C distances are in the range of 2.533 to 2.776 Å and the C–C bond distances (two shorter: 1.383 Å and four longer: in the range of 1.451 to 1.502 Å) are typical for a C_7_H_8_^2−^ ring with more localized negative charges at the two *para*‐positioned C atoms (−0.718 and −0.743, see Figure S35, Supporting Information).

From **Int2**, the C_7_H_8_^2−^ methyl group may then transfer one hydride to one Ca center, leading to the formation of an asymmetrical dinuclear [(^Dipp^BDI)Ca(κ^1^‐H)(μ_2_‐η^6^‐C_6_H_5_CH_2_)Ca(^Dipp^BDI)] complex, **Int3**. In this intermediate, as shown in Figure 2 and Figure S35 (Supporting Information), one Ca center is coordinated by two N–Ca contacts [2.379 and 2.358 Å], a terminal hydride ligand [2.061 Å] and an η^2^‐arene interaction with the bridging C_7_H_7_^−^ anion (C_arene_ to calcium distances of 2.832 and 2.803 Å). In contrast, the other Ca center is coordinated by two N–Ca contacts [2.325 and 2.296 Å] and an η^6^‐arene interaction with the bridging C_7_H_7_^−^ anion (centroid to calcium distance of 2.355 Å). The formation of **Int3** is endergonic by 16.2 kcal mol^−1^ with respect to **4_opt_**, via an associated kinetic barrier of 29.2 kcal mol^−1^. In the third and final step, the η^6^‐arene coordination mode of the bridging C_7_H_7_^−^ anion may evolve by an isomerization process toward the benzylic coordination mode of product **5_opt_**, with the formation of the benzylic C–Ca interaction providing the driving force of the reaction.

**Scheme 3:**
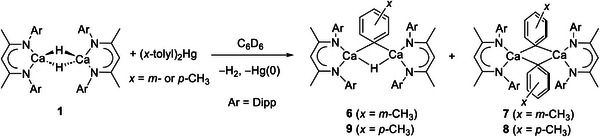
Synthesis of compounds **6** – **9**.

With compounds **4** and **5** in hand, and to assess the potential generality of the benzyl isomerization process, this synthetic protocol was extended to analogous *meta‐* and *para‐*phenyl methyl substitution. Accordingly, reactions of compound **1** with half an equivalent of (*x‐*tolyl)_2_ Hg (*x* = *m‐* or *p‐*) provided the familiar loss of H_2_ via rapid bubbling of the reaction solution and the formation of a mercury precipitate (**Scheme** **3**). Initial assessment of the *m‐*tolyl‐derived reaction by ^1^H NMR spectroscopy evidenced the generation of two calcium aryl species, which were identified by the appearance of two new *iso*‐propyl methine resonances at δ_H_ 3.16 and 2.96 ppm. Three singlet resonances were also observed across the ^Dipp^BDI γ‐methine and Ca‐μ‐H region (*ca*. 4–5 ppm), with the latter two signals integrating in a relative 1:2 ratio (Figures S12–S14, Supporting Information). Guided by the lack of discrimination apparent in our previous observations of the formation of compounds **2** and **3**, we ascribed the two products formed as a likely mixture of a calcium *meta‐*tolyl mixed hydride (**6**) and its dinuclear arylcalcium analog, [(^Dipp^BDI)Ca(*m‐*tolyl)]_2_ (**7**) (Scheme 3). In support of this assignment, treatment of the initial reaction mixture with a further 0.5 equivalents of (*m‐*tolyl)_2_ Hg resulted in the complete disappearance of the hydridic resonance and afforded exclusive access to compound **7**, which was characterized by *iso*‐propyl methine and ^Dipp^BDI γ‐methine resonances at δ_H_ 2.96 and 4.86 ppm, respectively.

**Figure 3:**
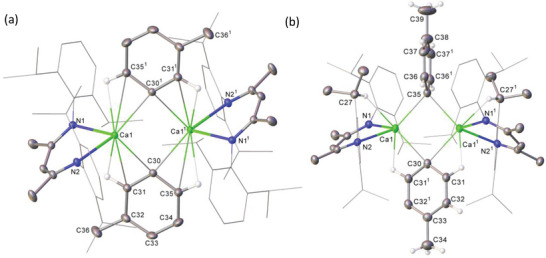
Displacement ellipsoid (30% probability) plots of the structures of a) compound **7** and b) compound **8**. For clarity, hydrogen atoms (excepting those attached to C31/C31^1^ and C35/C35^1^ (**7**) and C31/C31^1^ and C27/C27^1^ (**8**)), and disordered solvent (**7**) have been omitted. *N*‐Dipp substituents are presented as wireframes (*iso*‐propyl groups based on C27 excepted in **8**), also for visual ease. Selected bond lengths (Å) and angles (°): (**7**) Ca1‐N1 2.3630(11), Ca1‐N2 2.3651(12), Ca1‐C30 2.5444(15), Ca1‐C30 2.5491(14). N1‐Ca1‐N2 79.74(4), N1‐Ca1‐C30^1^ 118.26(4), N1‐Ca1‐C30 116.09(4). (**8**) Ca1‐N1 2.335(2), Ca1‐N2 2.371(2), Ca1‐C30 2.548(4), Ca1‐C35 2.571(4). N1‐Ca1‐N2 80.84(8), N1‐Ca1‐C30 120.75(7), N1‐Ca1‐C35 107.59(7), N2‐Ca1‐C30 109.63(9), N2‐Ca1‐C35 95.77(10). Symmetry operations: (**7**) ^1^ 1‐*x*, 1‐*y*, 1‐*z*. (**8**) ^1^ 1‐*y*, 1‐*x*, 3/2‐*z*.

This deduction was confirmed by X‐ray diffraction analysis, which identified compound **7** as a centrosymmetric σ‐aryl dimer in which the two *meta‐*methyl groups are oriented in opposing directions but with their corresponding aromatic units aligned in the same plane (**Figure** **3**a). As was previously observed in the structure of **3**,^[^ ^12^ ^]^ the aryl rings adopt an unusual orientation via engagement of each calcium with an *ortho* methine hydrogen atom of the aromatic substituents. As a result, the C_6_ planes subtend an acute angle of 22.47° with the least squares plane defined by the calcium atoms (Ca/Ca^1^) and the bridging *ipso*‐carbons (C30/C30^1^).

Two products were also observed in the reaction of **1** with half an equivalent of (*p‐*tolyl)_2_ Hg. In this instance, however, three *iso*‐propyl methine resonances were observed in the resultant ^1^H NMR spectrum alongside two differentiated *p‐*tolyl aromatic environments. Although comprehensive spectroscopic assignment was impeded by overlap of the various signals in this NMR spectrum (Figure S19, Supporting Information), these observations implied that a similar mixture of products had again been formed, albeit with an apparent reduction in symmetry across the calcium aryl dimer. After treatment of the reaction mixture with an additional half an equivalent of (*p‐*tolyl)_2_ Hg, the resultant ^1^H NMR spectrum presented only two of the initial *iso*‐propyl methine signals at 2.91 and 3.09 ppm. Further ^Dipp^BDI resonances (δ_H_ 4.86, 4.77 ppm), which integrated in a 1:1 ratio, in addition to a notable absence of any signal that could be assigned to a hydridic environment, supported a formulation of [(^Dipp^BDI)Ca(*p‐*tolyl)]_2_ (**8**).

The formation of colorless single crystals from a cooled (−35 **°**C) saturated toluene solution of **8**, allowed its structural confirmation by X‐ray diffraction as the unsymmetrical dimeric structure inferred by NMR spectroscopy (Figure 3b). In contrast to the symmetrical structures of both **3** and **7**, the C30‐ and C35‐containing *p‐*tolyl rings are differentiated by their respective orientations. While the C35‐containing carbocycle is almost orthogonal to the Ca1‐C30‐Ca1^1^‐C35 least squares plane (82.36°), the C30‐containing ring is constrained to lie in a similar plane (30.39°) through the adoption of *ortho*‐C–H∙∙∙Ca interactions familiar from the structures of **3** and **7**. In this case, however, coordinative unsaturation of the calcium centers is further satisfied by close contacts to the C27/C27^1^ methine C‐H bonds of two *N*‐Dipp *iso*‐propyl substituents.

**Figure 4:**
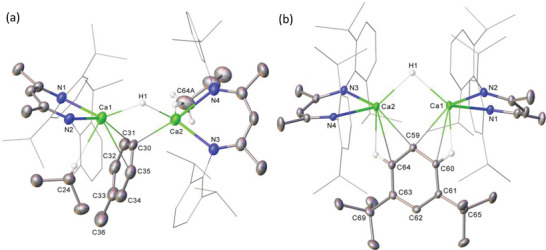
Displacement ellipsoid (30% probability) plots of the structures of a) compound **9** and b) compound **10**. For clarity, hydrogen atoms (excepting H1 and those attached to C24 and C64a (**9**) and C60 and C64 (**10**)), the second disordered component (**9**) and solvent (**9** and **10**) are omitted for clarity. Also, for visual ease, *N*‐Dipp substituents are presented as wireframes (*iso*‐propyl groups containing C12 and C64A excepted in **9**). Selected bond lengths (Å) and angles (°): (**9**) Ca1‐N1 2.329(3), Ca1‐N2 2.362(3), Ca1‐C30 2.565(3), Ca1‐H 2.2074(19), Ca2‐N3 2.338(3), Ca2‐N4 2.360(3), Ca2‐C30 2.586(3), Ca2‐H 2.2231(13), N1‐Ca1‐N2 79.19(9). (**10**) Ca1‐N1 2.3285(10), Ca1‐N2 2.3178(11), Ca1‐C59 2.5576(13), Ca1‐H 2.2811(15), Ca2‐N3 2.3194(10), Ca2‐N4 2.3266(10), Ca2‐C59 2.5387(12), Ca2‐H 2.2676(3), N2‐Ca1‐N1 82.46(4).

Crystals of the mixed aryl‐hydride dimer, [(^Dipp^BDI)Ca(μ‐H)(μ‐*p‐*tolyl)Ca(^Dipp^BDI)] (**9**), were also grown from a saturated benzene solution, permitting an X‐ray diffraction study (**Figure** **4**a). Much like the phenyl‐hydride species, [(^Dipp^BDI)Ca(μ‐H)(μ‐Ph)Ca(^Dipp^BDI)] (**2**), the calcium atoms are connected via a bridging hydride [≈2.215 Å], and a Ca–μ_2_–C–Ca interaction [≈2.576 Å], exhibiting comparable bond lengths and a similar orientation with respect to the aromatic unit.^[^ ^12^ ^]^ Unlike the previously reported phenyl analog, however, the aromatic ring itself is canted toward Ca1, reducing the Ca1‐centroid distance to 3.5859(2) Å relative to the analogous measurement to Ca2, [3.9064(3) Å].

**Scheme 4:**
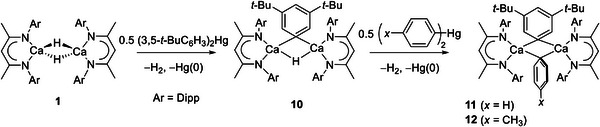
Synthesis of compounds **10** – **12**.

Although the structural characterization of compounds **2**, **4** and **9** demonstrates the viability of such dinuclear hydrido‐phenyl and hydrido‐tolyl calcium complexes, their synthesis via the relevant mercuric aryl reagent was invariably accompanied by the corresponding wholly aryl‐bridged compounds. Our study progressed, therefore, to the use of (3,5‐*t*‐Bu_2_C_6_H_3_)_2_Hg, expecting that the incorporation of additional steric bulk on the distal side of the aromatic unit would enable an enhanced level of kinetic discrimination. Accordingly, a reaction of compound **1** with half an equivalent of (3,5‐*t*‐Bu_2_C_6_H_3_)_2_Hg provoked the familiar evolution of H_2_ gas as well as the slow deposition of mercury metal. Although this process required a longer reaction time of 72 hours to reach completion, assessment at this point by ^1^H NMR spectroscopy revealed two characteristic resonances assigned to new ^Dipp^BDI γ‐methine and Ca‐μ‐H environments at δ_H_ 4.76 and 4.58 ppm. These signals emerged in a mutual 2:1 ratio by relative integration, alongside a broad *iso*‐propyl *N‐*Dipp methine signal at 3.11 ppm and two discriminated (9H) *tert*‐butyl resonances. (Figure S22, Supporting Information). These spectroscopic features and, particularly, the observation of an aryl *ipso‐*carbon signal at 177.7 ppm in the corresponding ^13^C{^1^H} NMR spectrum (Figures S23–S25, Supporting Information) were strongly indicative of the exclusive formation of [(^Dipp^BDI)Ca(H)(3,5‐*t*‐Bu_2_C_6_H_3_)Ca(^Dipp^BDI)] (**10**, **Scheme** **4**). This deduction was confirmed by a subsequent X‐ray diffraction analysis (Figure 4b), which revealed a further dimeric μ‐H‐μ‐aryl‐bridged structure. While the bond lengths and angles about the calcium centers are only marginally perturbed relative to those of the most directly comparable species described above, and the structure maintains the *ortho*‐C–H⋅⋅⋅Ca interactions that provide a distinctive feature in the structures of compounds **3**, **7** and **8**, the aromatic ligand itself adopts a somewhat more skewed orientation (36.3**°**) with respect to the plane defined by Ca1, C59 and Ca2.

**Figure 5:**
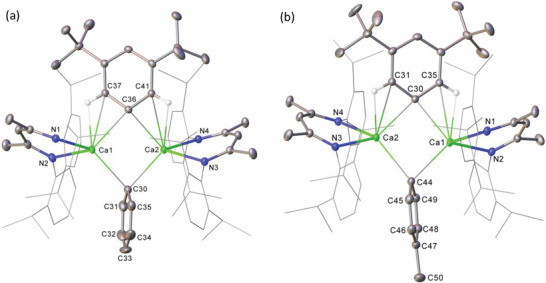
Displacement ellipsoid (30% probability) plots of the structures of a) compound **11** and b) compound **12**. For clarity, hydrogen atoms (excepting those attached to C37 and C41 (**11**) plus C31 and C25 (**12**)), solvent (**11**) and minor disordered components (**12**) are omitted for clarity. Also, for visual ease, *N*‐Dipp substituents are presented as wireframes. Selected bond distances (Å): (**11**) Ca1‐N1 2.3602(10), Ca1‐N2 2.3416(10), Ca1‐C30 2.5598(13), Ca1‐C36 2.5665(11), Ca2‐N3 2.3426(9), Ca2‐N4 2.3727(9), Ca2‐C30 2.5602(12), Ca2‐C36 2.5604(12). (**12**) Ca1‐N1 2.3755(9), Ca1‐N2 2.3403(9), Ca1‐C30 2.5486(11), Ca1‐C44 2.5659()12, Ca2‐N3 2.3422(9), Ca2‐N4 2.3748(9), Ca2‐C30 2.5356(11), Ca2‐C44 2.5659(12).

Attempts to react compound **10** with further equivalents of (3,5‐*t*‐Bu_2_C_6_H_3_)_2_Hg provided no evidence of reaction, presumably as a consequence of the increased steric demands of the dialkylated aryl units. With complex **10** in hand, however, we turned our attention to its potential to act as a synthon for wholly aryl bridged, but asymmetric [(^Dipp^BDI)Ca(μ‐Ar)(μ‐Ar')Ca(^Dipp^BDI)] systems. An initial reaction between **10** and 0.5 equiv. of Ph_2_Hg, induced the customary evolution of H_2_ and deposition of elemental mercury. Analysis of the resultant solution by ^1^H NMR spectroscopy revealed significant asymmetry within the resultant compound (**11**), albeit only a single ^Dipp^BDI γ‐methine environment at δ_H_ 4.77 ppm could be discriminated. Moreover, the aromatic region exhibited characteristic peaks for both the phenyl (δ_H_ 6.91, 6.65, 6.54 ppm) and 3,5‐*t*‐Bu_2_C_6_H_3_ (δ_H_ 7.77, 7.55 ppm) ligands, which integrated in 1:2:2 and 2:1 ratios, respectively. These data alongside the identification of two distinctive low field *ipso‐*carbon signals (δ_C_ 177.8, 175.7 ppm, identified via HMBC, Figure S29, Supporting Information) in the corresponding ^13^C{^1^H} NMR spectrum, thus, strongly supported the formation of [(^Dipp^BDI)Ca(Ph)(3,5‐*t*‐Bu_2_C_6_H_3_)Ca(^Dipp^BDI)] (**11**, Scheme 4). This deduction was validated by single crystal X‐ray diffraction, confirming the identity of **11** as a bis‐aryl bridged, but asymmetric, calcium β‐diketiminate complex (**Figure** **5**a).

The relative co‐planarity of the organocalcium core and the bulky 3,5‐*t*‐Bu_2_C_6_H_3_ substituent apparent in the structure of compound **10** is maintained (36.1°) in **11**. While this orientation is presumably enforced to reduce the mutual repulsion between the *N*‐Dipp substituents and the *tert*‐butyl groups, the less sterically encumbered phenyl group of **11** is orientated with an effectively orthogonal disposition relative to the calcium‐containing plane (87.29°).

The generality of this synthetic method was extended to an analogous reaction of compound **10** with (*p‐*tolyl)_2_ Hg. Analysis of this reaction after four hours by ^1^H NMR spectroscopy demonstrated the complete consumption of the calcium hydride starting material and the formation of a single new compound (**12**, Scheme 4). Compound **12** manifested the aromatic 3,5‐*t*‐Bu_2_C_6_H_3_ resonances in a respective 2:1 ratio by integration at δ_H_ 7.65 and 7.44 ppm. These signals appeared at higher field than those of the *p‐*tolyl ligand (δ_H_ 6.38 and 6.33), which were observed as mutually coupled doublets each integrating for two protons. As was observed in the spectrum of **11**, the two ^Dipp^BDI γ‐methine protons were resolved as a single resonance at 4.65 ppm. Although the spectrum evidenced significant asymmetry across the structure, the number and relative intensities of the remaining resonances were suggestive of a formulation of [(^Dipp^BDI)Ca(μ−3,5‐*t*‐Bu_2_C_6_H_3_)(μ‐*p‐*tolyl)Ca(^Dipp^BDI)] for compound **12**. This interpretation, which was also supported by the observation of two discriminated *ipso‐*carbon resonances at 178.0 (*t*‐Bu_2_C_6_H_3_) and 171.5 ppm (4‐Me‐C_6_H_4_) in the corresponding ^13^C{^1^H} NMR spectrum, was confirmed by the growth of X‐ray quality single crystals from a cooled saturated toluene solution (Figure 5b). Like its phenyl‐substituted analog (**11**), the *p‐*tolyl ligand of **12** adopts an approximately orthogonal orientation to the least squares plane defined by Ca1‐C30‐Ca2‐C44 (82.64°). This contrasts to the more in‐plane disposition of the 3,5‐*t*‐Bu_2_C_6_H_3_ unit (38.7°), the alignment of which is again apparently imposed by directional *ortho*‐C–H⋅⋅⋅Ca interactions.

**Scheme 5:**
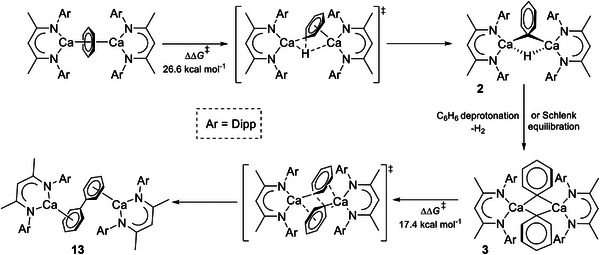
Harder and co‐workers’ calculated pathway leading to biphenyl dianion formation via compound **3**.^[^ ^15^ ^]^

We have previously reported that compound **3** reacts directly with bromobenzene and various aryl bromides to effect the uncatalyzed synthesis of biphenyl or the respective unsymmetrical biaryls (Scheme 1).^[^ ^12^ ^]^ In a further very recent advance, Harder and co‐workers have reported that ball milling of [(^Dipp^BDI)CaI(THF)] (and more sterically encumbered β‐diketiminate calcium and strontium analogs) with K/KI under solvent‐free conditions provided a deep purple product, assumed to be radicular [(^Dipp^BDI)Ca•(THF)].^[^ ^15^ ^]^ Although benzene solutions of this material were identified to contain compounds **1**∙THF and **3**, red‐brown crystals, which were observed to deposit from solution, were identified as [{(^Dipp^BDI)Ca(THF)}_2_(C_6_H_5_‐C_6_H_5_)] (**13**) in which two molecules of solvent had undergone a reductive and dehydrogenative coupling to provide a biphenyl dianion. Computational evaluation of the formation of **13** identified two potential mechanisms that invoked either i) direct attack of an initially formed C_6_H_6_^2−^ dianion of [{(^Dipp^BDI)Ca}_2_(C_6_H_6_)]^[^ ^9b^ ^]^ on neutral benzene (ΔΔ*G* ^‡^ = 26.0 kcal mol^−1^) or ii) an only marginally less viable (ΔΔ*G* ^‡^ = 26.6 kcal mol^−1^) isomerization of the C_6_H_6_^2−^ complex to afford the hydridophenylcalcium derivative (**2**). Notably, this latter mode of formation of **2** may be considered as the effective reverse (via **TS2**) of that earlier implicated in the transformation of **4_opt_** to **Int2** (Figure 2). Furthermore, Schlenk‐type redistribution of compound **2** was suggested as a viable source of **3**,^[^ ^15^, ^16^ ^]^ from which the formation of **13** is facilitated by a transition state (ΔΔ*G* ^‡^ = 17.4 kcal mol^−1^) in which the phenyl anions adopt twofold η^6^‐π‐interactions with each calcium (**Scheme** **5**). The authors recognized this latter mode of C−C bond formation to be reminiscent of our own earlier observations of C–C coupling and trienediide formation across the dimeric structures of various bis‐μ_2_‐η^2^‐bridged calcium acetylides.^[^ ^17^ ^]^ We have previously suggested that this reactivity is largely a consequence of π‐engagement of the C≡C bonds at each calcium and a resultant dissipation of negative charge from the otherwise mutually repulsive acetylide α‐C atoms.^[^ ^17^ ^]^

**Figure 6:**
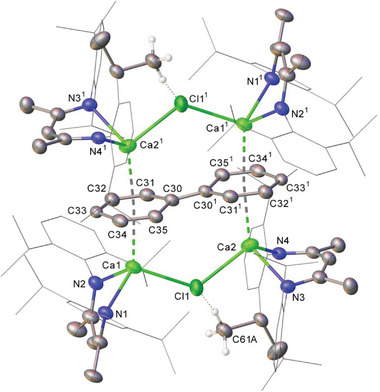
Molecular structure of **14** with thermal ellipsoids at the 50% probability level. hydrogen atoms, solvent and the second disordered component are omitted for clarity. *N*‐Dipp substituents are presented as wireframes (isopropyl groups containing C61A excepted), also for visual ease. Selected bond distances (Å) and bond angles (˚): Ca1‐Cl1 2.6289(14), Ca1‐N1 2.338(3), Ca1‐N2 2.355(3), Ca1‐C30 3.089(4), Ca1‐C31 2.854(4), Ca1‐C32 2.741(4), Ca1‐C33 2.769(3), Ca1‐C34 2.790(4), Ca1‐C35 2.903(4), Ca2‐Cl1 2.6442(14), Ca2‐N3 2.356(3), Ca2‐N4 2.364(3), Ca2‐C30^1^ 2.887(4), Ca2‐C31^1^ 2.960(4), Ca2‐C32^1^ 2.951(4), Ca2‐C33^1^ 2.932(3), Ca2‐C34^1^ 2.905(4), Ca2‐C35^1^ 2.926(4). Ca1‐Cl‐Ca2 120.59(5), Cl1‐Ca1‐C30 82.46(8), Ca1‐C33‐Ca2^1^ 123.87(13). Symmetry operations: ^1^ 2‐*x*, 1‐*y*, 1‐*z*.

In this regard, therefore, the calcium‐to‐aryl binding observed in compounds **4** and **9** and the previously reported derivative, [{(^Dipp^BDI)Ca}_2_(μ‐Br)(μ‐Ph)],^[^ ^12^ ^]^ confirm the viability of the polyhapto interactions implicated in the calculated transformation of **3** to **13**. Although we have observed no convincing evidence of the similar spontaneous transformation of compound **3** into **13**, we have noted that when stored for extended periods (>7 days), initially colorless benzene solutions of [{(^Dipp^BDI)Ca}Ph]_2_ (**3**) develop a red coloration. While such solutions evidence no significant changes in their NMR spectra, more extended periods of storage (>30 days) lead to the deposition of black crystals that are consequently insoluble in all solvents. X‐ray diffraction analysis of these crystals allowed the identification of this species as the chloride‐bridged and biphenyl dianion‐coordinated tetra‐calcium complex, [{{(^Dipp^BDI)Ca}_2_(μ_2_‐Cl)}_2_(C_6_H_5_‐C_6_H_5_)] (**14**, **Figure** **6**). Like compound **13**, the calcium centers of **14** are coordinated by a β‐diketiminate ligand and are assembled by η^6^‐coordination to each available C_6_‐face of a newly formed biphenyl dianion. In addition, and for necessary charge balance, the structure incorporates a further μ_2_‐bridging chloride anion [Ca1‐Cl1 2.6289(14), Ca2‐Cl1 2.6442(14) Å] between the calcium cations across each face of the planar organic dianion. As previously observed in structures such as that of **13** [1.390(6) Å],^[^ ^15^ ^]^ the C30‐C30' distance between the aromatic rings [1.354(8) Å] of **14** is considerably shorter than that observed within biphenyl itself [1.507 Å]^[^ ^18^ ^]^ and is more comparable to a C=C double bond. While the observation of biphenyl dianion formation from a solution of compound **2** could be interpreted to provide circumstantial corroboration of Harder and co‐workers’ tentative hypotheses, the chloride anions identified in the structure of **14** are presumably carried over as a spectroscopically unobservable artifact of the mercuric chloride employed in the synthesis of these phenyl calcium derivatives.

We cannot currently discount that the C–C coupling step leading to **14** could plausibly arise from an oxidative process induced by trace Hg(II) impurities in solution. Intrigued by the commonality of our observations and the earlier deductions of Harder and co‐workers, however, we re‐computed the formation of **13** starting from complex **3** at the same B3PW91‐D3 level of theory. The resultant Gibbs free energy (enthalpy) profile is presented in Figure S36 (Supporting Information). Unlike the profile reported by Harder and coworkers,^[^ ^15^ ^]^ in which the phenyl rings of the initial bis‐phenyl calcium dimer (**3'_opt_**, see Figure S37, Supporting Information) were positioned with a mutually orthogonal disposition, our starting compound (**3_opt_**), like the structurally characterized **3**, aligns both phenyl units in an almost coplanar orientation with the C_Ph_‐Ca‐C_Ph_‐Ca metallocycle. As shown in Figure S36 (Supporting Information), the transition state (**TS4**) corresponding to the nucleophilic attack of one Ph^−^ anion at the other Ph^−^ anion has a Gibbs free energy of 26.6 kcal mol^−1^ and the formation of the resulting biphenyl dianion Ca product (**13_opt_**) is an equilibrium reaction (Δ*G* = 2.1 kcal/mol relative to **3_opt_**). Taking into account that the presence of two coplanar phenyl units stabilizes compound **3_opt_** by 10.1 kcal mol^−1^ with respect to complex **3'**, our resultant kinetic barrier of 26.6 kcal mol^−1^ relative to **3_opt_** is, thus, perfectly concordant with the value of 17.4 kcal mol^−1^ relative to **3'_opt_** obtained by the group of Harder. As shown in Figure S36 (Supporting Information), transition state **TS4** is facilitated by a further isomer of **3_opt_ (3''_opt_)**, which comprises the two phenyl units aligned almost perpendicularly to the C_Ph_‐Ca‐C_Ph_‐Ca plane. Complex **3''_opt_** is located at 10.3 kcal mol^−1^, indicating that, while rotation of the first phenyl to form **3'_opt_** is energetically costly (Δ*G* = 10.1 kcal mol^−1^ relative to **3_opt_**), the rotation of the second phenyl constitutes an equilibrium process (see Figure S37, Supporting Information). On this basis, therefore, the formation of the biphenyl complex (**13**) may plausibly precede the formation of **14**, with the computed kinetic barrier of 26.6 kcal mol^−1^ accounting for the experimentally observed slow reaction time (>7days).

At this point, we considered how the C–C coupling across such bis‐phenyl calcium dimers may be facilitated by the establishment of polyhapto‐π‐interactions between each aromatic anion and the group 2 metal centers. Starting from **3** the achievement of the **TS4** geometry (Figure S36, Supporting Information) requires an adjustment of the initial η^1^‐engagement of the phenyl anion with calcium to η^6^. By analogy with our earlier rationalization of the C–C coupling across calcium acetylide dimers,^[^ ^17^ ^]^ we propose that the resultant π‐interactions with Ca induce a polarization across the C_6_ unit as a whole and decrease the mutual repulsion of the two carbanionic α‐carbon centers.

To further investigate this hypothesis, therefore, we carried out an Atoms in Molecules (AIM) analysis on species **3_opt_**, **3'_opt_**, **TS4** and **TS2**. As shown in Figure S38 (Supporting Information), the AIM analyses of compounds **3_opt_** and **3'_opt_** revealed bond critical points (BCPs) located between each Ca atom and both *ipso*‐C atoms of the two phenyl anions as well as a BCP located between the *ipso*‐C atoms of the two bridging phenyl rings. For **TS4**, on the other hand, the AIM analysis located two BCPs between each Ca atom and one *ipso*‐C of the phenyl anions. In corroboration of the presence of a polyhapto‐π interaction between the arene and the Ca (see Figure S39, Supporting Information), a BCP was also identified between the *ipso‐*C atoms of the two bridging phenyl ligands along with two cage critical points associated with each bridging phenyl ring and one Ca center.

While BCPs were also observed between the Ca atoms and the *ipso‐*C of the phenyl anion and the hydride ligand of **TS2**, the presence of a π interaction (see Figure S38, Supporting Information) enables a BCP between the *ipso‐*C atom of the phenyl and hydride ligands and between the phenyl ring and one Ca center. Significantly, while no BCP was detected between the phenyl ligand perpendicular to the C_Ph_‐Ca‐C_Ph_‐Ca plane and the Ca center for complex **3'_opt_**, for both **TS4** and **TS2** the η^6^ interaction between the phenyl anions and the calcium atoms is evidenced by a cage critical point. This suggests, therefore, that the effective nucleophilic attack of a Ph^−^ (or a H^−^) at the second Ph^−^ is likely to be assisted by a phenyl‐Ca π interaction, which perturbs the otherwise excessively high mutual repulsion between the two negatively charged *ipso‐*carbon (or *ipso‐*carbon and hydride) atoms, consequently enabling the resultant C–C or C–H coupling reaction.

## 3 Conclusions

In summary, we have reported a series of mixed hydrido‐aryl calcium complexes, where the disruption of the previously observed *ortho‐*CH‐to‐calcium interactions affords a tendency toward polyhapto‐coordination of the aromatic anion and isomerization to a benzyl species via an unusual “cross dimer” process involving hydride addition to the initially formed aryl substituent. In contrast, the introduction of moderate distal *meta‐* and *para‐*tolyl substitution leads to the maintenance of a more symmetric mode of Ca–C–Ca bridging, albeit with some variation in the relative orientation of the aryl substituents with respect to the Ca–Ca vector. Incorporation of the more sterically influential (3,5‐*t‐*Bu_2_C_6_H_5_) substituent allows selective isolation of a mixed hydrido‐aryl‐bridged calcium dimer. In turn, the availability of this species facilitates the first examples of mixed μ‐aryl‐μ‐aryl' bridged di‐calcium complexes. We have also observed the serendipitous formation of the tetra‐calcium complex, [{{(^Dipp^BDI)Ca}_2_(μ_2_‐Cl)}_2_(C_6_H_5_‐C_6_H_5_)], which comprises a biphenyl dianion. Although we have no solid experimental rationale for the mode of C–C bond formation, computational analysis suggests it is a consequence of the variable hapticity, which is a feature of the dimeric derivatives elaborated in this study. This work suggests, therefore, that such “cross dimer” polarization effects may provide the basis of a more generalizable route to both symmetric and unsymmetric biaryl units and related C‐X bonded organic products. We are continuing to explore these possibilities.

The Supporting Information contains Experimental details, NMR spectra, X‐ray crystallography, and computational details and atomic coordinates for the optimized geometries of the compounds.

Crystallographic data for all compounds have been deposited with the Cambridge Crystallographic Data Centre as supplementary publications CCDC 2260391–2260399 for **4**, **5**, **7**, **8**, **9**, **10**, **11**, **12** and **14**, respectively. These data are provided free of charge by the joint Cambridge Crystallographic Data Centre and Fachinformationszentrum Karlsruhe Access Structure sservice, www.ccdc.cam.ac.uk/structures.

## Conflict of Interest

The authors declare no conflict of interest.

## Supporting information

Supporting Information

Supporting Information

## Data Availability

The data that support the findings of this study are available in the supplementary material of this article.
