# Supplementary material for: Variable Ca‐Caryl Hapticity and its Consequences in Arylcalcium Dimers
Source: Adv Sci (Weinh). 2023 Sep 15;10(31):2304765. doi: 10.1002/advs.202304765 (PMC10625118; doi:10.1002/advs.202304765)
Supplement: Supplementary file 1 — Supporting Information [file ADVS-10-2304765-s002.pdf]

ADVANCED SCIENCE
Open Access

Supporting Information

for *Adv. Sci.*, DOI 10.1002/advs.202304765

Variable Ca-C_{aryl} Hapticity and its Consequences in Arylcalcium Dimers

Kyle G. Pearce, Chiara Dinoi, Ryan J. Schwamm, Laurent Maron, Mary F. Mahon* and Michael S. Hill**

General Considerations

All manipulations were carried out using standard Schlenk line and glovebox techniques under an inert atmosphere of argon. NMR experiments were conducted in J-Young's tap NMR tubes prepared in a glovebox. NMR spectra were recorded on a Bruker BioSpin GmbH spectrometer operating at 400.13 MHz (^{1}H) and 100.62 MHz (^{13}C). Elemental analyses were performed at Elemental Microanalysis Ltd., Okehampton, Devon, UK. Despite repeated attempts, apart from **11**, the sensitivity of the compounds reported herein defied all attempts to obtain microanalysis. Solvents were dried by passage through a commercially available solvent purification system and stored under argon in ampoules over 4 Å molecular sieves. C_6D_6 was purchased from Sigma-Aldrich, dried over potassium before distilling and storage over molecular sieves. $[(^{Dipp}BDI)CaH]_2$ (**1**), Ar_2Hg (Ar = Ph, *o*-tolyl, *m*-tolyl, *p*-tolyl, 3,5-*t*-$Bu_2C_6H_3$) and $[(^{Dipp}BDI)CaPh]_2$ (**3**) were synthesized according to literature procedures.[1-3]

Synthesis of [(^{Dipp}BDI)Ca(H)*o*-tolylCa(^{Dipp}BDI)] (4) and [(^{Dipp}BDI)Ca(H)(CH_2Ph)Ca(^{Dipp}BDI)] (5).

C_6D_6 (0.6 cm^3) was added to a J-Young's NMR tube containing $[(^{Dipp}BDI)CaH]_2$ (**1**) (30 mg, 0.03 mmol) and *o*-tolyl$_2$Hg (6.2 mg, 0.016 mmol), resulting in effervescence and the slow deposition of mercury metal over 48 hours. After the slow evaporation of C_6D_6, a small number of colorless crystals were grown from cold (–35 °C) toluene and subsequently pentane, allowing for the identification of [(^{Dipp}BDI)Ca(H)o-tolylCa(^{Dipp}BDI)] (**4**) and [(^{Dipp}BDI)Ca(H)benzylCa(^{Dipp}BDI)] (**5**), respectively. Yield: 21 mg. (**4**): ^{1}H NMR (C_6D_6): δ = 7.13-7.00 (^{Dipp}Ar-H, 12H), 6.79 (t, *o*-tolyl *m*- or *m'*-Ar-H, $^{3}J_{HH}$ = 6.78 Hz, 1H), 6.72 (d, *o*-tolyl *m*-Ar-C, $^{3}J_{HH}$ = 6.78 Hz, 1H), 6.60 (d, *o*-tolyl *m*- or *m'*-Ar-H, $^{3}J_{HH}$ = 7.54 Hz, 1H), 6.27 (t, *o*-tolyl *p*-Ar-H, $^{3}J_{HH}$ = 6.78 Hz, 1H), 4.74 (s, NC(CH_3)C$\underline{H}$, 2H), 4.59 (br s, Ca-$\underline{H}$, 1H), 3.01 (hept, C$\underline{H}$(CH_3)$_2$, $^{3}J_{HH}$ = 6.87 Hz, 8H), 2.11 (s, Ar-C$\underline{H}_3$, 3H), 1.58 (s, NC(C$\underline{H}_3$)CH, 12H), 1.14 (d, CH(C$\underline{H}_3$)$_2$, $^{3}J_{HH}$ = 6.9 Hz, 24H), 1.09 (d, CH(C$\underline{H}_3$)$_2$, $^{3}J_{HH}$ = 6.8 Hz, 24H). ^{13}C{^{1}H} NMR (C_6D_6) δ = 180.7 (*o*-tolyl *i*-Ar-C), 165.8 (N$\underline{C}$(CH_3)CH), 146.2 (^{Dipp}Ar-C), 142.0 (^{Dipp}Ar-C), 139.0 (*o*-tolyl *o*-Ar-C), 137.9 (*o*-tolyl *o'*-Ar-C, identified by HMBC), 129.3 (^{Dipp}Ar-C), 128.6 (*o*-tolyl *m or m'*-Ar-C), 127.0 (*o*-tolyl *m or m'*-Ar-C), 125.7 (*o*-tolyl *p*-Ar-C), 124.9 (^{Dipp}Ar-C), 124.4 (^{Dipp}Ar-C), 94.9 (NC(CH_3)$\underline{C}$H), 28.6 (CH($\underline{C}H_3$)$_2$), 24.8 (NC(C$\underline{H}_3$)CH), 24.2 (CH($\underline{C}H_3$)$_2$), 24.1 (CH($\underline{C}H_3$)$_2$). (**5**): ^{1}H NMR (C_6D_6): δ = 7.14-7.10 (m, Dipp-Ar-$\underline{H}$, 12H), 5.82 (dd, *o*-Ar-H, $^{3}J_{HH}$ = 6.84 Hz, 2H), 5.65 (d, *m*-Ar-H, $^{3}J_{HH}$ = 7.24 Hz, 2H), 5.55 (t, *p*-Ar-H, $^{3}J_{HH}$ = 6.84 Hz, 1H), 4.76 (s, NC(CH_3)C$\underline{H}$, 2H), 4.0 (s, Ca-$\underline{H}$, 1H), 3.10 (hept, C$\underline{H}$(CH_3)$_2$, $^{3}J_{HH}$ = 6.87 Hz, 8H), 1.90 (s, Benzyl -C$\underline{H}_2$, 2H), 1.67 (s, NC(C$\underline{H}_3$)CH, 12H), 1.22 (br, CH(C$\underline{H}_3$)$_2$, 48 H).

Isolation of [(^{Dipp}BDI)Ca(H)(*m*-tolyl)Ca(^{Dipp}BDI] (6) and [(^{Dipp}BDI)Ca(*m*-tolyl)]$_2$ (7). C_6D_6 (0.6 cm^3) was added to a J-Young's NMR tube containing $[(^{Dipp}BDI)CaH]_2$ (**1**) (20 mg, 0.02 mmol) and (*m*-tolyl)$_2$Hg (4.16 mg, 0.01 mmol), resulting in effervescence and the slow deposition of mercury metal. Colorless crystals of (**7**) were grown from the slow evaporation of C_6D_6. Crude Yield: 17 mg.

Rational Synthesis of [(^{Dipp}BDI)Ca(*m*-tolyl)]$_2$ (7).

[(^{Dipp}BDI)CaH]$_2$ (**1**) (20 mg, 0.02 mmol), *m*-tolyl$_2$Hg (10.82 mg, 0.02 mmol) and C_6D_6 (0.6 cm^3) were introduced into a J-Youngs NMR tube, resulting in the evolution of H_2 gas and the deposition of mercury metal. Colorless crystals were grown from the slow evaporation of a saturated C_6D_6 solution. Crystal Yield: 15 mg, 59%. ^{1}H NMR (C_6D_6): δ = 7.14-6.96 (m Ar-H, 18H), 6.87 (Ar-H, 2H), 4.86 (s, NC(CH$_3$)C$\underline{H}$,2H), 2.96 (hept, C$\underline{H}$(CH$_3$)$_2$, $^3J_{HH}$ = 6.83 Hz, 8H), 2.08 (s, Ar-C$\underline{H}_3$, 6H), 1.63 (s, NC(C$\underline{H}_3$)CH, 12H), 1.10 (d, CH(C$\underline{H}_3$)$_2$, $^3J_{HH}$ = 6.8 Hz, 24H), 0.75 (d, CH(C$\underline{H}_3$)$_2$, $^3J_{HH}$ = 6.8 Hz 24H). ^{13}C{^{1}H} NMR (C_6D_6) δ = 180.4 (*m*-tolyl *i*-Ar-C), 167.0 (N$\underline{C}$(CH$_3$)CH), 147.3 (Ar-C), 142.4 (Ar-C), 139.3 (Ar-C), 138.4 (Ar-C), 134.7 (Ar-C), 129.7 (Ar-C), 129.0 (Ar-C), 125.1 (Ar-C), 124.3 (Ar-C), 94.7 (NC(CH$_3$)$\underline{C}$H), 29.0 (CH($\underline{C}$H$_3$)$_2$), 25.3 (CH($\underline{C}$H$_3$)$_2$), 25.0 (NC(C$\underline{H}_3$)CH), 24.8 (CH($\underline{C}$H$_3$)$_2$), 22.0 (Ar-$\underline{C}$H$_3$).

Synthesis of [(^{Dipp}BDI)Ca(*p*-tolyl)]$_2$ (8).

[(^{Dipp}BDI)CaH]$_2$ (**1**) (28.8 mg, 0.031 mmol), *p*-tolyl$_2$Hg (12 mg, 0.031 mmol) and C_6D_6 (0.6 cm^3) were introduced into a J-Young's NMR tube, resulting in the evolution of H_2 gas and the deposition of mercury metal, taking 48 hours for complete conversion. After the slow evaporation of C_6D_6, the minimum amount of hexane (ca. 1 cm^3) was added and colorless crystals of **8** were grown at –35 °C. Crystal Yield: 11 mg, 32%. ^{1}H NMR (C_6D_6): δ = 7.24 (d, *p*-tolyl Ar-H, $^3J_{HH}$ = 7.08 Hz, 2H), 7.18 (s, Ar-H, 4H), 7.12 (d, Ar-H, $^3J_{HH}$ = 7.08 Hz, 3H), 7.07 (s, Ar-H, 3H), 7.05 (s, Ar-H, 2H), 7.03-7.69 (m, Ar-H, 3H), 6.83 (d, *p*-tolyl Ar-C Ar-H, $^3J_{HH}$ = 6.84 Hz, 2H), 6.44 (d, *p*-tolyl Ar-H, $^3J_{HH}$ = 6.84 Hz, 1H), 4.86 (s, NC(CH$_3$)C$\underline{H}$, 1H), 4.77 (s, NC(CH$_3$)C$\underline{H}$, 1H), 3.09 (hept, C$\underline{H}$(CH$_3$)$_2$, $^3J_{HH}$ = 6.84 Hz, 4H), 2.91 (hept, C$\underline{H}$(CH$_3$)$_2$, $^3J_{HH}$ = 6.84 Hz, 4H), 2.11 (s, Ar-CH$_3$, 6H), 1.66 (s, NC(C$\underline{H}_3$)CH, 6H), 1.63 (s, NC(C$\underline{H}_3$)CH, 6H), 1.21 (d, CH(C$\underline{H}_3$)$_2$, $^3J_{HH}$ = 6.98 Hz 12H), 1.14 (d, CH(C$\underline{H}_3$)$_2$, $^3J_{HH}$ = 6.69 Hz 12H), 1.10 (d, CH(C$\underline{H}_3$)$_2$, $^3J_{HH}$ = 6.69 Hz 12H), 0.67 (d, CH(C$\underline{H}_3$)$_2$, $^3J_{HH}$ = 6.98 Hz 12H). ^{13}C{^{1}H} NMR (C_6D_6) δ = 189.2 (*p*-tolyl *i*-Ar-C), 172.1 (*p*-tolyl *i*-Ar-C), 166.2 (N$\underline{C}$(CH$_3$)CH), 165.7 (N$\underline{C}$(CH$_3$)CH), 146.2 (Ar-C), 146.15 (Ar-C), 142.3 (Ar-C), 141.6 (Ar-C), 141.5 (Ar-C), 137.1 (Ar-C), 136.6 (Ar-C), 129.7 (*p*-tolyl Ar-C), 128. 9 (*p*-tolyl Ar-C), 128.5 (Ar-C), 128.1 (Ar-C), 125.3 (Ar-C), 124.5 (Ar-C), 124.2 (Ar-C), 123.7 (Ar-C), 123.5 (Ar-C),93.9 (NC(CH$_3$)$\underline{C}$H), 92.9 (NC(CH$_3$)$\underline{C}$H), 28.3 ($\underline{C}$H(CH$_3$)$_2$), 28.2 ($\underline{C}$H(CH$_3$)$_2$), 24.5 (CH($\underline{C}$H$_3$)$_2$), 24.4 (NC(C$\underline{H}_3$)CH), 24.2 (CH($\underline{C}$H$_3$)$_2$), 24.0 (CH($\underline{C}$H$_3$)$_2$), 23.9 (CH($\underline{C}$H$_3$)$_2$), 20.8 (Ar-CH$_3$), 20.3 (NC(C$\underline{H}_3$)CH).

Synthesis of [(^{Dipp}BDI)Ca(H)(3,5-*t*-Bu$_2$C$_6$H$_3$)Ca(^{Dipp}BDI)] (10).

C_6D_6 (0.6 cm^3) was added to a J-Young's NMR tube containing [(^{Dipp}BDI)CaH]$_2$ (**1**) (15 mg, 0.016 mmol) and ((3,5-*t*-Bu$_2$C$_6$H$_3$)$_2$Hg (4.72 mg, 0.08 mmol), resulting in effervescence and the slow deposition of mercury metal over 72 hours. After the slow evaporation of C_6D_6, the minimum amount of C_6H_6 was added, resulting in the deposition of colorless crystals of **10**. Crystal Yield: 13 mg, 73%.

^{1}H NMR (C_6D_6): δ = 7.55 (br m, (3,5-tBu)$_2$Ph *p*-Ar-H, 1H), 7.46 (d, (3,5-tBu)$_2$Ph *o*-Ar-H, $^3J_{HH}$ = 1.71 Hz, 2H), 7.37 (t, ^{Dipp}Ar-H, $^3J_{HH}$ = 1.69 Hz, 1H), 7.23-7.20 (m, ^{Dipp}Ar-H, 2H), 7.12-7.05 (m, ^{Dipp}Ar-H, 9H), 4.76 (s, NC(CH$_3$)C$\underline{H}$, 2H), 4.58 (s, Ca-H, 1H), 3.12 (br, C$\underline{H}$(CH$_3$)$_2$, 8H), 1.61 (s, NC(C$\underline{H}_3$)CH, 12H), 1.41 (s, Ar-^{t}Bu, 9H), 1.29 (s, Ar-^{t}Bu, 9H), 1.20 (d, CH(C$\underline{H}_3$)$_2$, $^3J_{HH}$ = 7.15 Hz, 24H). ^{13}C{^{1}H} NMR (C_6D_6) δ = 177.7 ((3,5-^{t}Bu)$_2$Ph *i*-Ar-C), 166.5 (N$\underline{C}$(CH$_3$)CH), 150.8 (Ar-C), 150.2 (Ar-C), 144.4 (Ar-C), 142.3 (Ar-C), 142.1 (Ar-C), 129.2 (Ar-C), 128.6 (Ar-C), 124.8 (^{Dipp}Ar-C), 124.0 ((3,5-^{t}Bu)$_2$Ph-Ar-C), 122.9 (^{Dipp}Ar-C), 122.4 (^{Dipp}Ar-C), 94.7 (NC(CH$_3$)$\underline{C}$H), 34.9 (Ar-tBu $\underline{C}$(CH$_3$)$_3$), 31.7 (Ar-^{t}Bu C($\underline{C}$H$_3$)$_3$), 31.6 (Ar-^{t}Bu C($\underline{C}$H$_3$)$_3$), 24.34 (CH($\underline{C}$H$_3$)$_2$), 24.28 (NC($\underline{C}$H$_3$)CH).

Synthesis of [(^{Dipp}BDI)Ca(Ph)(3,5-*t*-Bu$_2$C$_6$H$_3$)Ca(^{Dipp}BDI)] (11).

Ph$_2$Hg (2.90 mg, 0.008 mmol), compound **10** (17.7 mg, 0.016 mmol) and C_6D_6 (0.6 cm^3) were introduced into a J-Young's NMR tube, resulting in effervescence and the slow deposition of mercury metal over 16 hours. The reaction mixture was filtered into a vial and colorless crystals of **11** were grown from the saturated solution. Crystal Yield: 9.8 mg, 52%. ^{1}H NMR (C_6D_6): δ = 7.77 (d, (3,5-tBu)$_2$Ph *o*-Ar-H, $^3J_{HH}$ = 1.63 Hz, 2H), 7.55 (br m, (3,5-tBu)$_2$Ph *p*-Ar-H, 1H), 7.46 (br m, ^{Dipp}Ar-H, 1H), 7.23-7.18 (m, ^{Dipp}Ar-H, 4H), 7.13-7.05 (m, ^{Dipp}Ar-H, 8H), 6.91 (t, C$_6$H$_5$ *p*-Ar-C, $^3J_{HH}$ = 7.14 Hz, 1H), 6.65 (t, C$_6$H$_5$, *m*-Ar-H, $^3J_{HH}$ = 7.28 Hz, 2H), 6.54 (br m, C$_6$H$_5$, *o*-Ar-H, 2H), 4.77 (s, NC(CH$_3$)C$\underline{H}$, 2H), 3.05 (m, C$\underline{H}$(CH$_3$)$_2$, 4H), 2.73 (hept, C$\underline{H}$(CH$_3$)$_2$, $^3J_{HH}$ = 6.84 Hz, 4H), 1.59 (s, NC(C$\underline{H}_3$)CH, 12H), 1.52 (s, NC(C$\underline{H}_3$)CH, 12H), 1.29 (s, Ar-^{t}Bu, 18H), 1.19 (d, CH(C$\underline{H}_3$)$_2$, $^3J_{HH}$ = 6.55 Hz, 12H), 0.97 (d, CH(C$\underline{H}_3$)$_2$, $^3J_{HH}$ = 6.55 Hz, 12H), 0.87 (br d, CH(C$\underline{H}_3$)$_2$, $^3J_{HH}$ = 6.55 Hz, 12H), 0.34 (d, CH(C$\underline{H}_3$)$_2$, $^3J_{HH}$ = 6.55 Hz, 12H). ^{13}C{^{1}H} NMR (C_6D_6) δ = 177.8 ((3,5-^{t}Bu)$_2$Ph *i*-Ar-C), 175.7 (C$_6$H$_5$ *i*-Ar-C), 166.4 (N$\underline{C}$(CH$_3$)CH),150.8 (Ar-C), 150.5 (Ar-C), 146.4 (^{Dipp}Ar-C), 142.3 (^{Dipp}Ar-C), 141.8 (^{Dipp}Ar-C), 139.6 (C$_6$H$_5$, *o*-Ar-C), 130.4 ((3,5-tBu)$_2$Ph *o*-Ar-C), 128.8 (C$_6$H$_5$, *m*-Ar-C), 128.6 (^{Dipp}Ar-C), 124.6 (^{Dipp}Ar-C), 124.1 (^{Dipp}Ar-C), 123.8 (^{Dipp}Ar-C), 122.9 (^{Dipp}Ar-C), 122.2 ((3,5-tBu)$_2$Ph *p*-Ar-H), 94.8 (NC(CH$_3$)$\underline{C}$H), 35.06 (C(CH$_3$)$_2$), 34.9 (Ar-^{t}Bu $\underline{C}$(CH$_3$)$_3$), 31.8 (NC($\underline{C}$H$_3$)CH), 31.7 (Ar-^{t}Bu C($\underline{C}$H$_3$)$_3$), 29.0 (NC($\underline{C}$H$_3$)CH), 27.9 (NC($\underline{C}$H$_3$)CH), 25.4 (CH($\underline{C}$H$_3$)$_2$), 24.8(CH($\underline{C}$H$_3$)$_2$), 24.7 (), 24.2 (CH($\underline{C}$H$_3$)$_2$), 23.4 (CH($\underline{C}$H$_3$)$_2$). Anal. Calc. for Ca$_2$N$_4$C$_{64}$H$_{88}$ -: C, 79.27; H, 9.21; N, 4.74. Found: C, 79.07; H, 8.68; N, 4.87.

Synthesis of of [(^{Dipp}BDI)Ca(μ-3,5-*t*-Bu$_2$C$_6$H$_3$)(μ-*p*-tolyl)Ca(^{Dipp}BDI)] (12).

p-tolyl$_2$Hg (3.12 mg, 0.008 mmol) was added to a benzene solution of **10** (17.7 mg, 0.016 mmol), resulting in effervescence and the slow deposition of mercury metal over 4 hours. The reaction mixture was filtered into a vial and evaporated to almost dryness, the minimum amount of toluene was added cooled to –35 °C, affording colorless crystals of **12**. Crystal Yield: 12.2 mg, 64%. ^{1}H NMR (C_6D_6): δ = 7.65 (d, (3,5-tBu)$_2$Ph *o*-Ar-H, $^3J_{HH}$ = 1.56 Hz, 2H), 7.44 (br m, (3,5-tBu)$_2$Ph *p*-Ar-H 1H), 7.35 (br m, ^{Dipp}Ar-H, 1H), 7.15-7.07 (m, ^{Dipp}Ar-H, 5H), 6.97-6.94 (m, ^{Dipp}Ar-H, 6H), 6.38 (d, *p*-tolyl Ar-H, $^3J_{HH}$ = 7.14 Hz, 2H), 6.33 (d, *p*-tolyl Ar-H, $^3J_{HH}$ = 7.14 Hz, 2H), 4.65 (s, NC(CH$_3$)C$\underline{H}$, 2H), 2.93 (m, C$\underline{H}$(CH$_3$)$_2$,

4H), 2.62 (m, C<u>H</u>(CH_3)$_2$, $^3J_{HH}$ = 6.80 Hz, 4H), 2.03 (s, Ar-C<u>H</u>$_3$, 3H), 1.49 (s, NC(C<u>H</u>$_3$)CH, 12H), 1.18 (s, Ar-^{t}Bu, 18H), 1.09 (d, CH(C<u>H</u>$_3$)$_2$, $^3J_{HH}$ = 6.32 Hz, 12H), 0.87 (d, CH(C<u>H</u>$_3$)$_2$, $^3J_{HH}$ = 6.32 Hz, 12H), 0.75 (br, CH(C<u>H</u>$_3$)$_2$, 12H), 0.23 (br d, CH(C<u>H</u>$_3$)$_2$, $^3J_{HH}$ = 7.37 Hz 12H). ^{13}C{^{1}H} NMR (C_6D_6) δ = 178.0 ((3,5-^{t}Bu)$_2$Ph *i*-Ar-C), 171.5 (*p*-tolyl *i*-Ar-C), 166.4 (N<u>C</u>(CH_3)CH), 150.8 (^{Dipp}Ar-C), 150.4 (^{Dipp}Ar-C), 146.4 (^{Dipp}Ar-C), 142.3 (^{Dipp}Ar-C), 141.7 (^{Dipp}Ar-C), 139.5 (*p*-tolyl Ar-C), 136.4 (^{Dipp}Ar-C), 130.4 ((3,5-^{t}Bu)$_2$Ph *o*-Ar-C), 129.8 (*p*-tolyl Ar-C), 124.6 (^{Dipp}Ar-C), 124.1 (^{Dipp}Ar-C), 123.7 (^{Dipp}Ar-C), 122.9 (^{Dipp}Ar-C), 122.2 ((3,5-^{t}Bu)$_2$Ph *p*-Ar-C), 94.7 (NC(CH_3)<u>C</u>H), 35.1 (Ar-<u>C</u>(CH_3)$_3$), 31.8 (NC(<u>C</u>H$_3$)CH), 31.7 (NC(<u>C</u>H$_3$)CH), 29.0 (CH(<u>C</u>H$_3$)$_2$), 27.9 (CH(<u>C</u>H$_3$)$_2$), 25.4 (Ar-<u>C</u>(CH_3)$_3$), 24.8 (CH(<u>C</u>H$_3$)$_2$), 24.7 (CH(<u>C</u>H$_3$)$_2$), 24.2 (CH(<u>C</u>H$_3$)$_2$), 23.2 (CH(<u>C</u>H$_3$)$_2$), 21.5 (Ar-<u>C</u>H$_3$).

Isolation of [{(^{Dipp}BDI)Ca}$_2$(μ$_2$-Cl)}$_2$(C_6H_5-C_6H_5)] (14).

C_6D_6 (0.6 cm^3) was added to a vial containing [(^{Dipp}BDI)CaH]$_2$ (**1**) (30 mg, 0.03 mmol) and Ph_2Hg (16 mg, 0.045 mmol), resulting in effervescence and the slow deposition of mercury. Storage of the solution for an extended period of time (7 days), resulted in a red colouration. After 30 days, the formation of deep yellow (appear black) crystals was observed. The isolated crystals were insoluble but could be crystallographically identified as **14**.

NMR Spectra

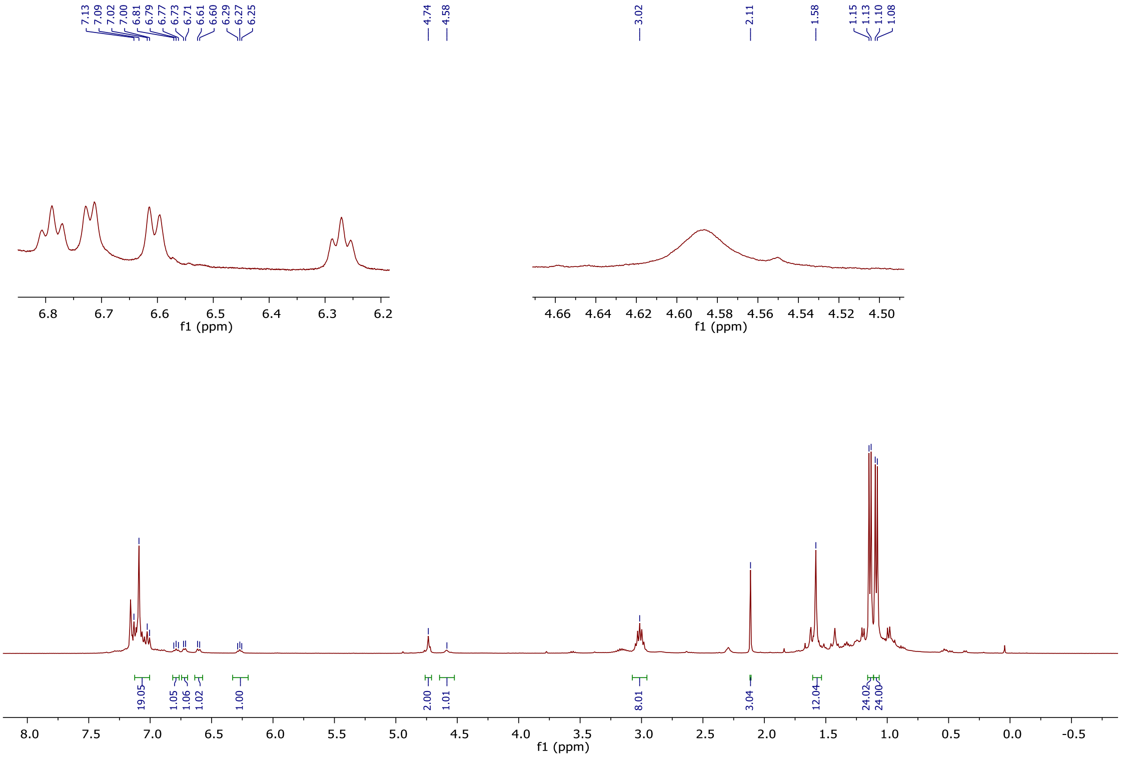

Figure S1. *^{1}H NMR Spectrum (C_6D_6, 298 K, 400.13 MHz) for [(^{Dipp}BDI)Ca(H)o-tolylCa(^{Dipp}BDI)]* ***(4)***.

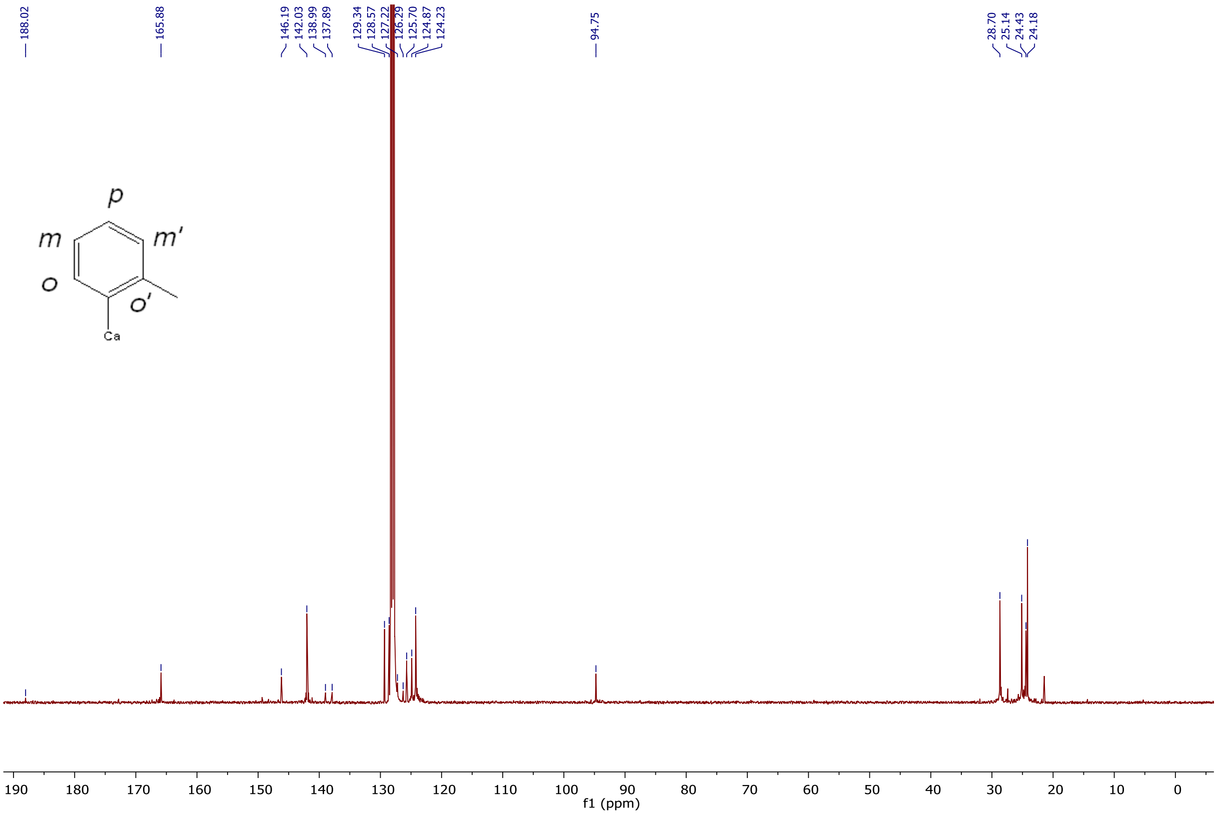

Figure S2. *^{13}C{^{1}H} NMR Spectrum (C_6D_6, 298 K, 100.62 MHz) for [(^{Dipp}BDI)Ca(H)o-tolylCa(^{Dipp}BDI)]* ***(4)***.

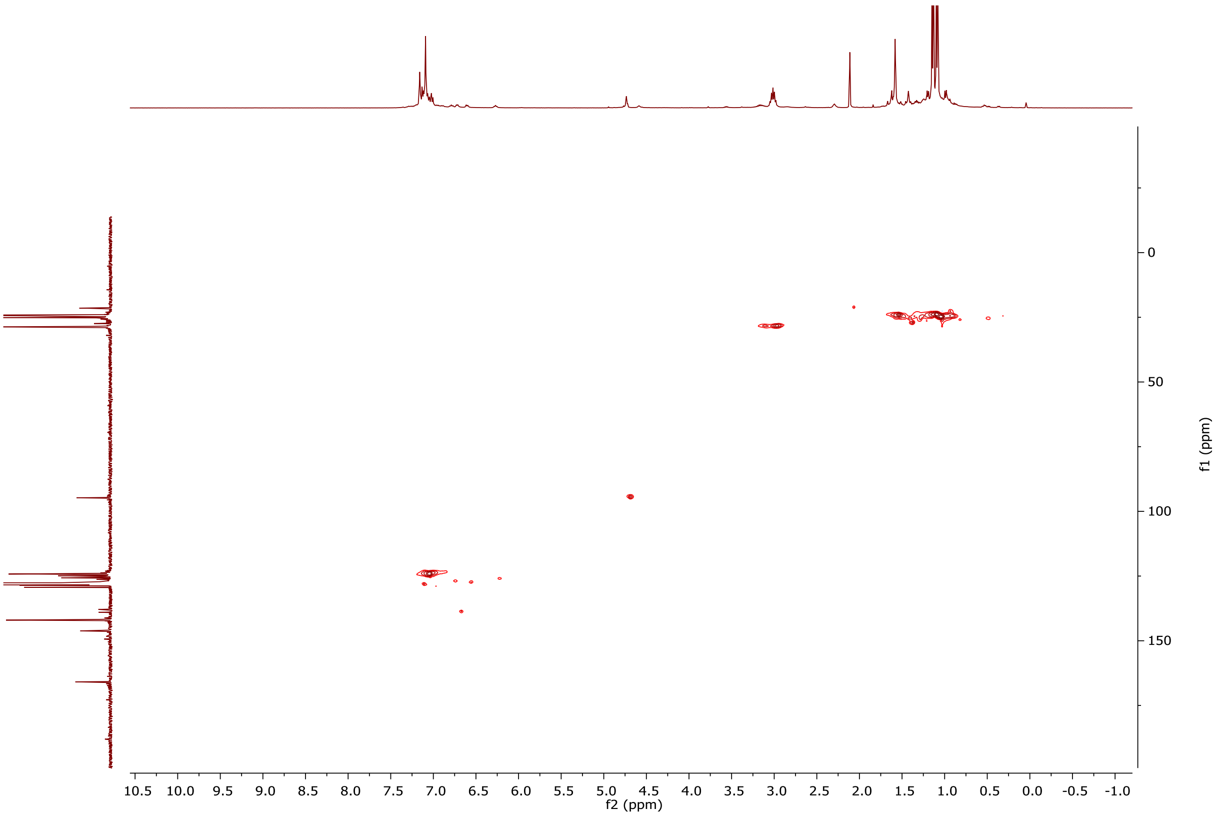

Figure S3. *^{1}H-^{13}C HSQC trace (C_6D_6, 298 K, 400.13, 100.62 MHz) for [(^{Dipp}BDI)Ca(H)*o-tolyl*Ca(^{Dipp}BDI)] (**4**).*

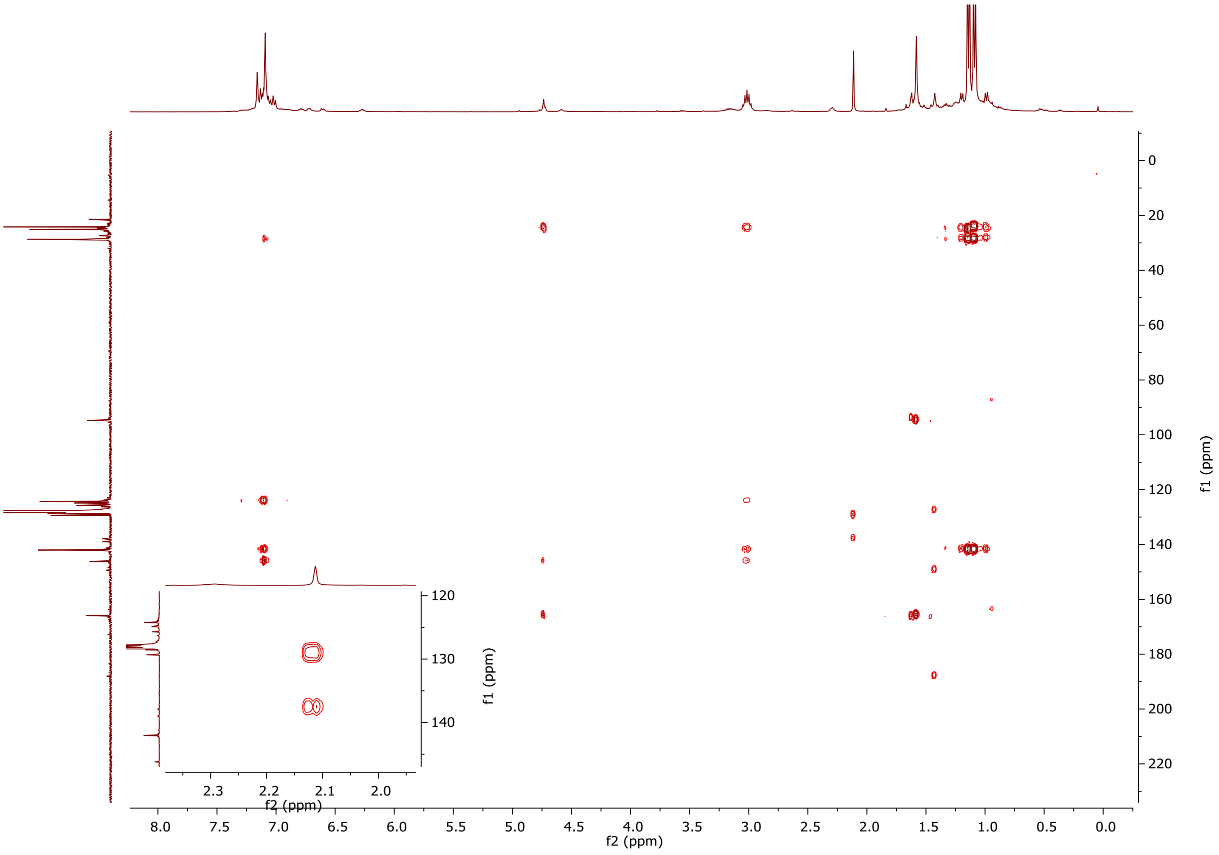

Figure S4. *^{1}H-^{13}C HMBC trace (C_6D_6, 298 K, 400.13, 100.62 MHz) for [(^{Dipp}BDI)Ca(H)*o-tolyl*Ca(^{Dipp}BDI)] (**4**).*

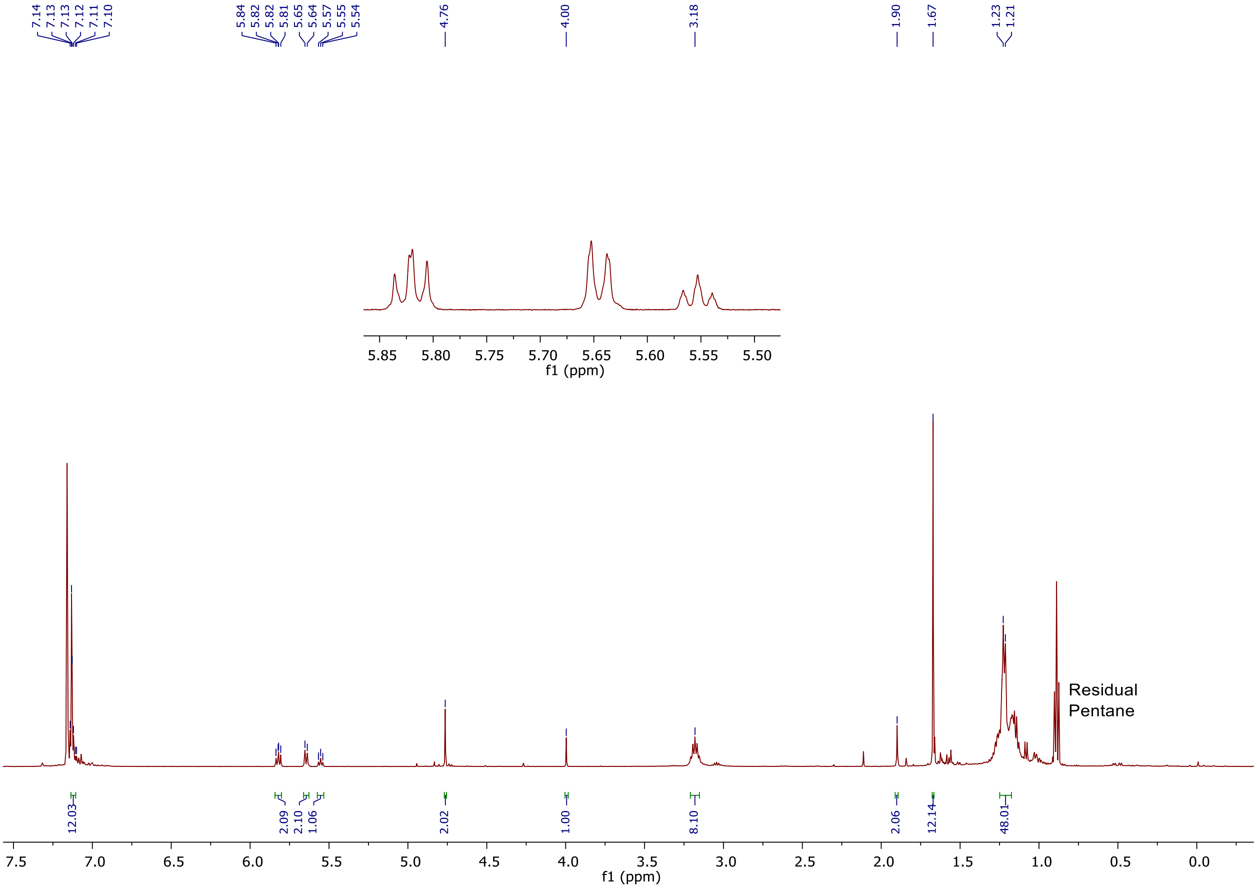

Figure S5. *^{1}H NMR Spectrum (C_6D_6, 298 K, 400.13 MHz) for [(^{Dipp}BDI)Ca(H)(CH_2Ph)Ca(^{Dipp}BDI)] (**5**).*

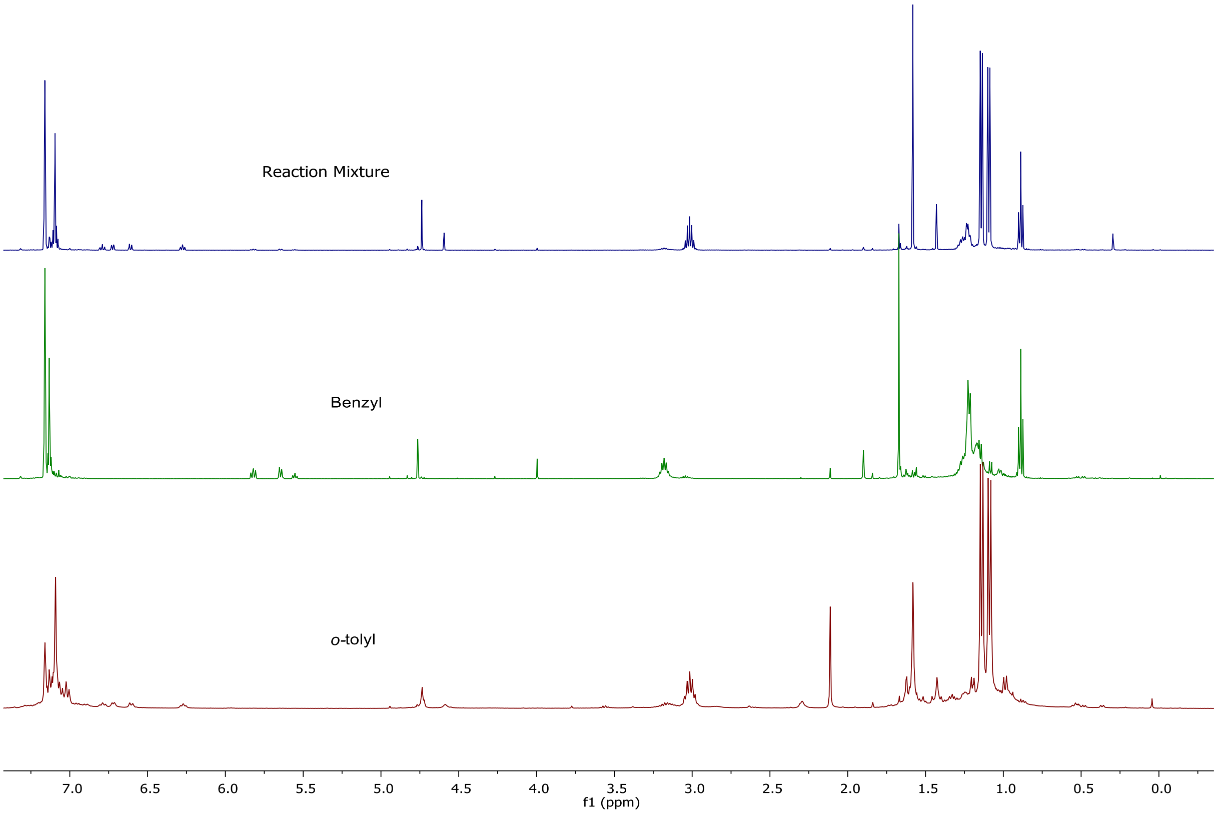

Figure S6. *^{1}H NMR Spectrum (C_6D_6, 298 K, 400.13 MHz) of the reaction between [LCaH]$_2$ and o-tolyl$_2$Hg, showing the presence of both [(^{Dipp}BDI)Ca(H)o-tolylCa(^{Dipp}BDI)] (**4**) and [(^{Dipp}BDI)Ca(H)(CH_2Ph)Ca(^{Dipp}BDI)] (**5**)..*

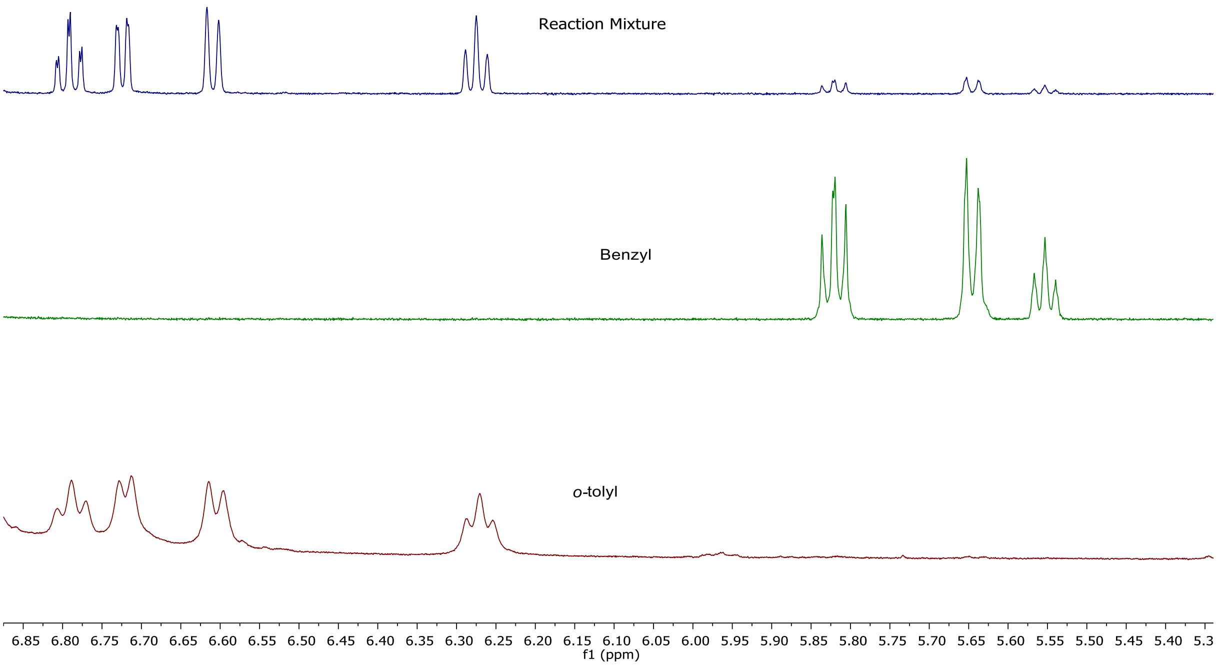

Figure S7. *^{1}H NMR Spectrum (C_6D_6, 298 K, 400.13 MHz) of the reaction between $[LCaH]_2$ and o-tolyl$_2$Hg, zoomed in on the aromatic region to demonstrate the presence of both [(^{Dipp}BDI)Ca(H)*o-tolyl*Ca(^{Dipp}BDI)] (**4**) and [(^{Dipp}BDI)Ca(H)(CH_2Ph)Ca(^{Dipp}BDI)] (**5**).*.

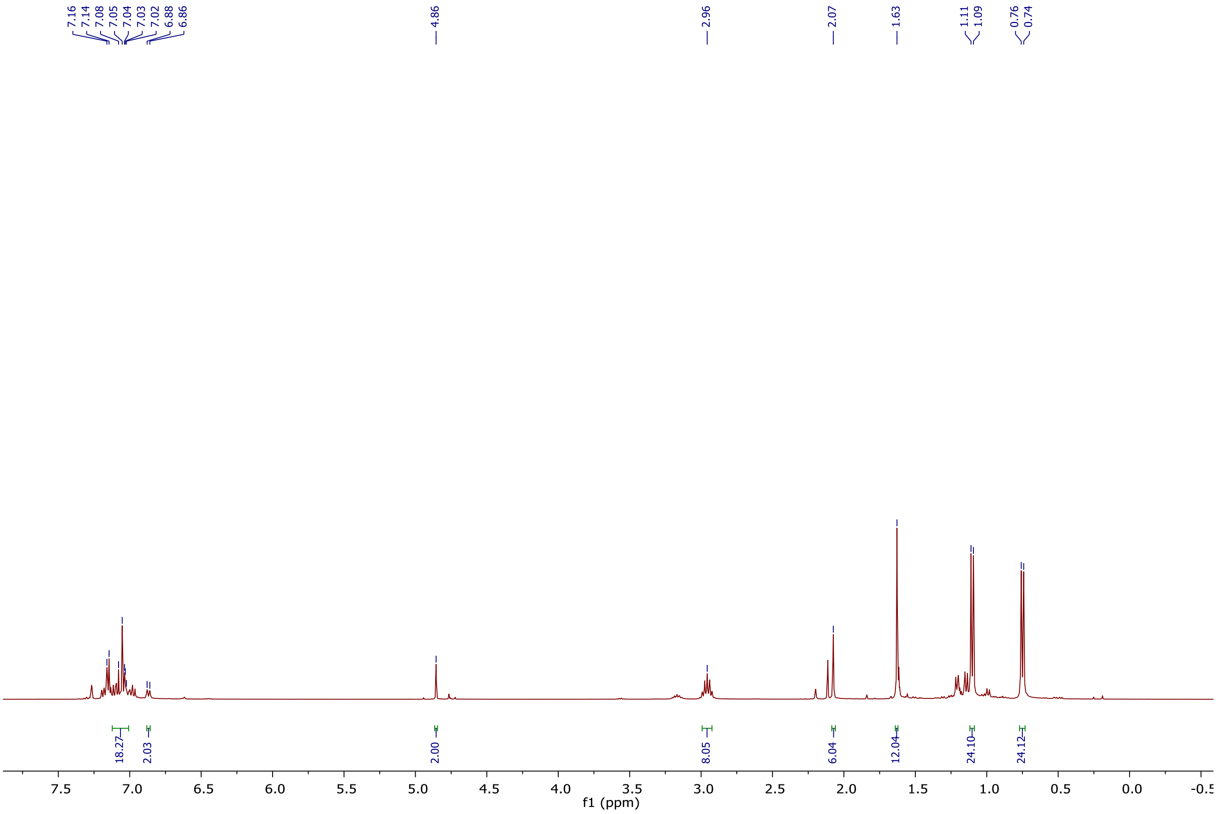

Figure S8. *^{1}H NMR Spectrum (C_6D_6, 298 K, 400.13 MHz) for [(^{Dipp}BDI)Ca(m-tolyl)]$_2$ (**7**).*

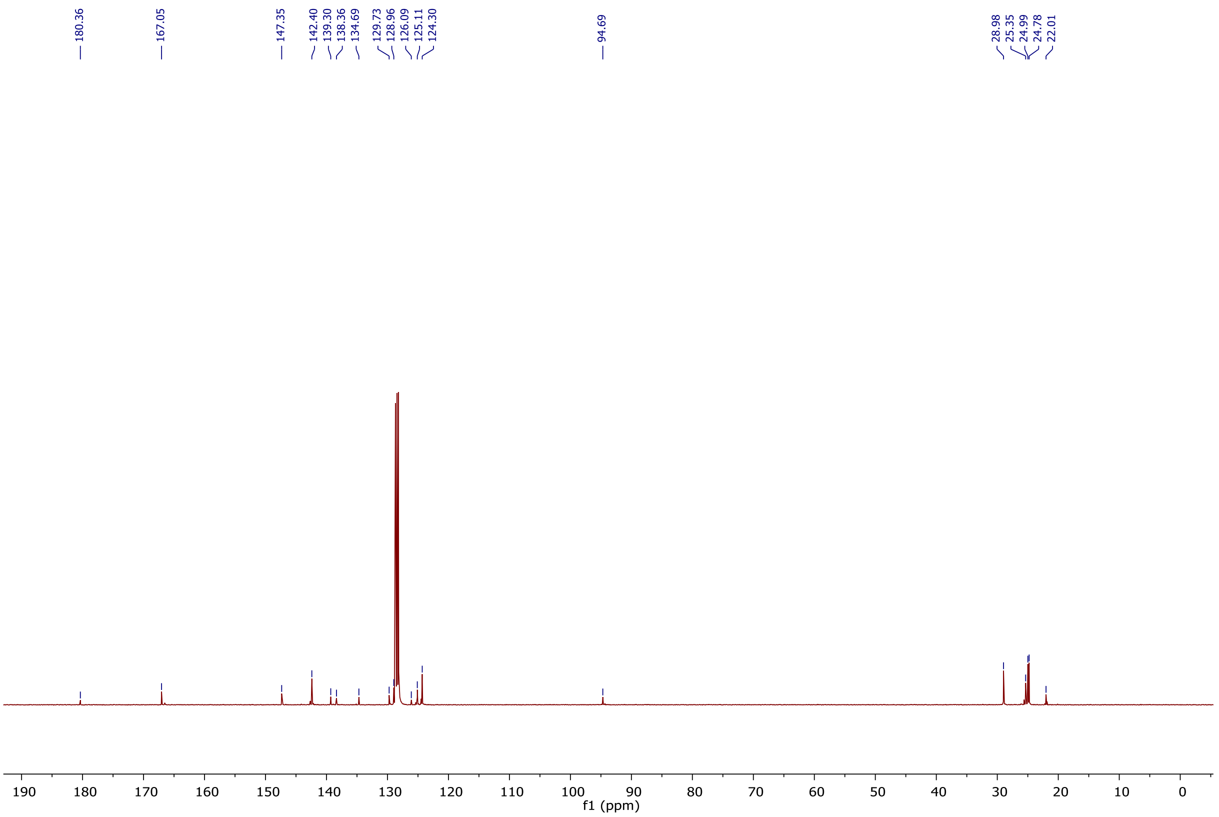

Figure S9. *$^{13}C\{^{1}H\}$ NMR Spectrum (C_6D_6, 298 K, 100.62 MHz) for [(^{Dipp}BDI)Ca(m-tolyl)]$_2$ (**7**).*

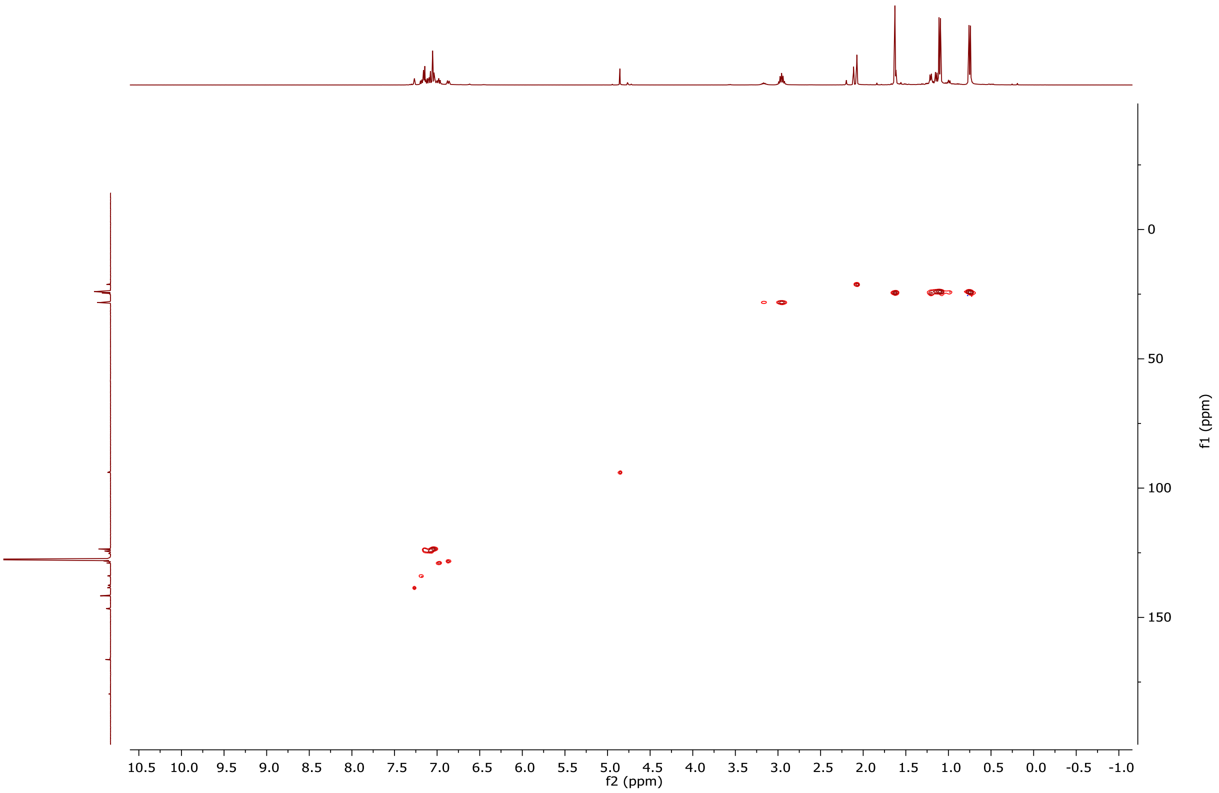

Figure S10. *^{1}H-^{13}C HSQC trace (C_6D_6, 298 K, 400.13, 100.62 MHz) for [(^{Dipp}BDI)Ca(m-tolyl)]$_2$ (**7**).*

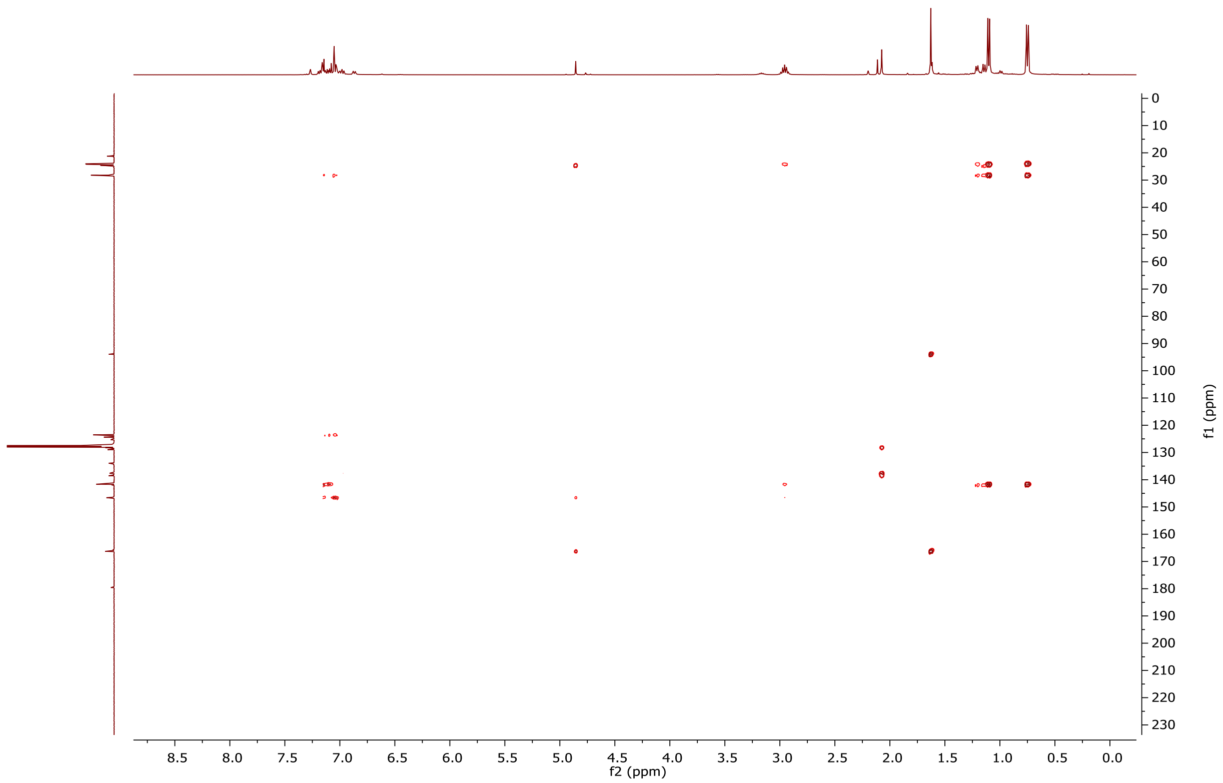

Figure S11. *^{1}H-^{13}C HMBC trace (C_6D_6, 298 K, 400.13, 100.62 MHz) for [(^{Dipp}BDI)Ca(m-tolyl)]$_2$ (**7**).*

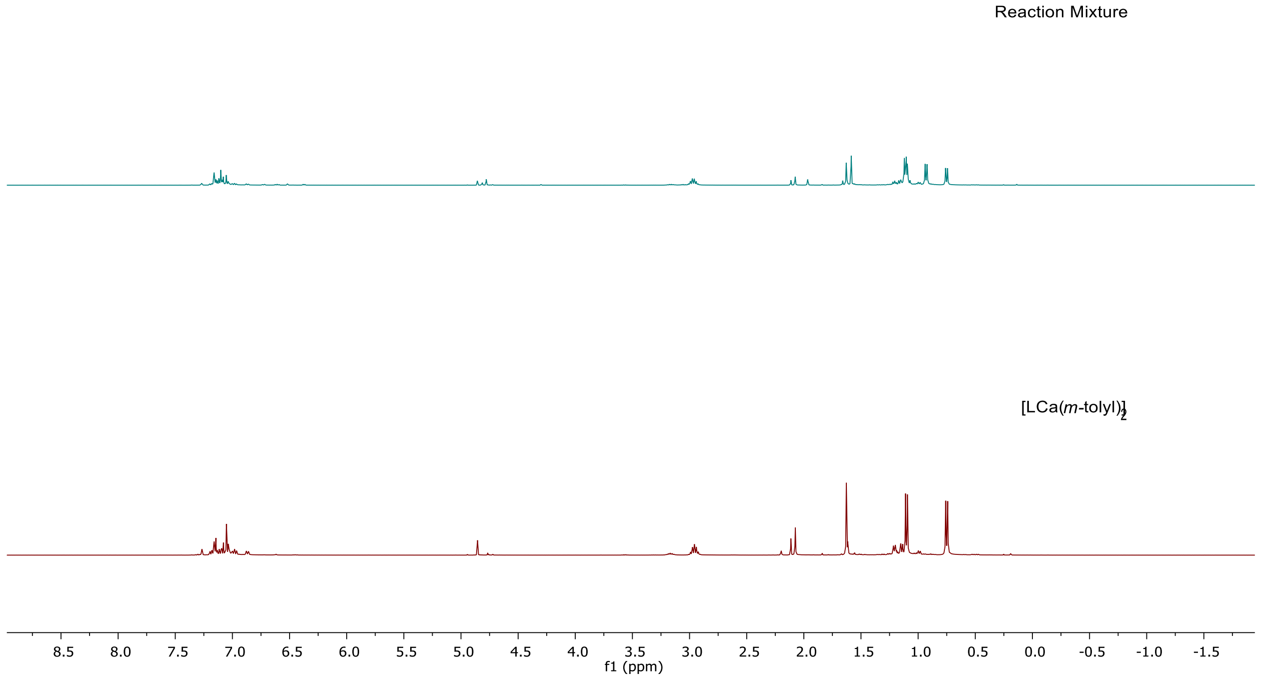

Figure S12. *^{1}H NMR Spectrum (C_6D_6, 298 K, 400.13 MHz) of the reaction between [(^{Dipp}BDI)CaH]$_2$ and 0.5 equiv. of (m-tolyl$_2$)Hg.*

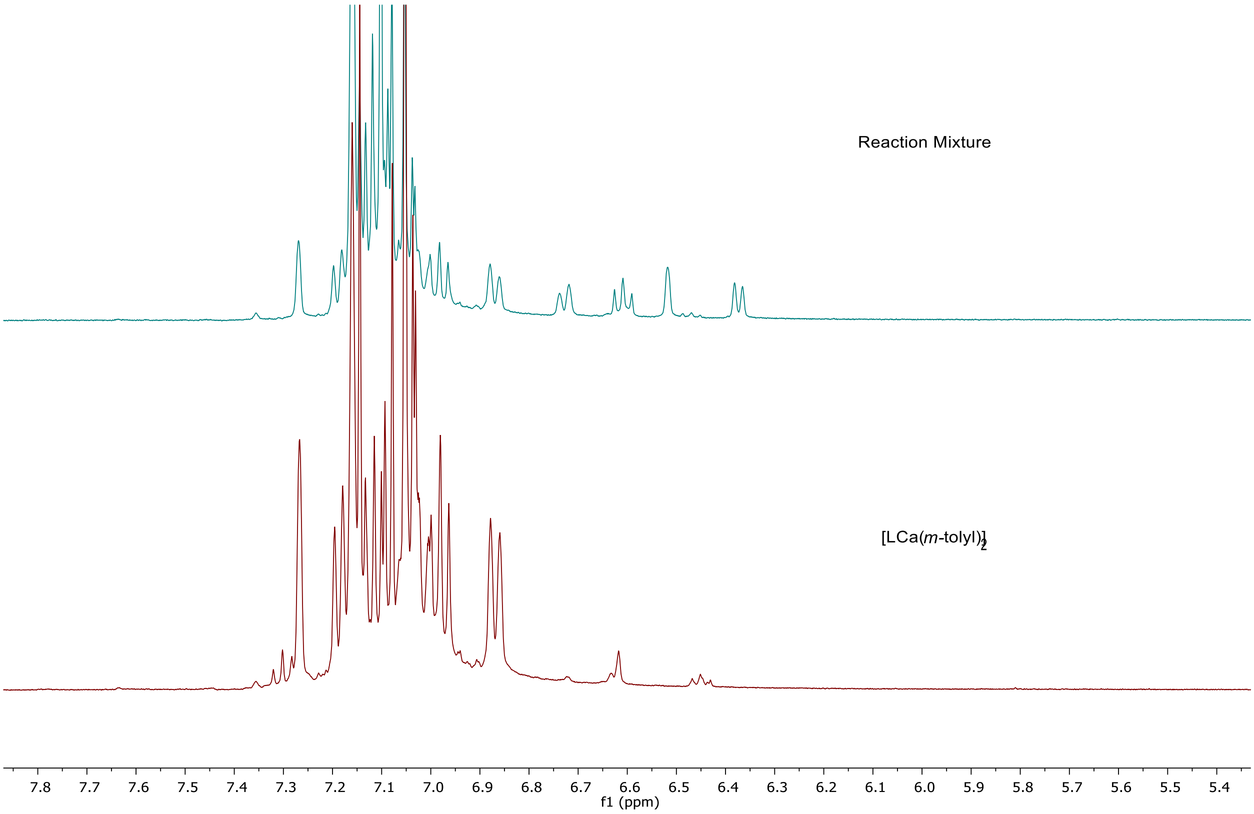

Figure S13. *^{1}H NMR Spectrum (C_6D_6, 298 K, 400.13 MHz) of the reaction between $[LCaH]_2$ and 0.5 equiv. of m-tolyl$_2$Hg, zoomed in on the aromatic region to demonstrate the presence of both $[(^{Dipp}BDI)Ca(H)m\text{-}tolylCa(^{Dipp}BDI)]$ (**6**) and $[(^{Dipp}BDI)Ca(m\text{-}tolyl)]_2$ (**7**).*

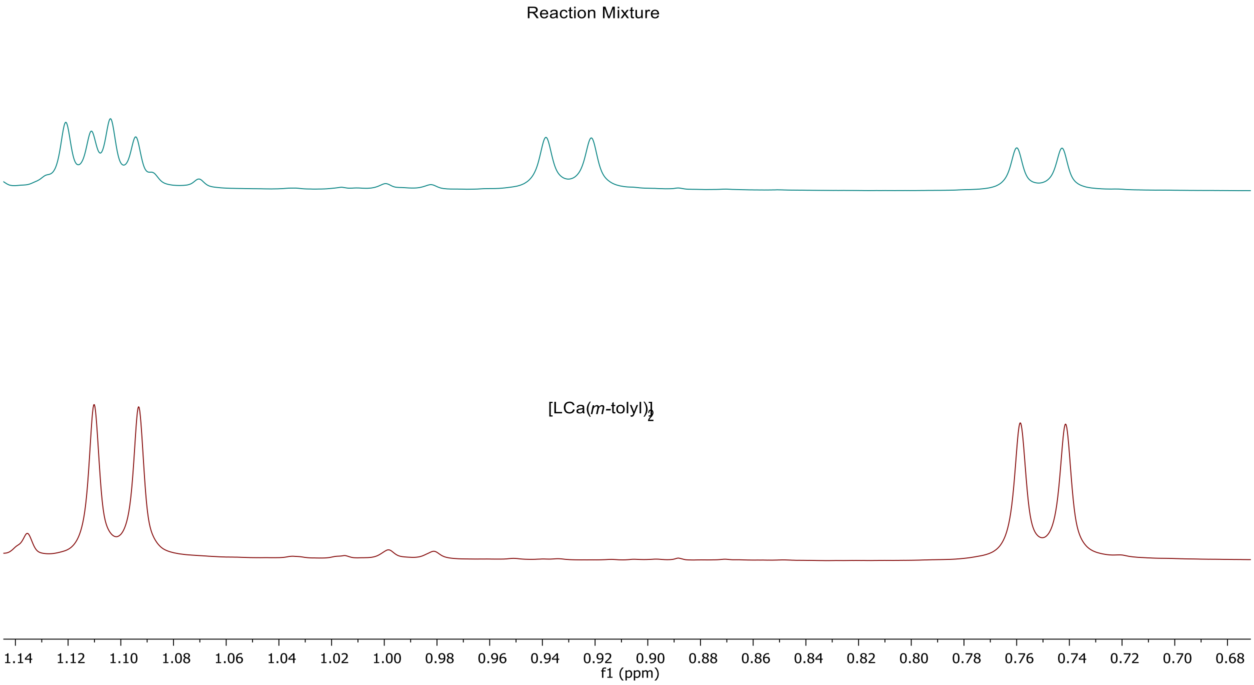

Figure S14*. ^{1}H NMR Spectrum (C_6D_6, 298 K, 400.13 MHz) of the reaction between $[LCaH]_2$ and 0.5 equiv. of m-tolyl$_2$Hg, zoomed in on the alkyl region demonstrating the presence of both $[(^{Dipp}BDI)Ca(H)m\text{-}tolylCa(^{Dipp}BDI)]$ (**6**) and $[(^{Dipp}BDI)Ca(m\text{-}tolyl)]_2$ (**7**).*

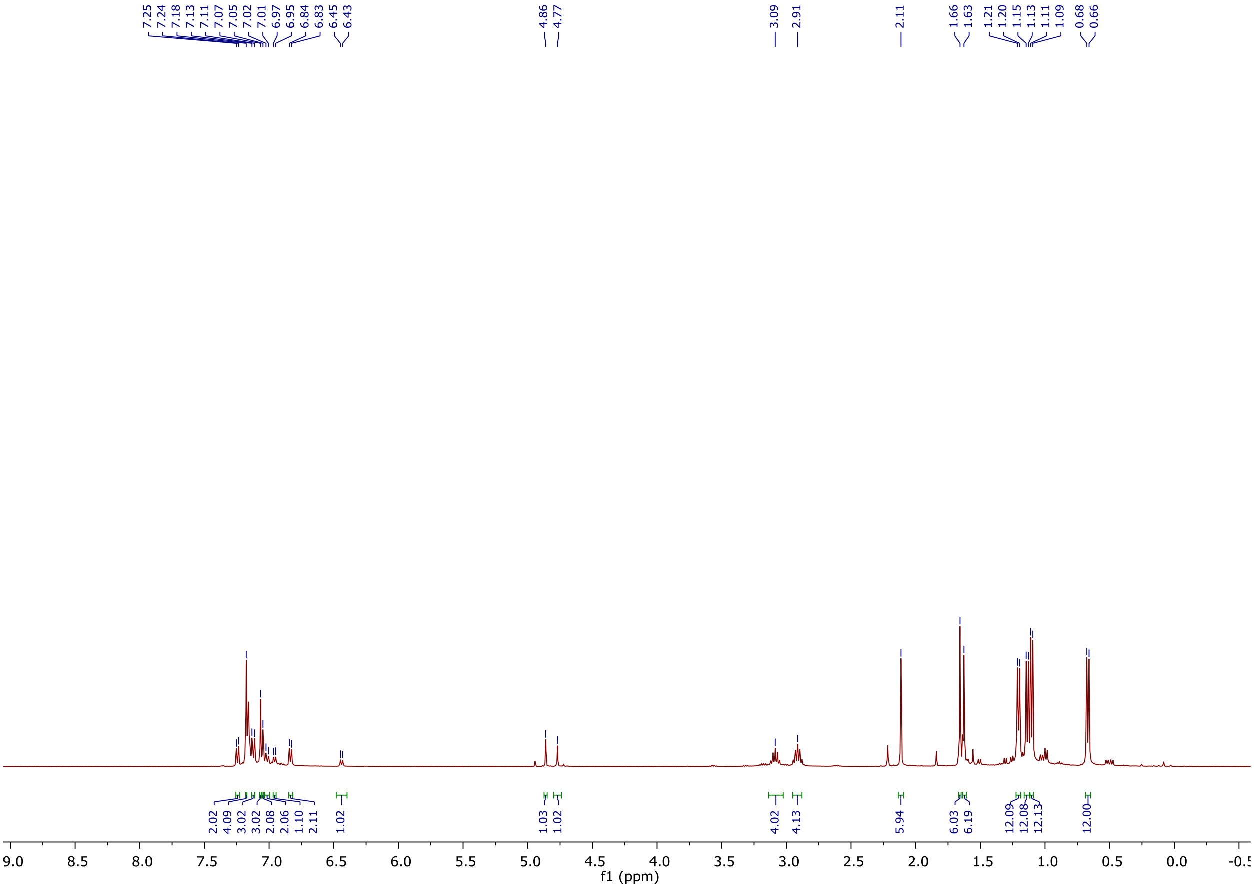

Figure S15. *^{1}H NMR Spectrum (C_6D_6, 298 K, 400.13 MHz) for [(^{Dipp}BDI)Ca(p-tolyl)]$_2$ (**8**).*

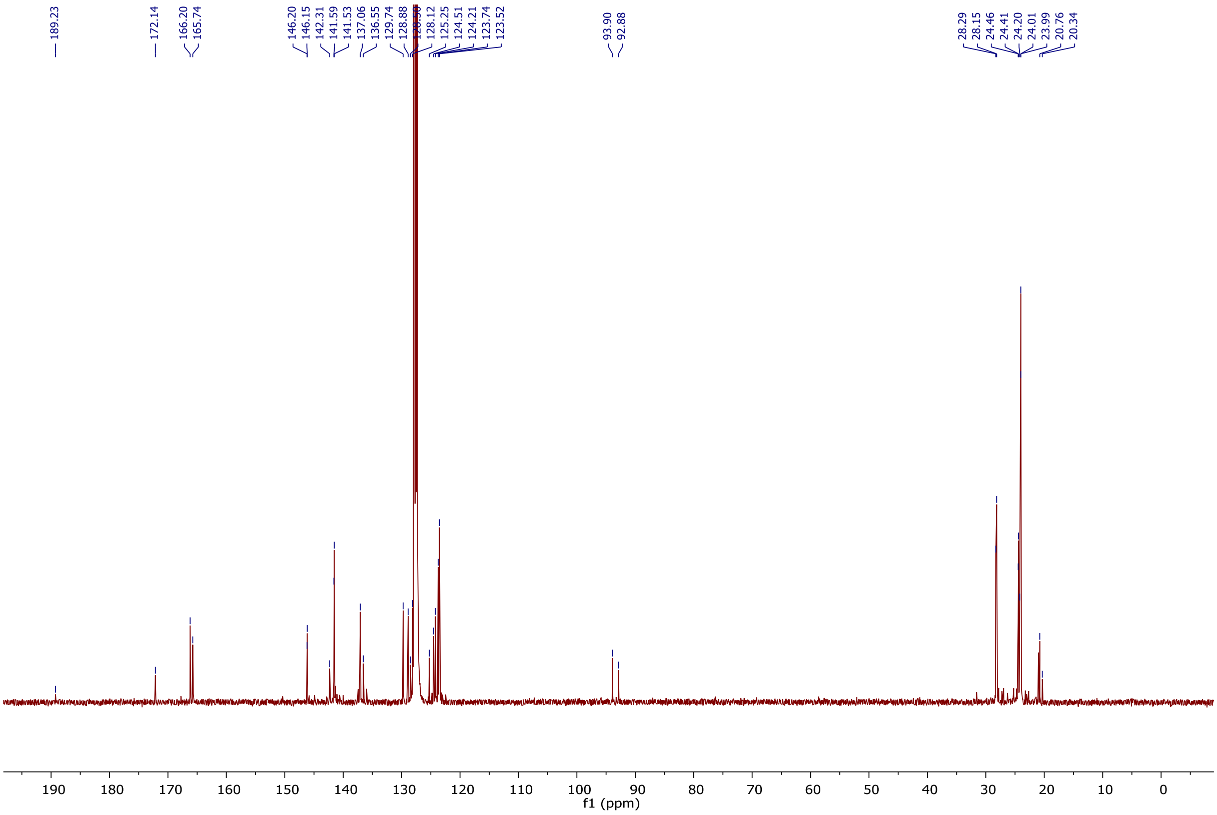

***Figure S16**. ^{13}C{^{1}H} NMR Spectrum (C_6D_6, 298 K, 100.62 MHz) for [(^{Dipp}BDI)Ca(p-tolyl)]$_2$ (**8**).*

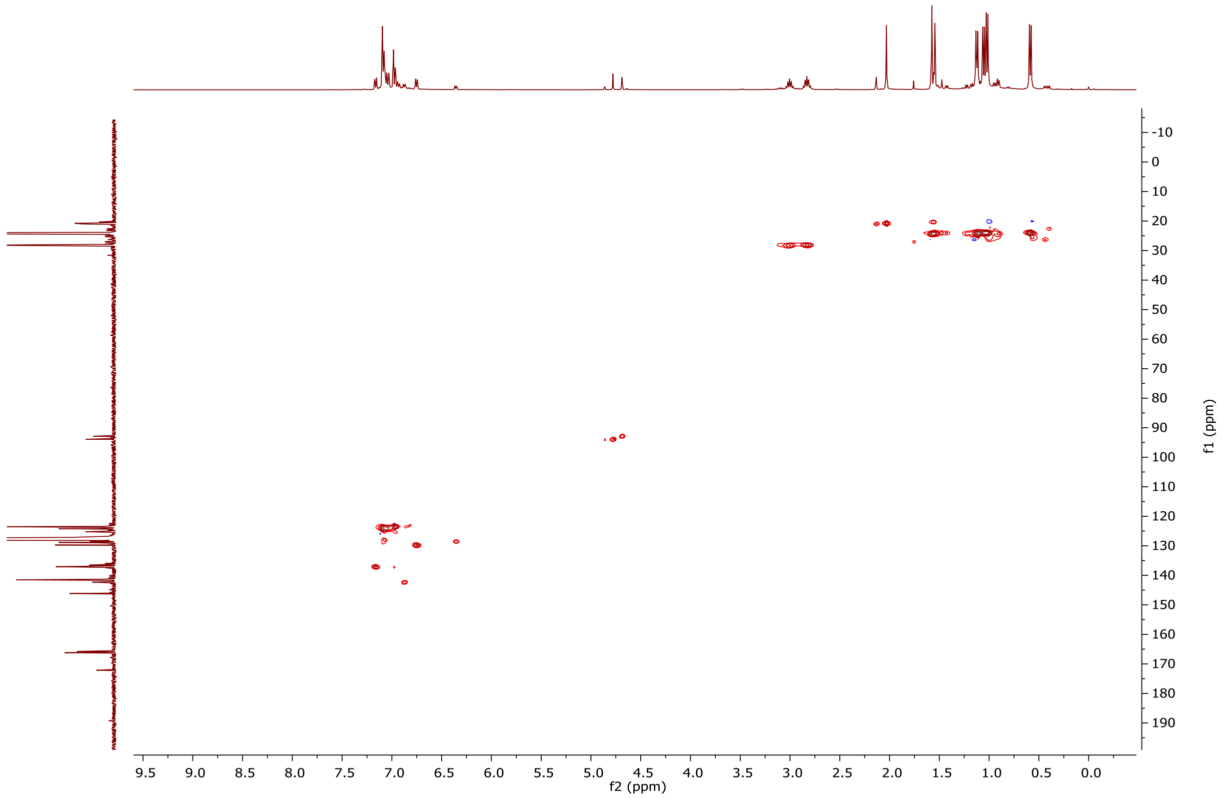

Figure S17. *^{1}H-^{13}C HSQC trace (C_6D_6, 298 K, 400.13, 100.62 MHz) for [(^{Dipp}BDI)Ca(p-tolyl)]$_2$ (**8**).*

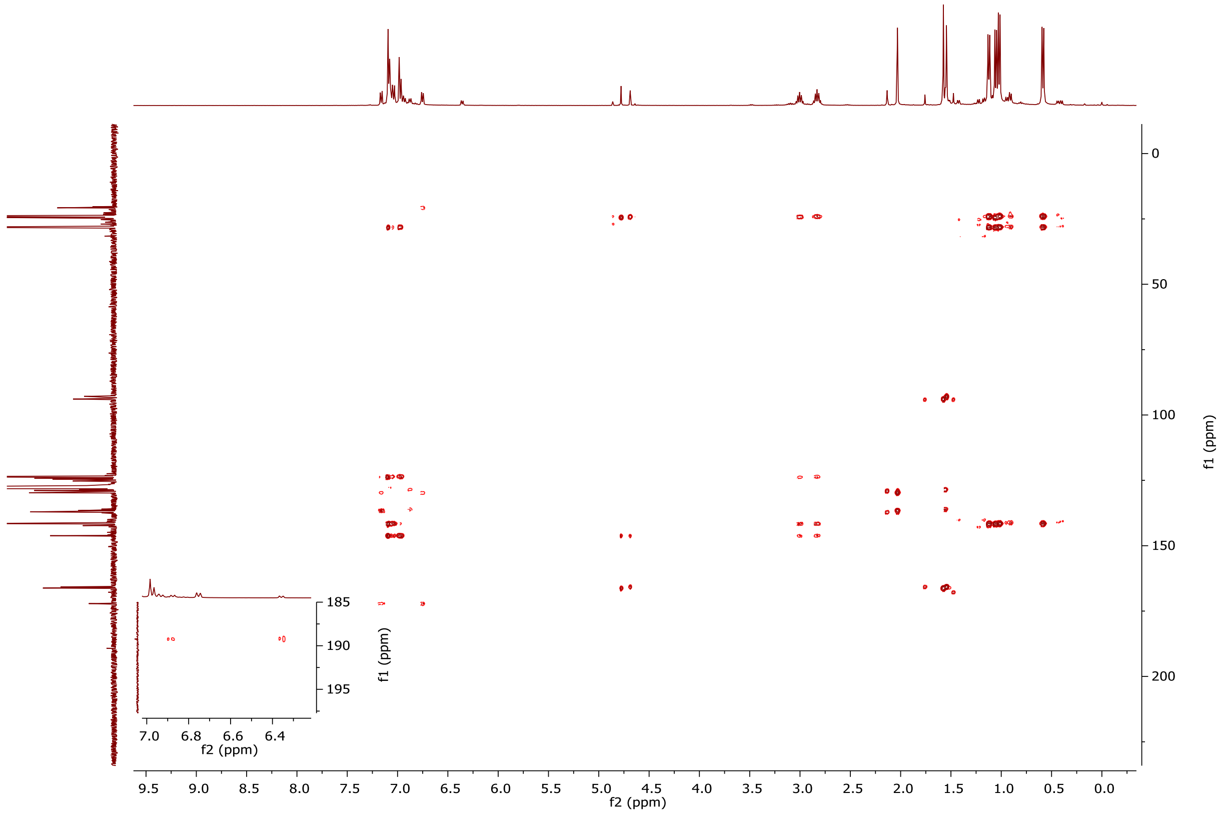

Figure S18. *^{1}H-^{13}C HMBC trace (C_6D_6, 298 K, 400.13, 100.62 MHz) for [(^{Dipp}BDI)Ca(p-tolyl)]$_2$ (**8**).*

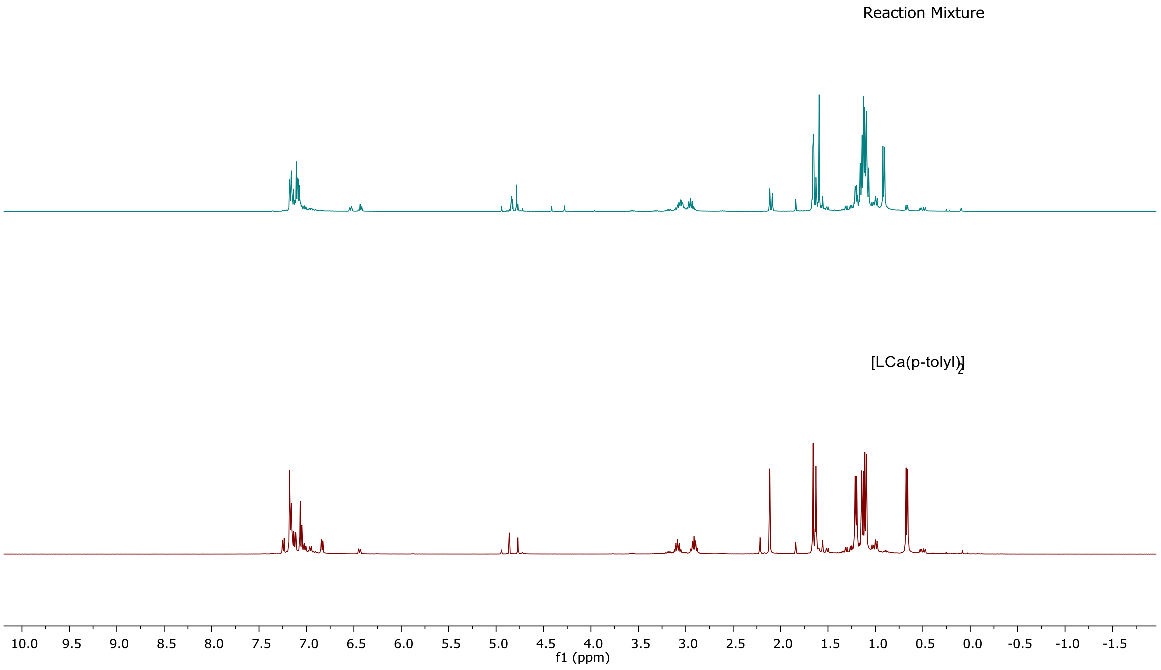

Figure S19. *^{1}H NMR Spectrum (C_6D_6, 298 K, 400.13 MHz) of the reaction between [LCaH]$_2$ and 0.5 equiv. of p-tolyl$_2$Hg.*

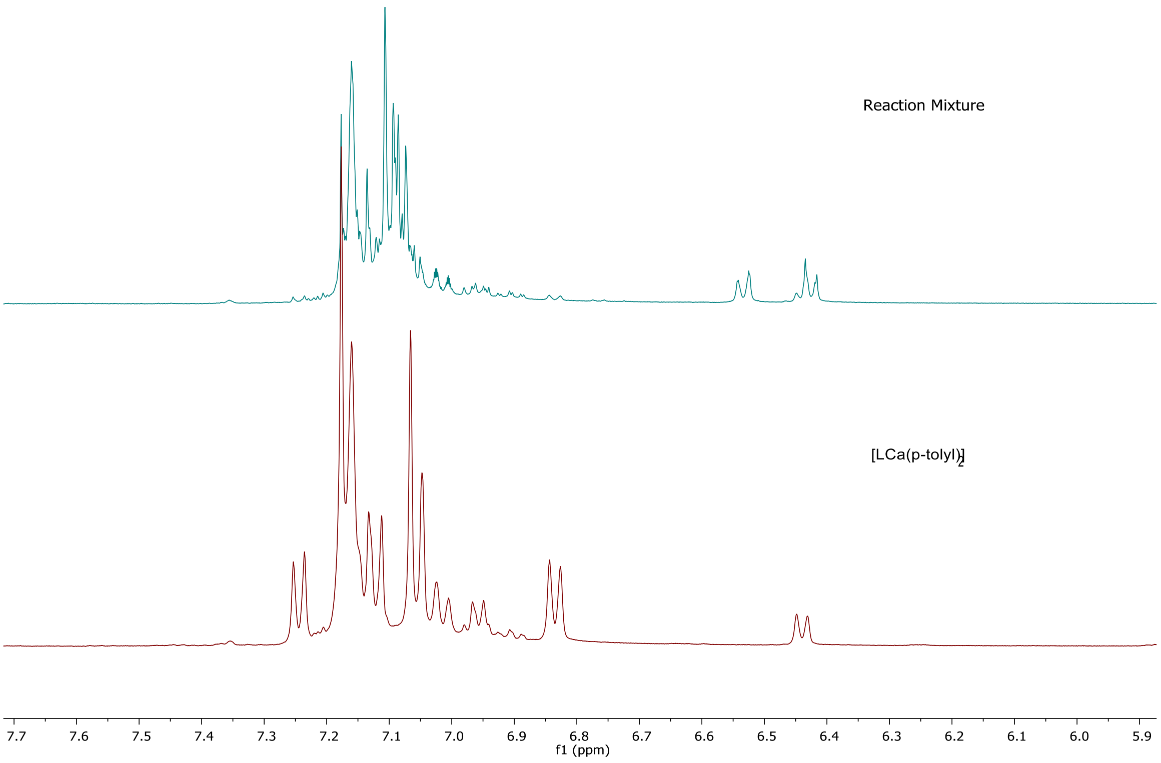

Figure S20. *^{1}H NMR Spectrum (C_6D_6, 298 K, 400.13 MHz) of the reaction between [LCaH]$_2$ and 0.5 equiv. of p-tolyl$_2$Hg, zoomed in on the aromatic region to demonstrate the presence of both [(^{Dipp}BDI)Ca(H)p-tolylCa(^{Dipp}BDI)] and [(^{Dipp}BDI)Ca(p-tolyl)]$_2$ (**8**)..*

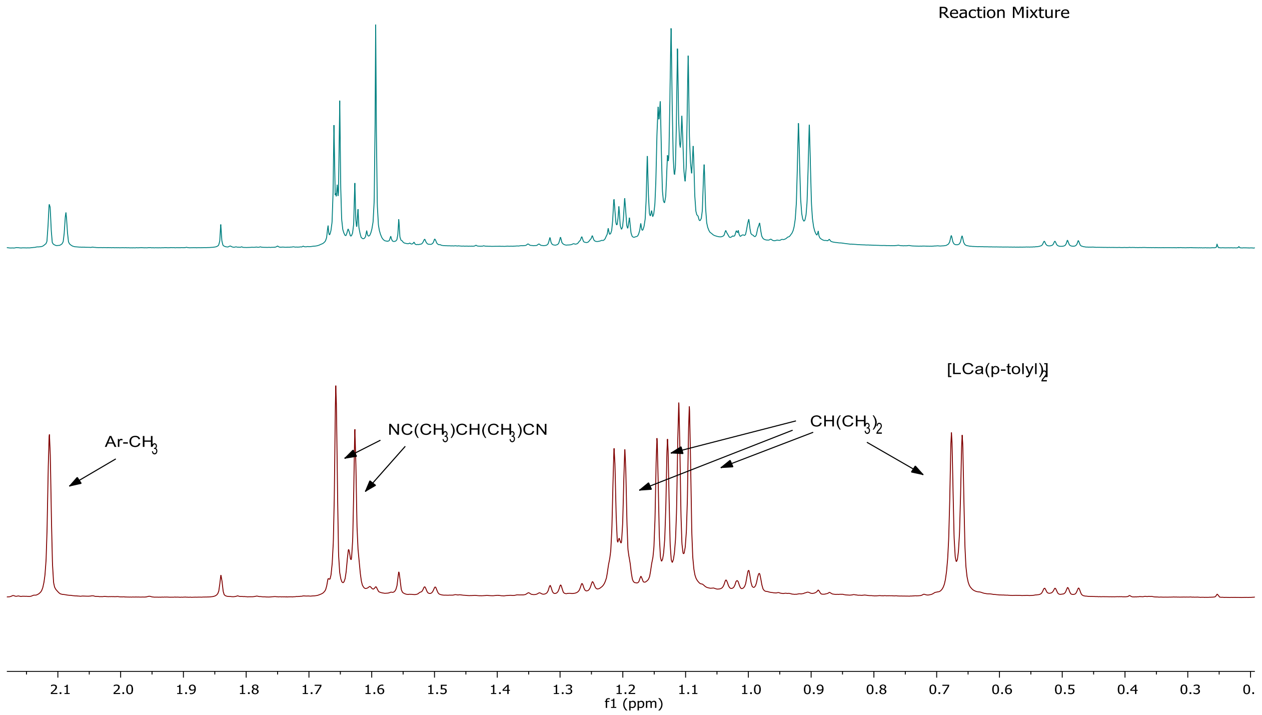

Figure S21. *^{1}H NMR Spectrum (C_6D_6, 298 K, 400.13 MHz) of the reaction between [LCaH]$_2$ and 0.5 equiv. of p-tolyl$_2$Hg, zoomed in on the alkyl region demonstrating the presence of both [(^{Dipp}BDI)Ca(H)p-tolylCa(^{Dipp}BDI)] and [(^{Dipp}BDI)Ca(p-tolyl)]$_2$ (**8**).*.

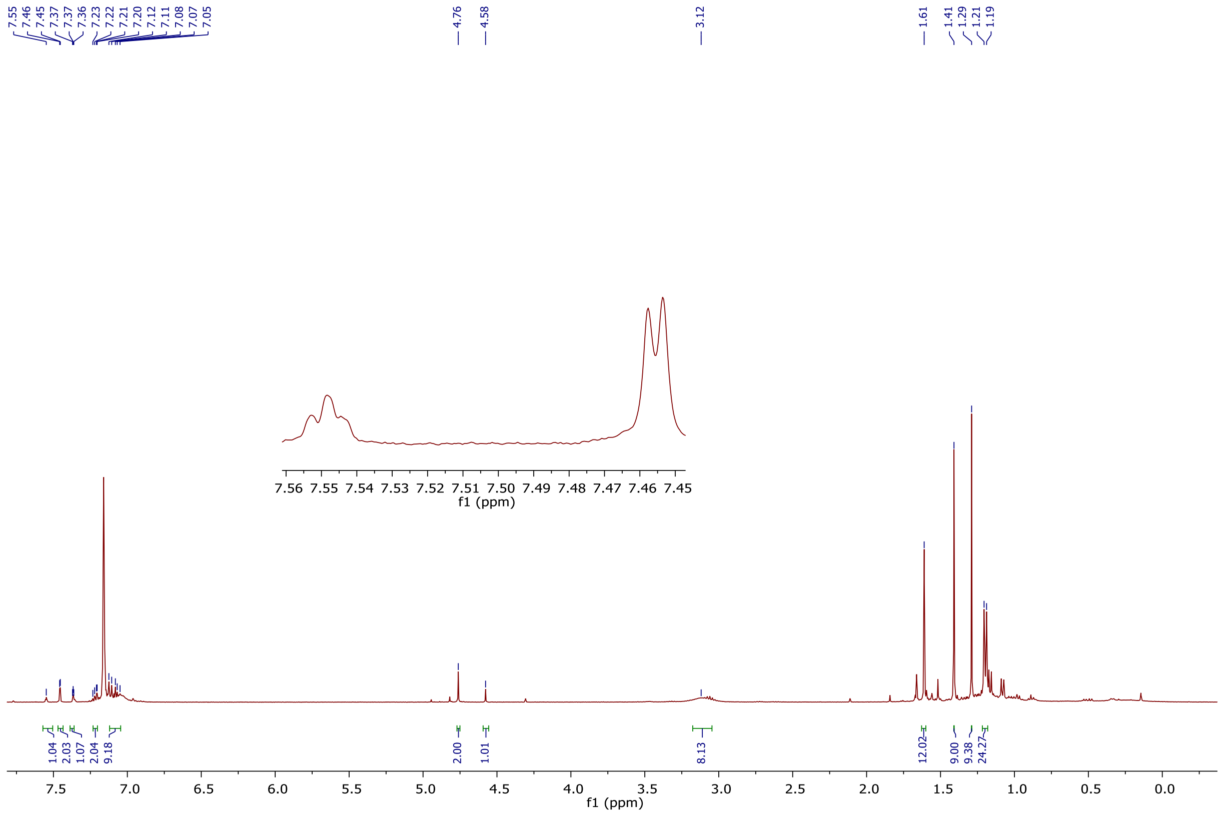

Figure S22. *^{1}H NMR Spectrum (C_6D_6, 298 K, 400.13 MHz) for [(^{Dipp}BDI)Ca(H)(3,5-^{t}Bu)$_2$PhCa(^{Dipp}BDI)] (**10**).*

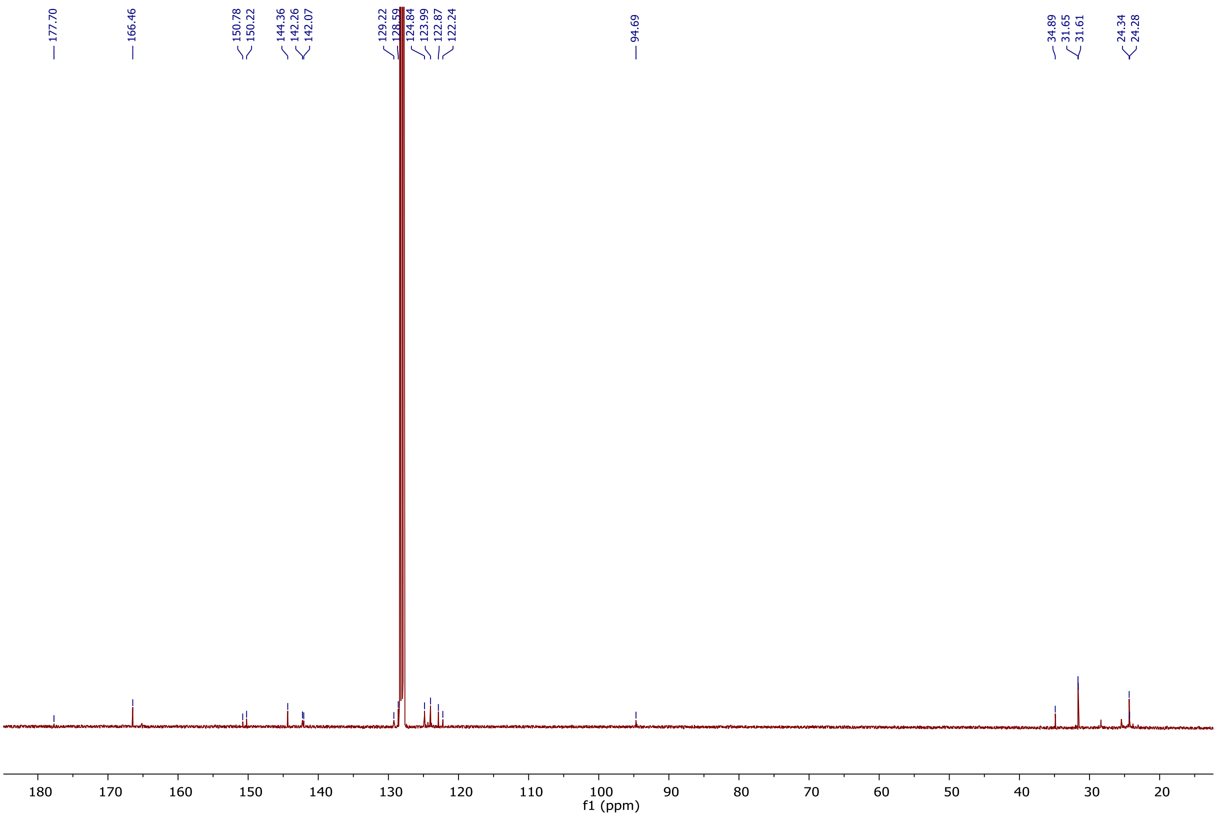

Figure S23. *$^{13}C\{^1H\}$ NMR Spectrum (C_6D_6, 298 K, 100.62 MHz) for [(^{Dipp}BDI)Ca(H)(3,5-^{t}Bu)$_2$PhCa(^{Dipp}BDI)]* (**10**).

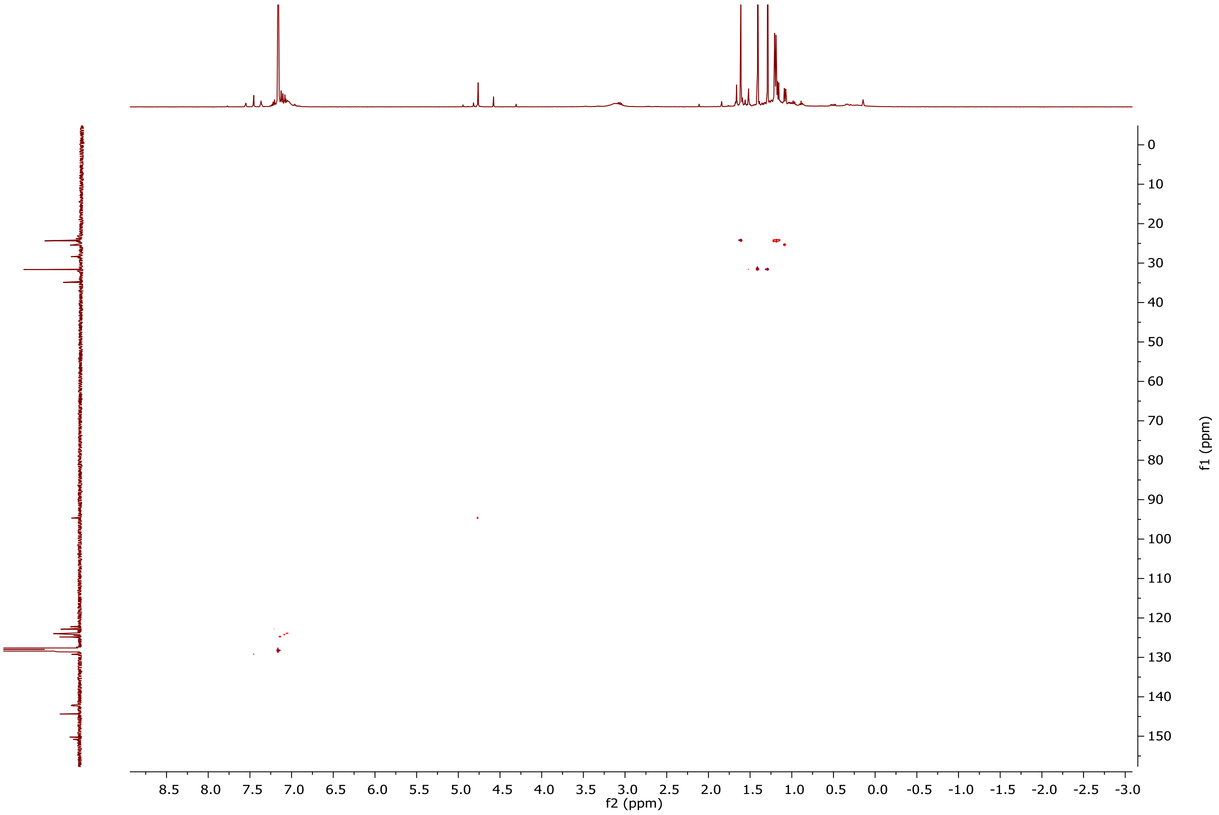

Figure S24. *^1H-^{13}C HSQC trace (C_6D_6, 298 K, 400.13, 100.62 MHz) for [(^{Dipp}BDI)Ca(H)(3,5-^{t}Bu)$_2$PhCa(^{Dipp}BDI)]* (**10**).

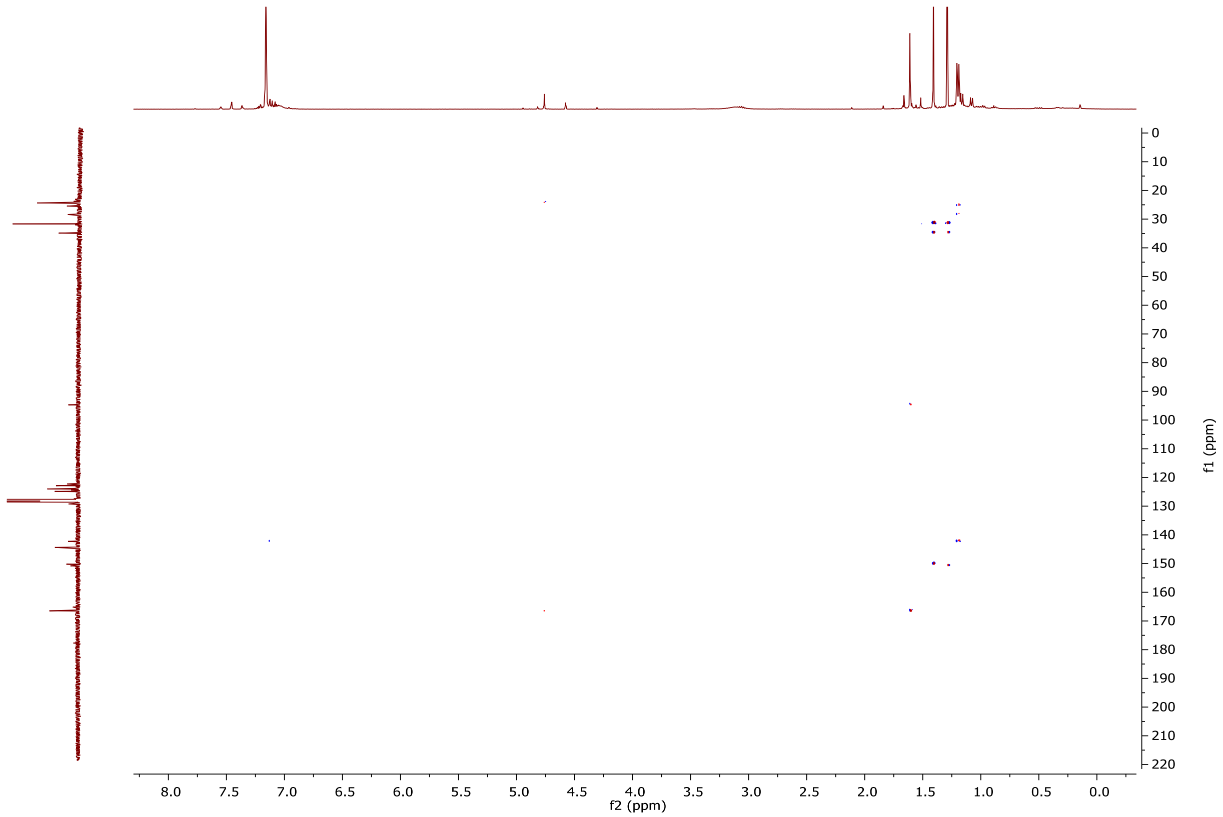

Figure S25. ^{1}H-^{13}C *HMBC trace (C_6D_6, 298 K, 400.13, 100.62 MHz) for [(^{Dipp}BDI)Ca(H)(3,5-^{t}Bu)$_2$PhCa(^{Dipp}BDI)] (**10**).*

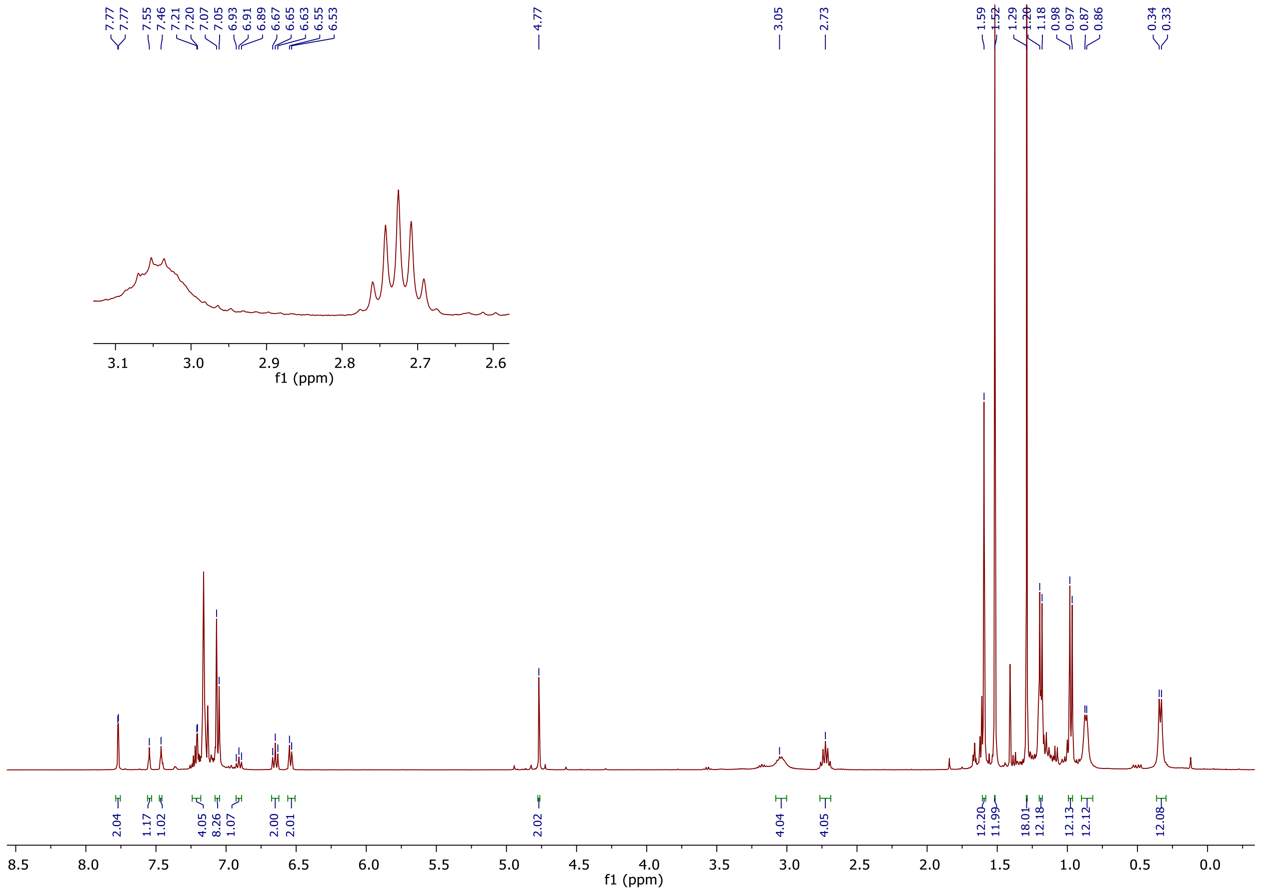

Figure S26. *^{1}H NMR Spectrum (C_6D_6, 298 K, 400.13 MHz) for [(^{Dipp}BDI Ca((3,5-tBu)$_2$Ph)PhCa(^{Dipp}BDI)] (**11**).*

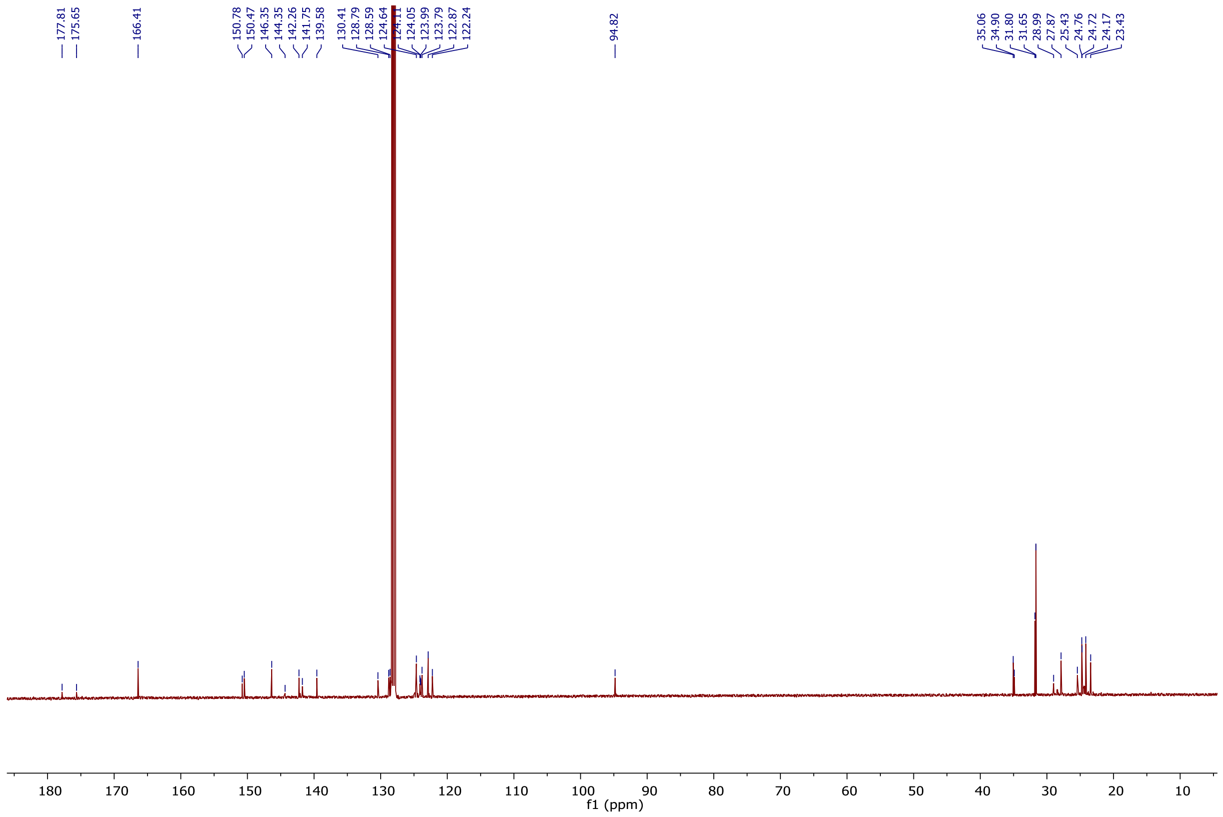

Figure S27. *$^{13}C\{^{1}H\}$ NMR Spectrum (C_6D_6, 298 K, 100.62 MHz) for [(^{Dipp}BDI Ca((3,5-tBu)$_2$Ph)PhCa(^{Dipp}BDI)] (**11**).*

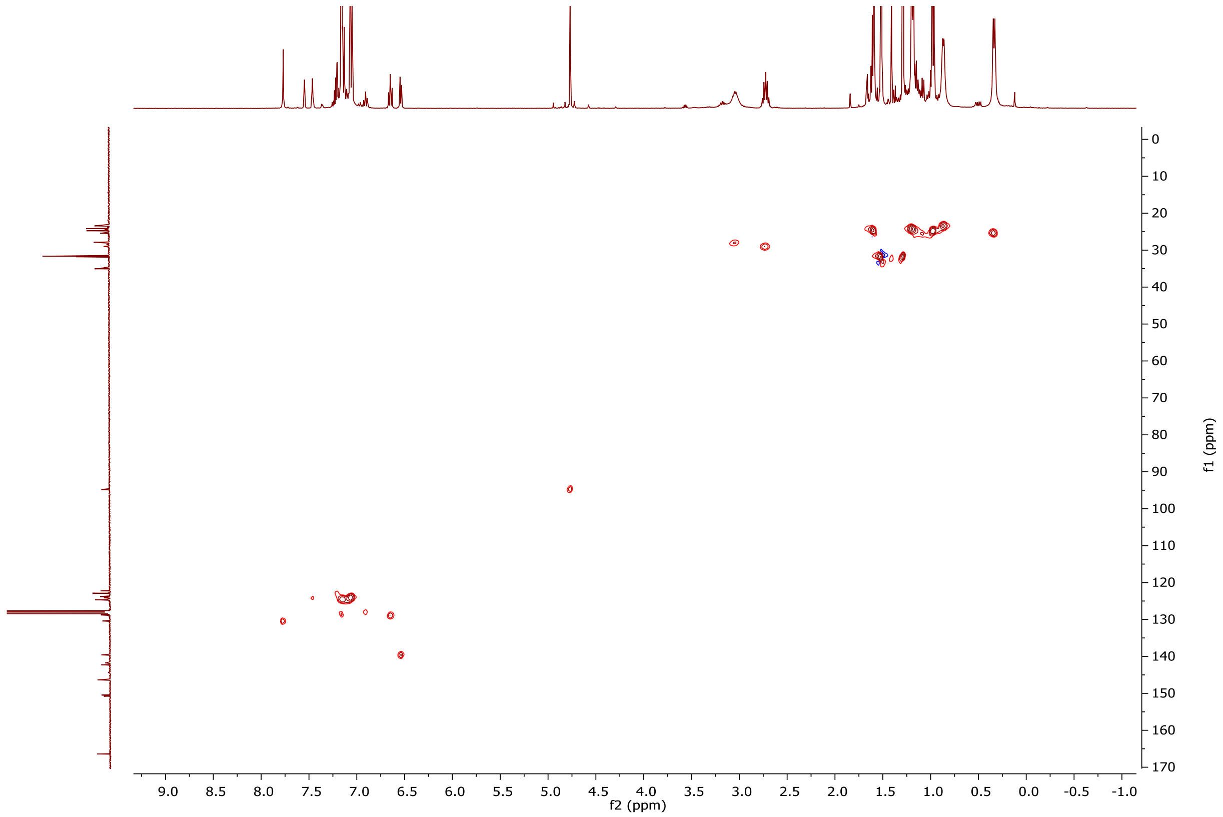

Figure S28. *^{1}H-^{13}C HSQC trace (C_6D_6, 298 K, 400.13, 100.62 MHz) for [(^{Dipp}BDI Ca((3,5-tBu)$_2$Ph)PhCa(^{Dipp}BDI)] (**11**).*

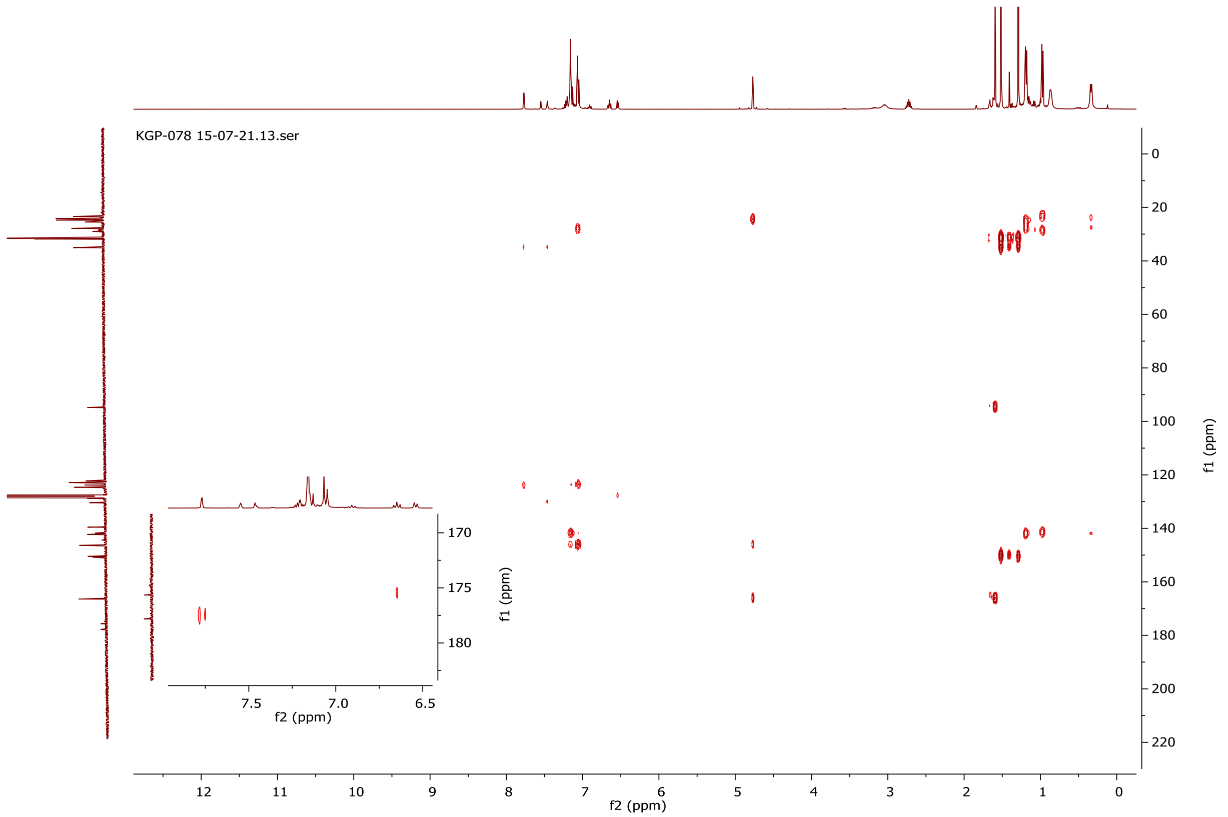

Figure S29. *^{1}H-^{13}C HMBC trace (C_6D_6, 298 K, 400.13, 100.62 MHz) for [(^{Dipp}BDI Ca((3,5-tBu)$_2$Ph)PhCa(^{Dipp}BDI)] (**11**).*

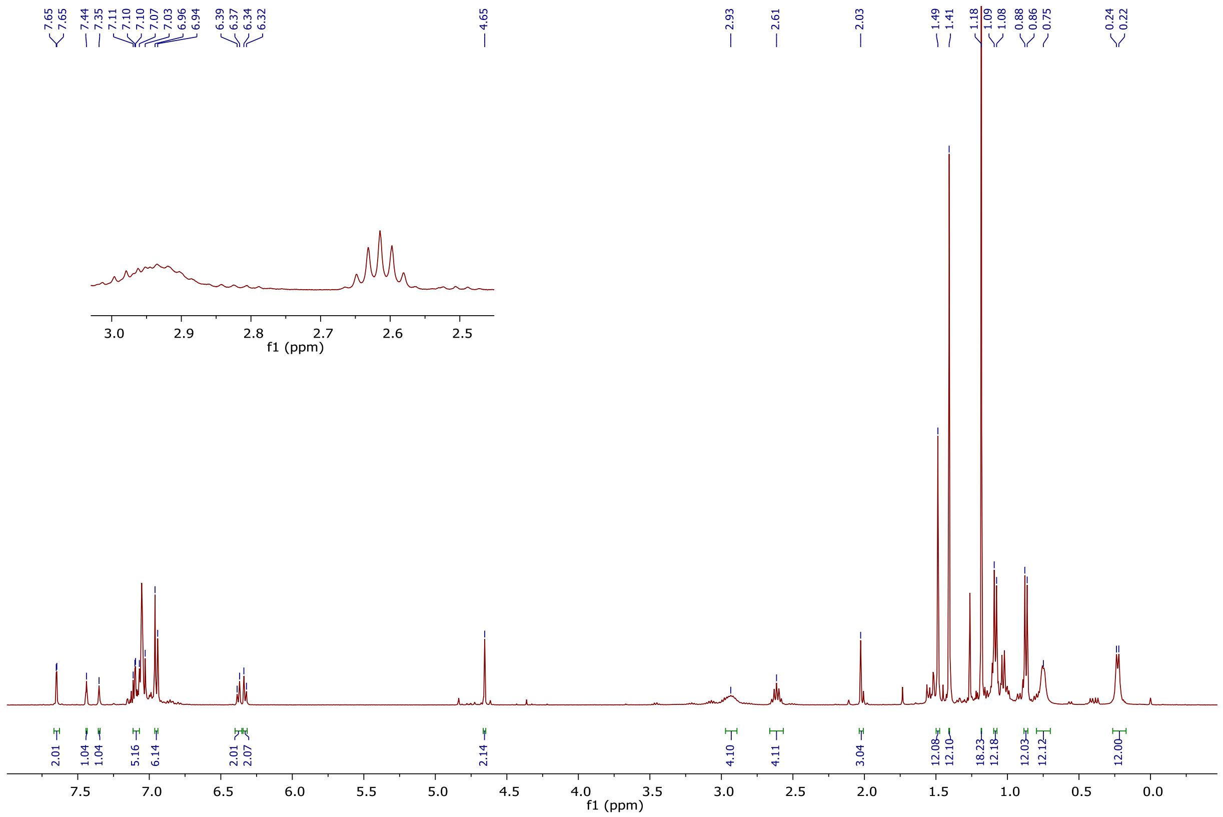

Figure S30*. ^{1}H NMR Spectrum (C_6D_6, 298 K, 400.13 MHz) for [(^{Dipp}BDI)LCa((3,5-tBu)$_2$Ph)p-tolylCa(^{Dipp}BDI)] (**12**)..*

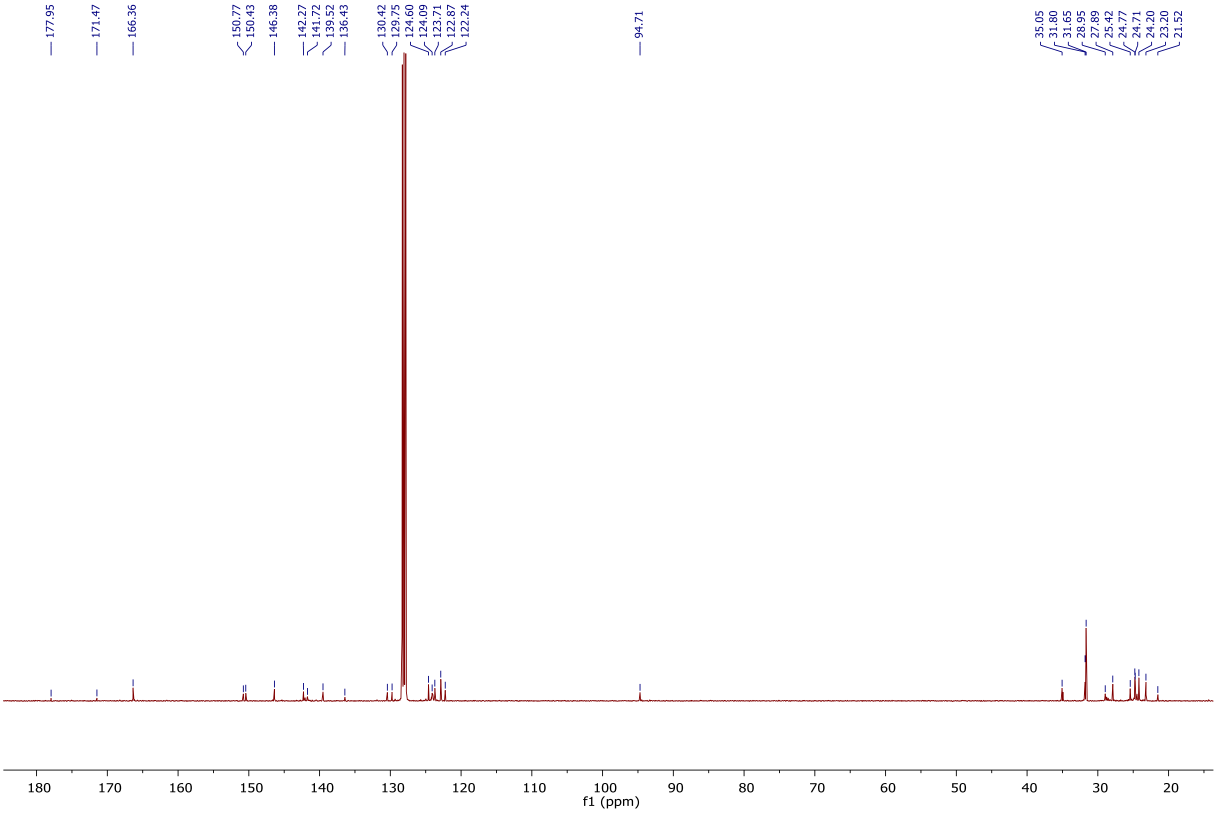

Figure S31. *$^{13}C\{^{1}H\}$ NMR Spectrum (C_6D_6, 298 K, 100.62 MHz) for [(^{Dipp}BDI)LCa((3,5-tBu)$_2$Ph)p-tolylCa(^{Dipp}BDI)] (**12**).*

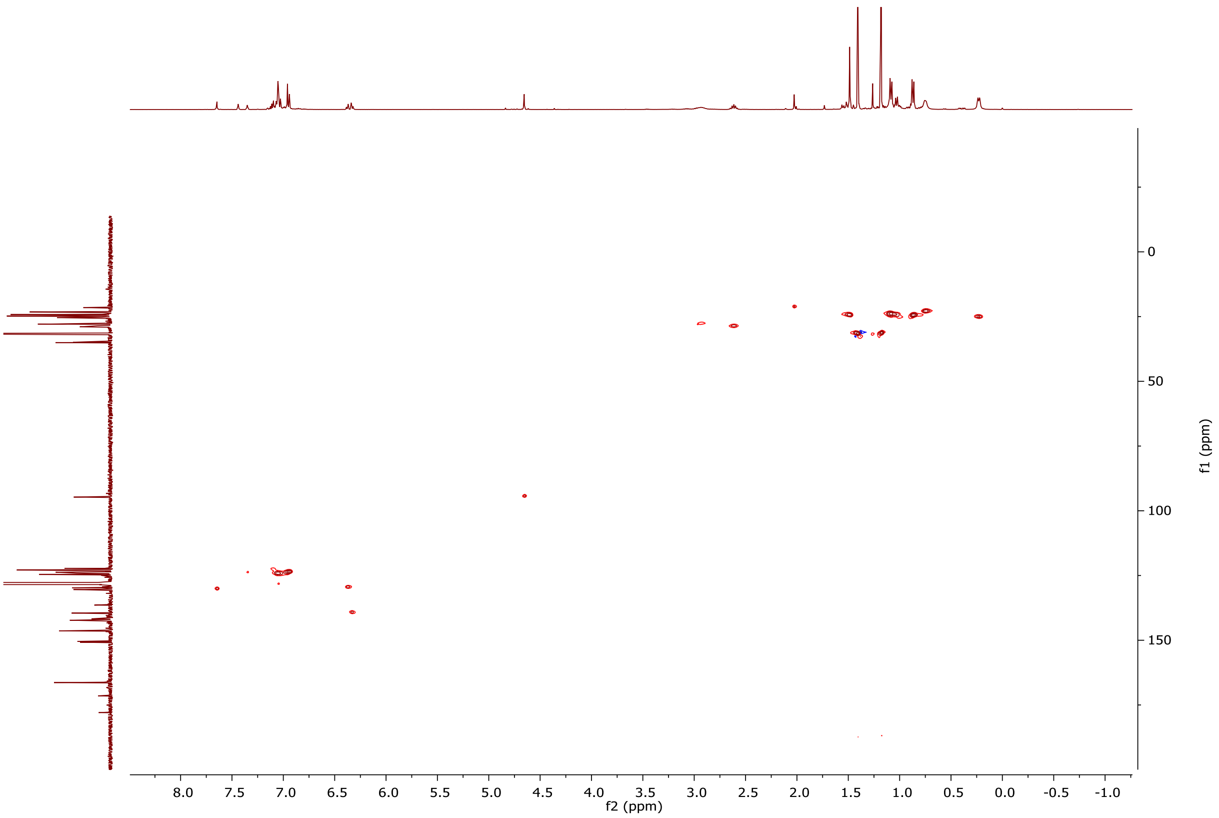

Figure S32. *^{1}H-^{13}C HSQC trace (C_6D_6, 298 K, 400.13, 100.62 MHz) for [(^{Dipp}BDI)LCa((3,5-tBu)$_2$Ph)p-tolylCa(^{Dipp}BDI)] (**12**).*

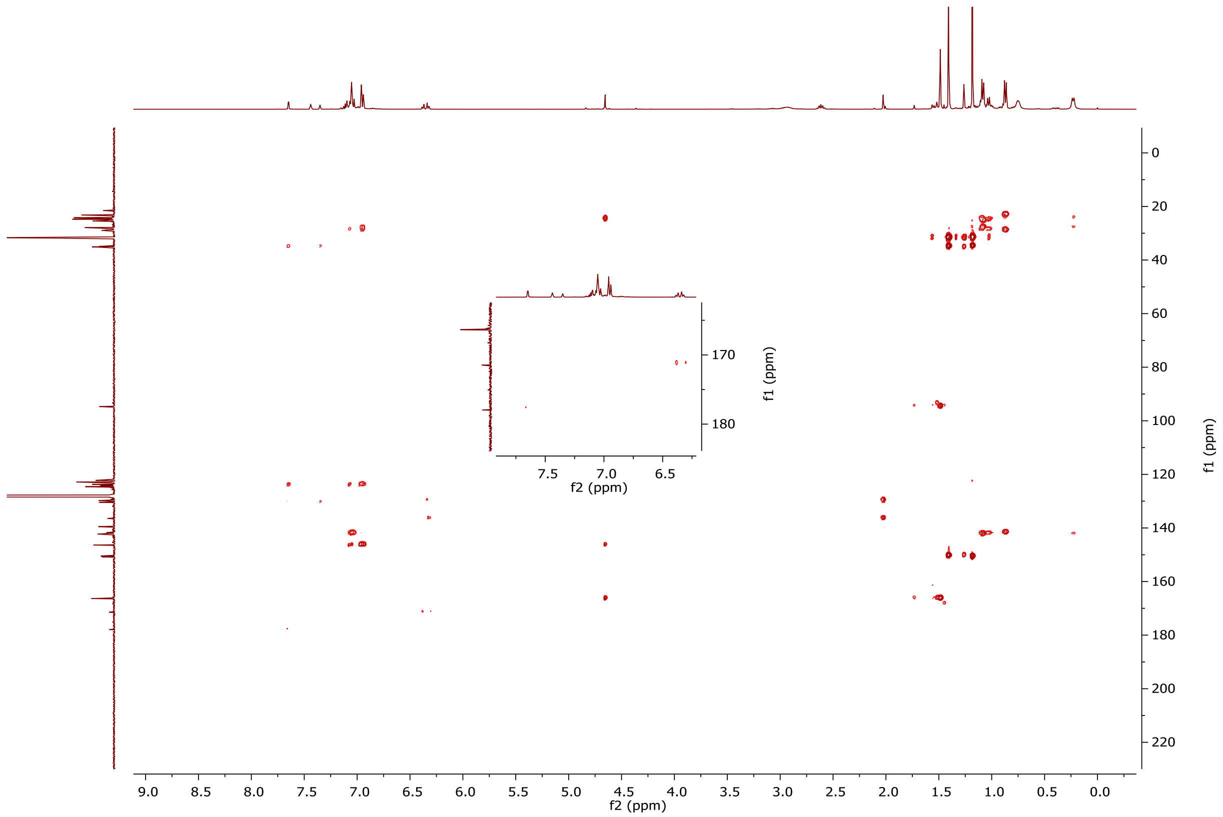

Figure S33. *^{1}H-^{13}C HMBC trace (C_6D_6, 298 K, 400.13, 100.62 MHz) for [([Dipp]BDI)LCa((3,5-tBu)$_2$Ph)p-tolylCa([Dipp]BDI)]* ***(12)****.*

Single Crystal X-ray Analysis

Data for **4**, **5**, **7** and **10** were collected on an Agilent Xcalibur diffractometer (using Mo-Kα radiation) while those for **8**, **9**, **11**, **12** and **14** were obtained using an Agilent SuperNova instrument and a Cu-Kα source. All experiments were conducted at 150 K, solved using SHELXT[1] and refined using SHELXL[2] via the Olex2[3] interface. Refinements were unremarkable except for the points outlined hereafter. Distance and ADP restraints were employed, on merit, in all disordered regions to assist convergence.

The asymmetric unit in the structure of **4** contains one molecule of the calcium dimer and a region of solvent. The latter evidently corresponded to one molecule of pentane, but it was very disordered. This is not unexpected given that the solvent resides in channels that are parallel to the *a*-axis, in which molecules randomly orient themselves. Thus, the solvent in the structure was addressed using the masking algorithm available in Olex2, with allowance made for same in the formula as presented. H1 was located and refined without restraints. The only other limitation with this crystal was the weakness of the diffraction. That said, the model is entirely unambiguous.

In the structure of **5**, the asymmetric unit was noted to contain one molecule of the calcium dimer complex and one molecule of hexane. The hydride ligand was located and refined freely. The hydrogen atoms attached to C59 were also located and these were refined subject to being 0.98 Å from the parent carbon atom. 55:45 disorder was modelled for the methyl groups based on C64 and C65, and the solvent moiety was modelled as two components in a 50:50 ratio.

The asymmetric unit in the structure of **7** contains half of a dimer and one molecule of benzene. The hydrogen atoms attached to C31 and C35 were located and refined freely. 75:25 disorder was modelled for the solvent, with both components therein being treated as rigid hexagons.

The asymmetric unit in the structure of **8** comprises half of a molecule, in which C30, C34, C35, and to C39 are coincident with a crystallographic 2-fold rotation axis that serves to generate the remainder of the dimer. The symmetry of the molecule has resulted in the hydrogen atoms pertaining to the methyl moieties based on C34 and C39 being disordered over two positions. The crystal moved radically during the data collection and, hence, collected data were credible to a maximum resolution of 0.79 Å.

One molecule of the calcium containing dimer plus a molecule of toluene constitute the asymmetric unit in **9**. Despite copious crystallisation efforts, the crystal quality could not be augmented relative to the sample used in this experiment for which the raw data exhibited some smearing of the electron density above and beyond the 20% twinning that was accounted for during integration. C63-C65 were treated for 40:60 disorder. H1, H24 and H35 were located and refined freely, while all three hydrogens attached C12 were located and refined at a distance of 0.98 Å from the parent atom. The solvent moiety also revealed 60:40 disorder and the phenyl rings therein were refined as rigid hexagons.

In **10**, there is one molecule of the calcium-based dimer plus one and a half molecules of benzene. The complete solvent molecule was modelled to take account of 65:35 disorder, while the half molecule lies close to a crystallographic inversion centre which serves to generate the remainder. The hydride was located and refined without restraints.

The asymmetric unit in **11** is host to 3 disordered molecules of benzene (50:00, 50:50 and 55:45 occupancy fractions, respectively) as well as one molecule of the calcium complex.
Solvent components were treated as regular hexagons.

The asymmetric unit in the structure of **12**, comprises one dimer molecule and some solvent. Disorder in the main feature is confined to splitting of C37-39 in a 55:45 ratio, over two sites, and this was readily modelled. However, the guest solvent was disordered to a degree whereby modelling with not have justified the associated parameterisation. As such, the associated electron-density was, treated with the solvent mask algorithm in Olex-2. An allowance has been made in the formula, as presented, for three molecules of benzene per unit cell.

One molecule of benzene accompanies half of a calcium-based tetramer, in the structure of **14**. The methyl groups in the isopropyl functionality based on C59 were treated for 57:43 disorder, and the whole of the benzene moiety treated for 50:50 disorder.
Despite copious efforts, the size and quality of the crystals from which the sample arose could not be augmented. Hence, the crystal employed in the crystallographic experiment was smaller on one dimension than desirable, and this impacted on the diffraction intensities. Moreover, there was evidence in the raw data of minor domains being present, but integration of same (followed by refinement) did not lead to any significant improvement of the model or residuals. Ultimately, therefore, the raw data were integrated as arising from a single crystal, as the test

refinement (based on three domains) showed that the associated batch scale factor for the major component was in the region of 95%. The background mask was reduced, slightly, during data reduction, to minimise contamination of the reflection intensities by the minor domains.

Crystallographic data for all compounds have been deposited with the Cambridge Crystallographic Data Centre as supplementary publications CCDC 2260391-2260399 for **4, 5, 7, 8, 9, 10, 11, 12** and **14**, respectively. Copies of these data can be obtained free of charge on application to CCDC, 12 Union Road, Cambridge CB2 1EZ, UK [fax(+44) 1223 336033, e-mail: deposit@ccdc.cam.ac.uk.

Table S1: Crystal data and structure refinement for compounds **4**, **5, 7** and **8**.

Identification code	**e21msh10 (4)**	**e21msh08 (5)**	**e22msh05 (7)**	**s22msh16 (8)**
Empirical formula	$C_{70}H_{102}Ca_2N_4$	$C_{71}H_{104}Ca_2N_4$	$C_{84}H_{108}Ca_2N_4$	$C_{36}H_{48}CaN_2$
Formula weight	1079.71	1093.74	1253.90	548.84
Crystal system	monoclinic	monoclinic	triclinic	tetragonal
Space group	$P2_1/n$	$P2_1/n$	P–1	$P4_32_12$
a/ Å	12.4131(4)	12.0271(3)	11.1859(5)	14.31188(14)
b/ Å	41.3998(10)	28.4491(7)	13.2501(5)	14.31188(14)
c/ Å	13.7992(5)	19.9507(5)	14.0992(6)	31.9348(4)
α/ °	90	90	102.251(3)	90
β/ °	104.632(4)	98.561(3)	110.173(4)	90
γ/ °	90	90	99.588(3)	90
U/ Å^3	6861.4(4)	6750.3(3)	1850.22(14)	6541.20(15)
Z	4	4	1	8
ρ_{calc}/ g cm^{-3}	1.045	1.076	1.125	1.115
μ/ mm^{-1}	0.206	0.210	0.200	1.824
F(000)	2360.0	2392.0	680.0	2384.0
Crystal size/ mm^3	0.28 × 0.174 × 0.156	0.557 × 0.367 × 0.266	0.527 × 0.341 × 0.218	0.346 × 0.191 × 0.092
2θ range for data collection/°	5.896 to 54.966	5.92 to 60.62	5.864 to 60.64	6.768 to 153.24
Index ranges	$-14 \le h \le 16$, $-52 \le k \le 53$, $-15 \le l \le 17$	$-16 \le h \le 16$, $-40 \le k \le 40$, $-28 \le l \le 21$	$-14 \le h \le 15$, $-16 \le k \le 18$, $-18 \le l \le 14$	$-15 \le h \le 17$, $-17 \le k \le 17$, $-39 \le l \le 24$
Reflections collected	32083	67293	17155	27495
Independent reflections, R_{int}	14869, 0.0575	18018, 0.0288	9421, 0.0227	6340, 0.0340
Data/restraints/parameters	14869/0/669	18018/243/804	9421/192/455	6340/0/369
Goodness–of–fit on F^2	1.002	1.017	1.053	1.009
Final R1, wR2 [I>=2σ(I)]	0.0683, 0.1395	0.0467, 0.1101	0.0454, 0.1096	0.0408, 0.1006
Final R1, wR2 [all data]	0.1221, 0.1723	0.0717, 0.1231	0.0615, 0.1200	0.0525, 0.1115
Largest diff. peak/hole/ e Å^{-3}	0.34/–0.33	0.56/–0.28	0.35/–0.36	0.20/–0.26
Flack Parameter	–	–	–	–0.003(4)

Table S2: Crystal data and structure refinement for compounds **9**, **10**, **11**, **12** and **14**.

Identification code	**s22msh81 (9)**	**e21msh25 (10)**	**s21msh59 (11)**	**s21msh68 (12)**	**s22msh86 (14)**
Empirical formula	$C_{72}H_{98}Ca_2N_4$	$C_{81}H_{113}Ca_2N_4$	$C_{96}H_{126}Ca_2N_4$	$C_{88}H_{119}Ca_2N_4$	$C_{140}H_{186}Ca_4Cl_2N_8$
Formula weight	1099.70	1222.91	1416.16	1313.02	2212.18
Crystal system	triclinic	monoclinic	triclinic	triclinic	monoclinic
Space group	*P*–1	$P2_1$/n	*P*–1	*P*–1	$P2_1/c$
a/ Å	12.1327(6)	14.7119(1)	13.2053(1)	12.7553(2)	16.0402(5)
b/ Å	14.7627(9)	22.1897(2)	13.7705(2)	13.2827(2)	16.2877(6)
c/ Å	19.3476(7)	23.3651(3)	23.6922(3)	26.9923(5)	24.6017(7)
α/ °	105.414(5)	90	95.630(1)	80.009(2)	90
β/ °	90.169(4)	99.1060(10)	91.556(1)	81.863(2)	95.011(3)
γ/ °	93.676(5)	90	93.176(1)	64.624(2)	90
U/ $Å^3$	3333.1(3)	7531.47(13)	4278.60(9)	4057.65(13)	6402.8(4)
Z	2	4	2	2	2
ρ_{calc}/ g cm^{-3}	1.096	1.079	1.099	1.075	1.147
μ/ mm^{-1}	1.790	0.194	1.496	1.542	2.242
F(000)	1196.0	2668.0	1540.0	1430.0	2392.0
Crystal size/ mm^3	0.384 × 0.113 × 0.035	0.668 × 0.403 × 0.27	0.422 × 0.315 × 0.186	0.223 × 0.136 × 0.103	0.129 × 0.117 × 0.036
2θ range for data collection/°	6.744 to 144.256	5.882 to 60.804	6.708 to 147.04	7.426 to 146.076	6.516 to 147.206
Index ranges	–14 ≤ h ≤ 10, –17 ≤ k ≤ 18, –23 ≤ l ≤ 23	–19 ≤ h ≤ 20, –31 ≤ k ≤ 29, –33 ≤ l ≤ 31	–16 ≤ h ≤ 14, –17 ≤ k ≤ 17, –29 ≤ l ≤ 29	–15 ≤ h ≤ 15, –16 ≤ k ≤ 10, –33 ≤ l ≤ 33	–19 ≤ h ≤ 14, –20 ≤ k ≤ 19, –30 ≤ l ≤ 27
Reflections collected	13018	82759	60426	60529	52565
Independent reflections, R_{int}	13018, 0.0761*	20379, 0.0248	17101, 0.0211	16100, 0.0242	12759, 0.0780
Data/restraints/parameters	13018/300/820	20379/0/856	17101/546/1052	16100/0/835	12759/175/764
Goodness–of–fit on F^2	0.782	1.030	1.027	1.045	1.021
Final *R*1, *wR*2 [*I*>=2σ(*I*)]	0.0599, 0.1318	0.0443, 0.1103	0.0375, 0.1018	0.0347, 0.0937	0.0767, 0.2009
Final *R*1, *wR*2 [all data]	0.1439, 0.1518	0.0631, 0.1210	0.0385, 0.1028	0.0374, 0.0961	0.1102, 0.2322
Largest diff. peak/hole/ e $Å^{-3}$	0.40/–0.42	0.33/–0.27	0.26/–0.31	0.27/–0.20	0.60/–0.68

*R_{int} pertaining to HKLF5 file (based on comparison of scale factors arising from the component analysis for each domain and overlapping reflections which contribute to each twinned component).

Computational Details

Calculations were carried out using the Gaussian09 package[7] at the DFT level by means of the hybrid density functional B3PW91.[8] A triple-zeta 6-311G basis set augmented by a polarization and diffuse function was used for the Ca atom. Polarized all electron triple-zeta 6-311G(d,p) basis set were used for the N and O atoms whereas a polarized all electron double-zeta 6-31G(d,p) basis set were used for the C and H atoms. Vibrational frequencies were systematically computed in order to characterize the nature of the stationary points. The ground states and transition states, in particular, have been computed as optimized stationary points displaying, respectively, no imaginary frequency and only one negative imaginary frequency, as verified by means of analytical frequency calculations at 298.15 K and 1 atm. The geometry optimizations have been achieved without any geometrical constraints and IRC calculations were carried out in order to confirm the connectivity between reactant(s), transition state and product(s). The electron density and partial charge distribution were examined in terms of localized electron-pair bonding units using the NBO program.[9] Through this method, the input atomic orbital basis set is transformed via natural atomic orbitals (NAOs) and natural hybrid orbitals (NHOs) into natural bond orbitals (NBOs), which correspond to the localised one centre ("lone pair") and two-centre ("bond") elements of the Lewis structure. All the possible interactions between "filled" (donor) Lewis-type NBOs and "empty" (acceptor) non-Lewis NBOs orbitals, together with their energetic quantification (stabilization energy), have been obtained by a second-order perturbation theory analysis of the Fock matrix. The effect of dispersion forces was taken into account by using the-D3 Grimme's correction with the Becke-Johnson damping.[10] In all calculations, the solvent effect was taken into account through the SMD continuum solvation model[11] by using toluene as solvent as experimentally.

Figure S34:

The optimized structure of **4** and **5** (**4_{opt}** and **5_{opt}** respectively), with the most significant bond lengths (blue) and NPA charges (red).

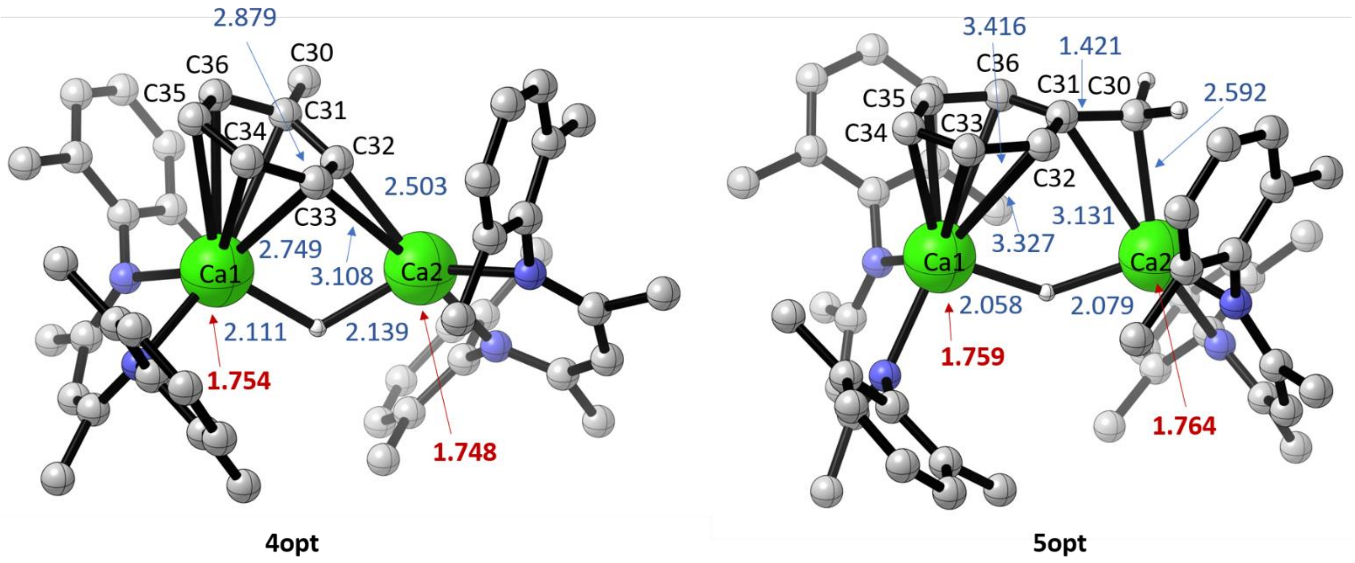

Figure S35:

The optimized structure of **TS2**, **INT2** and **TS3**, **INT3** with their most significant bond lengths (blue) and NPA charges (red).

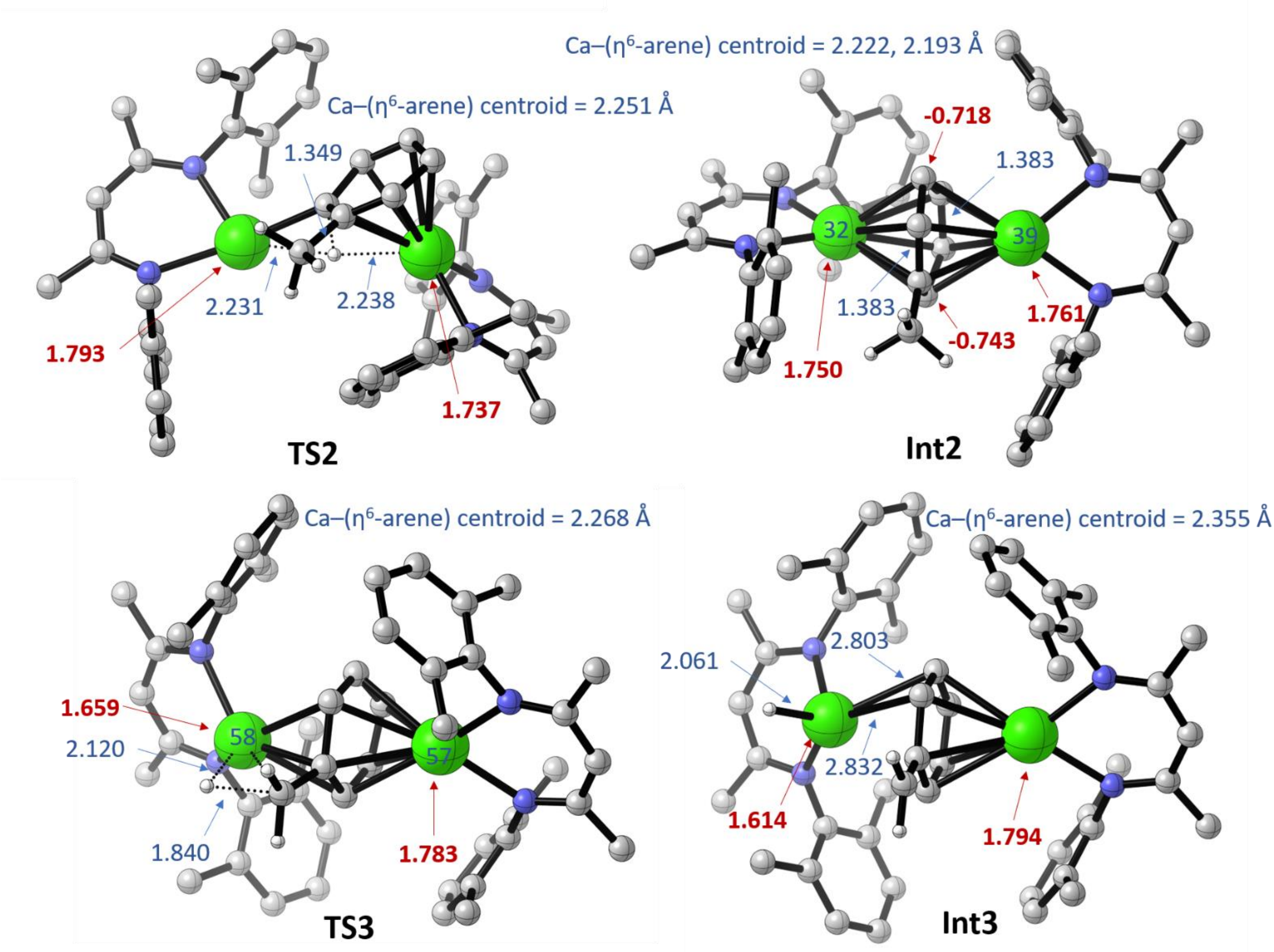

Figure S36:

Gibbs free energy (enthalpy) profile at the D3-B3PW91 level of theory (6-311++G** for Ca, 6-311G** N, O and 6-31G**) involving the nucleophilic attack of a Ph¯ anion at a Ph¯ anion for the formation of the biphenyl dianion complex **13** (**13opt**) starting from compound **3** (**3opt**)

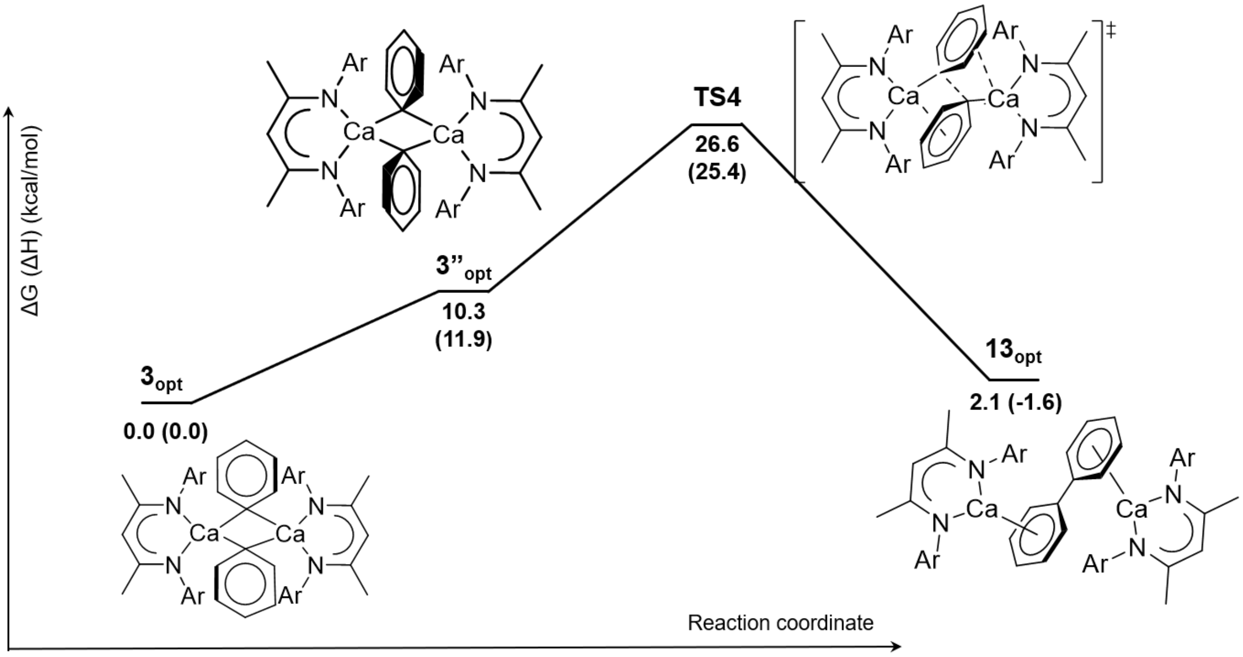

Figure S37:

Gibbs free energy speciation of complex **3** at the D3-B3PW91 level of theory (6-311++G** for Ca, 6-311G** N, O and 6-31G**).

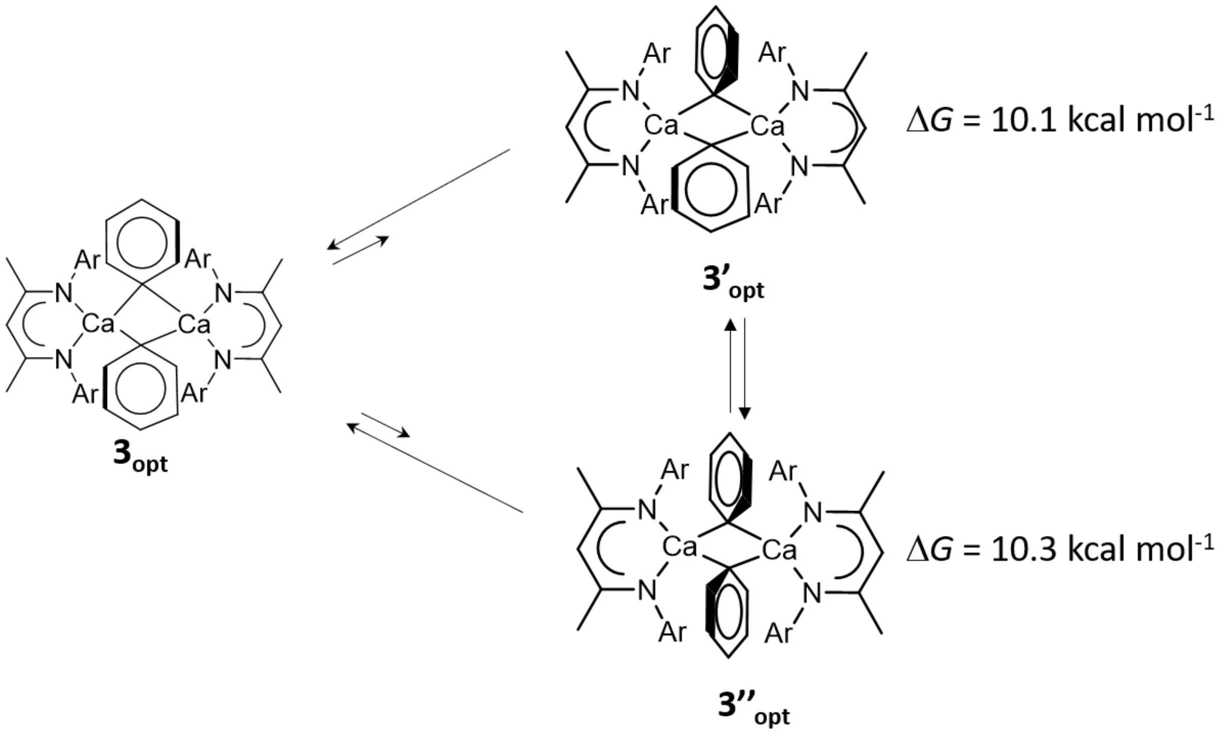

Figure S38:

AIM analyses of a) compound **3_{opt}** and b), c) compound **$3'_{opt}$**. Figure c and d show the AIM analysis along the Ca-C_{phenyl}-Ca plane with the phenyl ring located in the same plane (b) and perpendicular (c) with respect to the C_{phenyl}-Ca-C_{phenyl}-Ca plane.

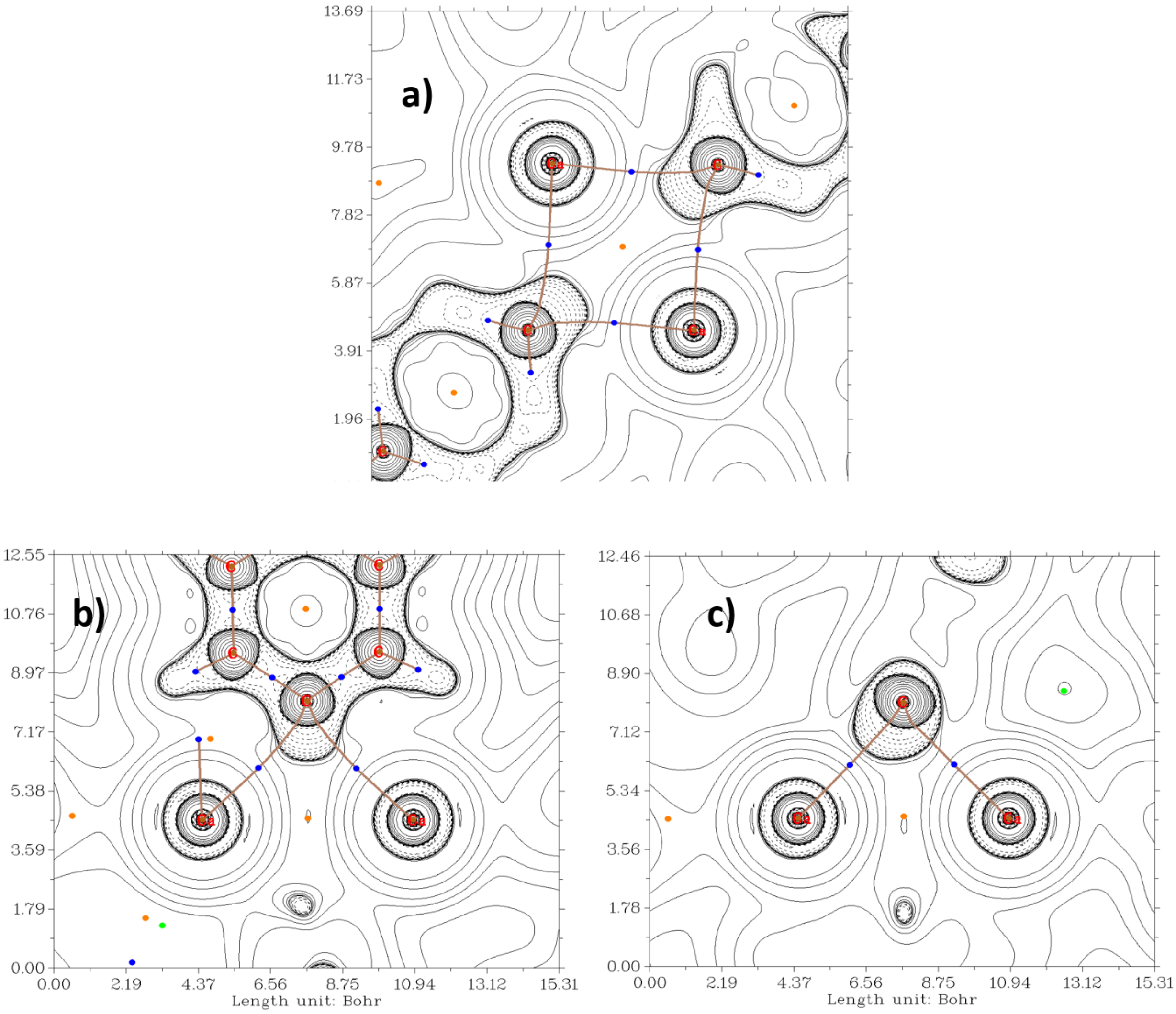

Figure S39:

AIM analyses of a) compound **TS4** and b) compound **TS2**.

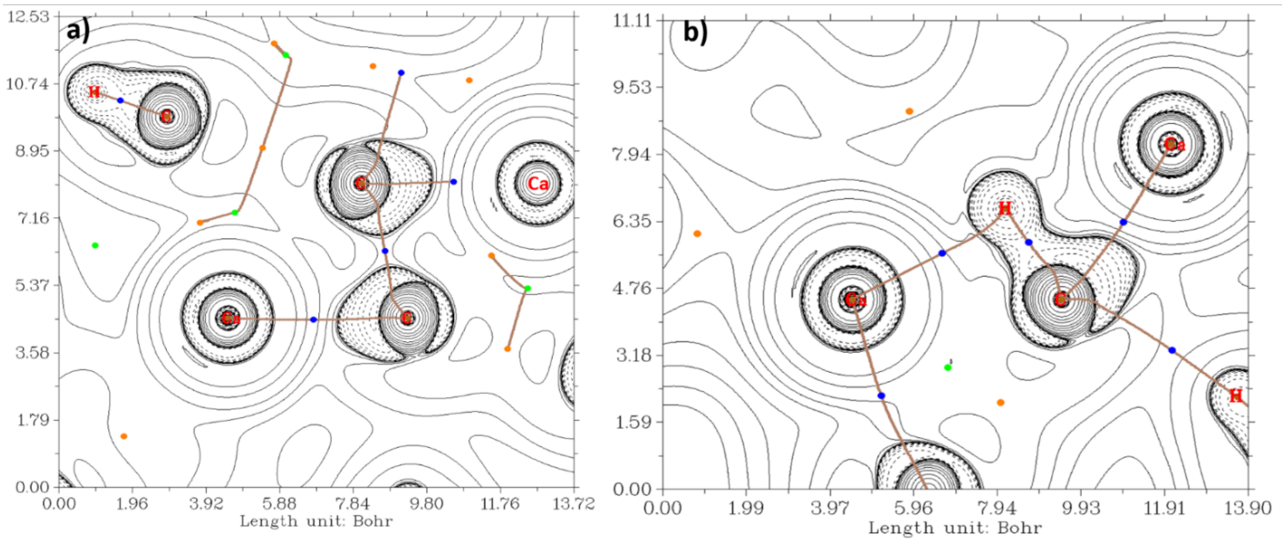

Energies and Atomic coordinates for the computed complexes and transition states

4opt (Enthalpies= -4103.408564 / Free Energies= -4103.602188)

Ca	2.409503	17.039143	3.279146
Ca	3.627767	13.635972	3.987638
N	4.396497	11.925211	2.536055
N	0.580447	17.756067	1.991383
N	3.417636	18.667249	1.932811
N	2.782688	11.734615	5.074625
C	3.431266	9.953340	3.569235
H	3.360680	8.877930	3.446019
C	4.644141	19.141366	2.461032
C	2.873859	10.449818	4.763880
C	1.773925	18.791994	0.162396
C	0.608331	18.296948	0.783833
C	0.963677	12.560736	6.480701
C	4.169024	10.616485	2.572026
C	6.679537	12.369406	1.790938
C	5.285487	12.428874	1.554495
C	4.781927	13.065682	0.397679
C	-0.637649	17.358350	2.593589
C	2.299290	12.112644	6.353431
C	7.208080	11.811720	3.100014
H	6.442350	11.151051	3.518182
C	-1.283615	16.154475	2.233307
C	7.550235	12.891713	0.834349
H	8.622520	12.849945	1.004228
C	-0.662588	18.432630	-0.029711
H	-0.742131	17.602425	-0.740939
H	-0.653913	19.360388	-0.608397
H	-1.552774	18.414833	0.602858
C	3.049928	19.018749	0.703657
C	-1.138708	18.151612	3.656504
C	0.053757	12.611780	5.265328
H	0.679969	12.896743	4.407655
C	4.611604	20.270861	3.314388
C	5.850360	18.448641	2.231802
C	0.516368	12.979180	7.735058
H	-0.507213	13.320584	7.852364
C	3.178756	12.156807	7.456225
C	2.689070	12.593237	8.689064
H	3.359415	12.634059	9.544167
C	5.803872	20.707669	3.892657
H	5.797258	21.575234	4.546151
C	4.045837	19.719275	-0.196461
H	4.589285	20.501555	0.341450
H	3.556620	20.160621	-1.067756
H	4.795121	19.002372	-0.552582
C	5.693367	13.559813	-0.541033
H	5.320734	14.031872	-1.445705
C	4.795756	9.725092	1.518804
H	4.746068	10.179042	0.525618
H	4.311992	8.745926	1.490586
H	5.859439	9.570470	1.738288
C	3.289635	13.201552	0.159606
H	2.793492	13.076278	1.128134
C	-0.444794	19.452111	4.019687
H	0.636545	19.274060	3.948490
C	-2.444578	15.788413	2.920538
H	-2.956640	14.870865	2.640718
C	-2.291489	17.734649	4.322689
H	-2.688130	18.335210	5.135449
C	-0.746114	15.250685	1.139380
H	0.111771	15.754486	0.684069
C	7.021235	18.922974	2.830720
H	7.957170	18.396832	2.660073
C	4.369163	16.823091	5.660929
C	7.065654	13.466298	-0.336958
H	7.756423	13.855475	-1.080259
C	3.294067	20.977244	3.577035
H	2.517152	20.202545	3.563236
C	1.365752	12.988288	8.837329
H	0.999529	13.322680	9.804423
C	5.874365	17.180234	1.402616

H	4.910050	17.097885	0.890298
C	3.605860	15.637017	5.490581
C	-2.949370	16.563561	3.956350
H	-3.848783	16.255118	4.482427
C	6.014814	15.952410	2.306650
H	6.975924	15.960808	2.831816
H	5.948360	15.032924	1.721164
H	5.230332	15.941380	3.074633
C	7.005784	20.046010	3.649537
H	7.925198	20.400801	4.107858
C	2.414196	9.401485	5.756205
H	3.227471	9.177407	6.456661
H	2.148185	8.470365	5.249008
H	1.564231	9.743625	6.351286
C	-0.237184	13.924529	1.713703
H	0.564795	14.100716	2.436327
H	0.167636	13.289747	0.916796
H	-1.043521	13.368869	2.206188
C	7.419982	12.950848	4.104992
H	8.164654	13.664874	3.734685
H	7.758725	12.566767	5.073619
H	6.496702	13.516779	4.280229
C	3.755747	18.070256	5.892330
H	4.370296	18.967028	5.929730
C	2.905156	14.583719	-0.371040
H	3.269542	15.373094	0.292440
H	1.815286	14.673648	-0.423547
H	3.296699	14.764536	-1.379173
C	-1.061164	13.648974	5.389503
H	-1.805681	13.355055	6.138924
H	-1.582807	13.752677	4.435778
H	-0.681275	14.639579	5.660728
C	8.489795	10.991710	2.940548
H	8.367283	10.206259	2.186813
H	8.754273	10.514894	3.891224
H	9.341391	11.613722	2.641137
C	-0.741665	19.940358	5.436861
H	-1.774924	20.293643	5.534741

H	-0.088964	20.785368	5.682857
H	-0.582807	19.157373	6.185881
C	1.588136	17.015099	6.010269
H	0.512244	17.065266	6.163736
C	2.213563	15.793400	5.721903
H	1.564090	14.918656	5.658833
C	-0.769109	20.558453	3.007609
H	-0.424092	20.297316	2.005060
H	-0.278462	21.495435	3.296526
H	-1.850357	20.736182	2.968352
C	2.369710	18.177252	6.050982
H	1.913044	19.147699	6.222681
C	-0.543870	11.241710	4.923115
H	0.230847	10.521137	4.652718
H	-1.229471	11.329260	4.071690
H	-1.107818	10.843347	5.775202
C	-1.788382	14.989106	0.045814
H	-2.622441	14.389179	0.428265
H	-1.337261	14.432251	-0.783905
H	-2.205918	15.919825	-0.350742
C	4.648198	11.808808	7.309972
H	4.799166	11.419616	6.296851
C	6.971311	17.181903	0.335601
H	6.903001	18.064260	-0.310069
H	6.887165	16.284312	-0.284664
H	7.970871	17.175504	0.785949
C	3.231607	21.688345	4.928829
H	3.527176	21.033011	5.754594
H	2.209016	22.034103	5.118297
H	3.879086	22.573007	4.955413
C	5.875275	16.777819	5.628387
H	6.226799	15.814102	5.252260
H	6.269739	16.895449	6.646691
H	6.302683	17.577803	5.014839
C	2.948272	21.962914	2.453120
H	3.747849	22.703166	2.328279
H	2.020984	22.499298	2.686974
H	2.800335	21.447564	1.501772

C	2.769437	12.097260	-0.767975
H	3.268277	12.138911	-1.744109
H	1.691194	12.211424	-0.933840
H	2.939717	11.104003	-0.341728
C	5.097167	10.726173	8.296191
H	5.030107	11.077645	9.332512
H	6.141001	10.447458	8.109285
H	4.481913	9.824219	8.211974
C	5.506390	13.071938	7.461619
H	5.172320	13.862889	6.779437
H	6.562726	12.852729	7.265626
H	5.428639	13.473198	8.479257
H	2.643815	15.025288	2.692246
H	1.632342	19.148133	-0.852520

161

5opt (Enthalpies= -4103.428924 / Free Energies= -4103.626670)

Ca	8.063085	19.928123	10.412475
H	7.282781	21.823190	10.226081
Ca	6.877646	23.555542	11.300888
H	8.380163	23.836267	13.737596
N	6.420005	18.249037	10.363655
N	8.012470	19.395694	8.102660
N	7.934717	25.509427	10.532374
N	5.123479	24.636164	10.158611
C	5.747446	17.740075	9.336950
C	6.057206	17.970899	7.988884
H	5.411376	17.470659	7.275423
C	7.139341	18.672056	7.418236
C	4.587964	16.810249	9.615367
H	4.935183	15.894874	10.107556
H	4.065949	16.532350	8.697157
H	3.874321	17.280182	10.299968
C	6.160445	17.779446	11.676872
C	5.336743	18.524051	12.548676
C	5.283858	18.162083	13.897112
H	4.665458	18.741888	14.578545
C	5.999920	17.072073	14.379267
H	5.956132	16.810793	15.433202
C	6.751385	16.300055	13.496931
H	7.285180	15.429341	13.870092
C	6.838300	16.629016	12.142968
C	4.481792	19.664225	12.031540
H	4.635746	19.720062	10.950635
C	2.994633	19.391303	12.281367
H	2.690798	18.422673	11.869793
H	2.386553	20.174786	11.819479
H	2.767586	19.380885	13.354102
C	4.876475	21.021082	12.617735
H	4.736133	21.051140	13.704737
H	4.261895	21.815019	12.179351
H	5.935716	21.232882	12.433270
C	7.665255	15.772513	11.200508
H	7.444403	16.093885	10.178242
C	9.165464	15.985723	11.423336
H	9.441808	17.029174	11.246883
H	9.754109	15.361609	10.740591
H	9.454988	15.733855	12.450867
C	7.307580	14.287088	11.311002
H	7.589924	13.873393	12.286028
H	7.836585	13.710815	10.543044
H	6.232210	14.124830	11.180062
C	7.259025	18.544133	5.913210
H	6.696430	19.344122	5.419171
H	6.844862	17.591641	5.572988
H	8.296779	18.622202	5.580170
C	9.096855	20.013737	7.432844
C	10.390710	19.481074	7.652588
C	11.484682	20.069684	7.015994
H	12.478619	19.658788	7.166553
C	11.318712	21.168102	6.178185
H	12.177619	21.611349	5.681589
C	10.050999	21.711808	6.003337
H	9.930018	22.592680	5.377651
C	8.928572	21.168138	6.634335
C	10.560082	18.236624	8.505904

H	9.767579	18.242179	9.267643
C	11.899395	18.165687	9.236422
H	12.730445	17.982332	8.545946
H	11.889395	17.339355	9.955801
H	12.110095	19.093304	9.779354
C	10.317127	16.972139	7.673687
H	9.309244	16.971104	7.249763
H	10.426319	16.074463	8.293550
H	11.037925	16.911926	6.849625
C	7.585643	21.858388	6.490017
H	6.831256	21.214092	6.948012
C	7.198056	22.100150	5.027185
H	7.846471	22.852061	4.562126
H	6.169927	22.475629	4.966877
H	7.267747	21.187835	4.425973
C	7.580359	23.183538	7.261004
H	7.751946	23.009767	8.326493
H	6.619795	23.698802	7.154226
H	8.361229	23.856391	6.887726
C	7.568058	26.381245	9.604749
C	6.290638	26.417259	9.012672
H	6.145537	27.202355	8.278906
C	5.141123	25.670959	9.329279
C	8.545276	27.441244	9.141461
H	9.160965	27.803322	9.969723
H	8.026391	28.287617	8.685269
H	9.228068	27.022263	8.393499
C	9.179664	25.630932	11.196242
C	10.371856	25.147745	10.615021
C	11.547764	25.175843	11.370734
H	12.468360	24.797836	10.932925
C	11.558160	25.663347	12.670916
H	12.480345	25.671583	13.245682
C	10.376668	26.131344	13.239944
H	10.390707	26.498778	14.260821
C	9.177302	26.121383	12.526969
C	10.391985	24.551757	9.221878
H	9.418925	24.744031	8.762850
C	11.468244	25.176150	8.328686
H	11.384433	26.267739	8.294271
H	11.379045	24.788143	7.307341
H	12.476443	24.932264	8.683407
C	10.570229	23.033141	9.283473
H	11.498511	22.770313	9.804259
H	10.608248	22.603265	8.280722
H	9.739789	22.566352	9.821817
C	7.885792	26.622294	13.152879
H	7.086268	25.937071	12.837986
C	7.499116	28.009841	12.625222
H	8.291565	28.736920	12.838928
H	6.578401	28.356745	13.109801
H	7.322478	27.994634	11.547676
C	7.904214	26.628395	14.680867
H	8.188460	25.653862	15.090741
H	6.908454	26.878523	15.061905
H	8.596604	27.382347	15.073950
C	3.843849	26.132058	8.703541
H	3.398798	25.339205	8.094537
H	3.989959	27.014042	8.076406
H	3.112590	26.372823	9.483634
C	3.889505	24.051704	10.535216
C	3.296550	23.025066	9.764147
C	2.131891	22.418444	10.239927
H	1.669179	21.629788	9.651646
C	1.557284	22.795475	11.449656
H	0.651750	22.307859	11.800705
C	2.155882	23.793766	12.209631
H	1.713239	24.085065	13.159074
C	3.317390	24.433203	11.771997
C	3.900882	22.560644	8.452913
H	4.738836	23.225920	8.224873
C	4.459144	21.139329	8.575640
H	5.295772	21.105249	9.279822
H	4.825312	20.775585	7.610188
H	3.689146	20.438030	8.917431
C	2.898164	22.635085	7.295501

H	2.086499	21.909328	7.423800
H	3.398112	22.404876	6.347410
H	2.442375	23.627292	7.208996
C	3.953500	25.514357	12.626540
H	4.813972	25.907010	12.073910
C	7.813596	22.908764	13.629406
H	7.084095	22.747554	14.423741
C	8.582053	21.780039	13.235821
C	9.780168	21.914906	12.455448
H	10.143446	22.916609	12.235299
C	10.499976	20.817394	12.006814
H	11.408204	20.982819	11.431655
C	10.082257	19.498899	12.283810
H	10.698732	18.650814	12.004871
C	8.926932	19.333817	13.074343
H	8.590701	18.337367	13.347499
C	8.187026	20.427653	13.508337
H	7.303025	20.250992	14.112968
C	2.996546	26.686023	12.867876
H	2.646875	27.110618	11.920810
H	3.498560	27.480205	13.432938
H	2.115368	26.373721	13.440753
C	4.462323	24.940591	13.954073
H	4.935494	25.721745	14.558956
H	5.202869	24.145387	13.802122
H	3.640920	24.511186	14.539937

161

TS1 (Enthalpies= -4103.329898 / Free Energies= -4103.520928)

Ca	2.677269	17.334157	3.269209
Ca	3.536543	13.855130	4.229939
N	4.111332	12.320986	2.523073
N	0.793442	17.532873	1.881209
N	3.400118	18.828516	1.652168
N	2.513725	11.906325	5.131332
C	2.610053	10.514920	3.137525
H	2.241712	9.564304	2.764858
C	4.496174	19.497198	2.234168
C	2.251387	10.783912	4.476251
C	1.792888	18.507718	-0.098479
C	0.750598	17.864740	0.594718
C	1.276422	12.588307	7.149057
C	3.543234	11.143688	2.289705
C	6.486551	12.677210	2.184297
C	5.162573	12.782189	1.689068
C	4.924825	13.408681	0.443720
C	-0.368211	17.041460	2.527540
C	2.367861	11.919057	6.546977
C	6.749790	12.070677	3.551131
H	5.873919	12.279585	4.180462
C	-0.719850	15.675463	2.472384
C	7.548537	13.118137	1.392515
H	8.566037	13.023593	1.758746
C	-0.488369	17.548095	-0.215268
H	-0.499745	16.484692	-0.480051
H	-0.516632	18.126450	-1.141603
H	-1.400647	17.743442	0.355524
C	2.990114	19.040975	0.415755
C	-1.111161	17.936894	3.336937
C	0.219779	13.264726	6.297978
H	0.741379	13.700230	5.441825
C	4.335433	20.804922	2.748563
C	5.692270	18.775848	2.466063
C	1.182303	12.602400	8.541914
H	0.343407	13.104619	9.013582
C	3.367673	11.329034	7.351136
C	3.233169	11.371743	8.741043
H	4.000351	10.916777	9.363173
C	5.367576	21.357291	3.508812
H	5.250250	22.358800	3.916613
C	3.789070	19.933345	-0.506445
H	4.841950	19.970613	-0.215421
H	3.399836	20.958057	-0.473155
H	3.713227	19.587715	-1.541449
C	6.020050	13.846746	-0.306674
H	5.843425	14.319643	-1.269593

C	3.901654	10.369285	1.037721	C	0.804710	13.753207	2.792604
H	4.954761	10.501701	0.775313	H	1.206851	14.379795	3.598746
H	3.308922	10.729050	0.189985	H	1.600070	13.138928	2.358765
H	3.696794	9.303238	1.160485	H	0.077691	13.078712	3.252926
C	3.531820	13.640041	-0.111850	C	7.972980	12.672050	4.244558
H	2.816868	13.196733	0.588484	H	8.907373	12.369409	3.758078
C	-0.696043	19.394089	3.429349	H	8.025057	12.331283	5.282688
H	0.401186	19.413669	3.388209	H	7.932350	13.767059	4.245997
C	-1.841540	15.236998	3.181438	C	4.418874	17.767254	5.729934
H	-2.119159	14.186712	3.137653	H	5.461611	18.068435	5.687413
C	-2.219977	17.453395	4.032814	C	3.228571	15.139255	-0.211690
H	-2.800470	18.130016	4.652487	H	3.277064	15.603291	0.776655
C	0.154029	14.667442	1.753748	H	2.230014	15.312325	-0.628816
H	0.952164	15.219460	1.251904	H	3.949337	15.646647	-0.863222
C	6.699832	19.374412	3.226198	C	-0.503802	14.407302	7.009540
H	7.623266	18.830002	3.405564	H	-1.195250	14.038170	7.777086
C	4.037224	16.426933	5.865853	H	-1.097340	14.974255	6.283261
C	7.324019	13.690664	0.145436	H	0.205658	15.088398	7.489605
H	8.159882	14.029699	-0.460758	C	6.853669	10.543370	3.469999
C	3.060642	21.594141	2.513032	H	5.922292	10.105795	3.099919
H	2.420545	21.008426	1.846566	H	7.057940	10.115761	4.458694
C	2.144757	11.992069	9.339650	H	7.666134	10.249351	2.794789
H	2.051672	12.013746	10.422376	C	-1.141079	20.071316	4.726099
C	5.893448	17.397997	1.863368	H	-2.222075	20.254673	4.735234
H	4.910379	17.006586	1.577924	H	-0.648404	21.044172	4.830024
C	2.670016	16.025020	5.844965	H	-0.901471	19.467583	5.608633
C	-2.595887	16.114596	3.950003	C	2.058841	18.379418	5.779141
H	-3.465385	15.757086	4.495143	H	1.298936	19.155870	5.764450
C	6.511966	16.391057	2.832772	C	1.682134	17.025742	5.869694
H	7.557650	16.629190	3.063221	H	0.624327	16.774701	5.905218
H	6.489887	15.392047	2.388817	C	-1.182067	20.205502	2.221500
H	5.957945	16.359817	3.776420	H	-0.743825	19.838511	1.290832
C	6.541530	20.651015	3.756015	H	-0.897238	21.258957	2.331579
H	7.333151	21.098205	4.351462	H	-2.274181	20.154386	2.136637
C	1.581920	9.622653	5.187742	C	3.412437	18.742662	5.679077
H	2.339293	8.901719	5.515960	H	3.687534	19.787927	5.573949
H	0.907325	9.096310	4.505524	C	-0.800803	12.261551	5.749669
H	1.025675	9.940604	6.071137	H	-0.327588	11.517723	5.104135

H	-1.568261	12.781681	5.163990
H	-1.301216	11.733996	6.570874
C	-0.591258	13.834328	0.709169
H	-1.367607	13.215128	1.173252
H	0.105581	13.161127	0.196454
H	-1.075694	14.463661	-0.044085
C	4.616588	10.712592	6.749190
H	4.505253	10.707995	5.660286
C	6.720356	17.489220	0.575351
H	6.235265	18.135779	-0.163049
H	6.842224	16.493353	0.137649
H	7.715730	17.900958	0.783286
C	2.279960	21.800013	3.814898
H	2.002529	20.839808	4.259589
H	1.357743	22.362614	3.628238
H	2.871464	22.356111	4.552292
C	4.790533	15.164839	6.151304
H	3.356887	14.797763	6.161447
H	4.988977	15.070235	7.224935
H	5.747717	15.006459	5.624234
C	3.347895	22.938524	1.835594
H	3.920006	23.603790	2.493018
H	2.411224	23.448412	1.581421
H	3.927773	22.804442	0.916618
C	3.339054	12.982181	-1.484497
H	3.948404	13.480741	-2.247505
H	2.291688	13.059866	-1.799444
H	3.620174	11.924666	-1.481255
C	4.840734	9.266996	7.203446
H	5.044025	9.211250	8.279403
H	5.701170	8.832064	6.681234
H	3.966167	8.641087	6.999794
C	5.836733	11.575776	7.089179
H	5.712672	12.597723	6.715996
H	6.746746	11.150256	6.651829
H	5.982877	11.636857	8.174106
H	3.432202	15.460412	2.878015
H	1.606626	18.703700	-1.149160

161

TS2 (Enthalpies= -4103.359148 / Free Energies= -4103.550495)

C	3.394614	-3.563186	0.289674
C	3.694314	-2.405220	1.046650
C	3.489517	-2.391037	2.446728
C	3.038789	-3.553815	3.073399
C	2.782488	-4.708546	2.342978
C	2.950191	-4.703722	0.961739
N	4.072714	-1.218352	0.380070
C	5.326940	-0.785927	0.437456
C	6.384562	-1.639015	1.106890
C	3.684084	-1.113920	3.244342
C	2.330409	-0.444915	3.508394
C	3.580695	-3.557066	-1.216848
C	5.055966	-3.719656	-1.604173
C	5.755345	0.462093	-0.047501
C	4.993463	1.531034	-0.559789
C	5.749926	2.782958	-0.942628
N	3.675609	1.508359	-0.699750
C	3.000978	2.632173	-1.245845
C	2.303899	3.498778	-0.376512
C	1.559240	4.550482	-0.916584
C	1.497245	4.748164	-2.291556
C	2.165944	3.872556	-3.141875
C	2.919852	2.808667	-2.645127
C	2.292295	3.242821	1.116882
C	2.528978	4.505383	1.949142
C	3.574029	1.825388	-3.596675
C	2.516818	0.884448	-4.187829
C	4.435941	-1.332466	4.559351
C	2.726493	-4.599727	-1.938369
C	4.376757	2.514621	-4.703508
C	0.964739	2.593958	1.524735
Ca	2.089348	-0.099791	-0.163591
C	0.209309	0.247518	-1.588207
C	-0.352268	-0.847918	-2.403870
C	-1.441387	-0.685697	-3.232166

C	-2.095987	0.572807	-3.386561
C	-1.416795	1.695537	-2.838232
C	-0.346365	1.554279	-1.976242
Ca	-2.304631	0.184799	-0.821682
N	-3.580243	1.309794	0.849984
C	-2.942236	2.414225	1.466100
C	-2.425416	2.282656	2.777425
C	-1.822823	3.387322	3.381314
C	-1.700658	4.598295	2.708690
C	-2.158200	4.703223	1.398900
C	-2.775117	3.625356	0.756292
C	-2.453908	0.945631	3.497325
C	-1.078052	0.281536	3.390699
C	-3.316587	3.770131	-0.653636
C	-4.808783	4.121884	-0.642876
C	-4.781989	0.956864	1.305717
C	-5.533491	1.882995	2.244811
C	-5.424346	-0.267630	1.053188
C	-4.917258	-1.460549	0.496259
C	-5.787748	-2.686129	0.695401
N	-3.762881	-1.547811	-0.138726
C	-3.243267	-2.773298	-0.613108
C	-2.122173	-3.321002	0.060853
C	-1.498012	-4.448676	-0.472300
C	-1.947758	-5.023749	-1.658070
C	-3.051289	-4.483926	-2.307941
C	-3.723939	-3.368541	-1.800624
C	-1.660897	-2.703556	1.369076
C	-2.594715	-3.108747	2.517196
C	-4.960909	-2.852460	-2.515200
C	-4.638334	-2.253884	-3.887563
C	-2.886370	1.052958	4.961625
C	-2.529414	4.767805	-1.502265
C	-6.019381	-3.955154	-2.647836
C	-0.216262	-3.023254	1.736489
C	0.341818	-2.165152	-2.285531
H	-1.804460	-1.549110	-3.787013
H	-2.873765	0.703743	-4.131262
H	0.123486	2.457168	-1.594102
H	-1.751264	2.696294	-3.103123
H	-0.135823	-0.029846	-0.314360
H	-6.202836	-2.702808	1.707467
H	-5.228486	-3.608791	0.525333
H	-6.632409	-2.669947	-0.003235
H	-6.412180	-0.361909	1.494288
H	-5.270509	1.665489	3.286440
H	-6.612457	1.737868	2.141931
H	-5.294231	2.933527	2.065038
H	-0.638187	-4.875320	0.035893
H	-1.440621	-5.892347	-2.069956
H	-3.406941	-4.942741	-3.227486
H	-1.431017	3.295401	4.391065
H	-1.231131	5.450066	3.193651
H	-2.041475	5.644079	0.869839
H	-3.166549	0.293595	2.984542
H	-3.004555	0.053017	5.394603
H	-2.144343	1.585437	5.568317
H	-3.839147	1.583588	5.062264
H	-1.083440	-0.709899	3.857332
H	-0.780061	0.164789	2.341133
H	-0.315760	0.888169	3.892781
H	-3.232293	2.790938	-1.142370
H	-2.872838	4.727092	-2.542066
H	-2.675784	5.798718	-1.158630
H	-1.456563	4.549220	-1.485806
H	-4.980612	5.061364	-0.103843
H	-5.182472	4.241724	-1.666969
H	-5.395188	3.335736	-0.160325
H	-1.743807	-1.612547	1.266192
H	-2.251832	-2.677044	3.464847
H	-3.615828	-2.763044	2.341300
H	-2.613373	-4.199748	2.626522
H	0.089528	-2.435484	2.606976
H	-0.079778	-4.077296	1.998809
H	0.481395	-2.804643	0.920291
H	-5.385421	-2.046446	-1.910022

H	-6.950028	-3.544836	-3.057416
H	-5.682399	-4.748348	-3.325269
H	-6.242773	-4.418114	-1.681437
H	-5.560620	-1.950365	-4.397562
H	-4.001964	-1.372018	-3.785920
H	-4.122528	-2.978778	-4.528843
H	6.806729	2.711604	-0.676286
H	5.314555	3.657846	-0.446940
H	5.674833	2.966721	-2.019735
H	6.820971	0.647963	0.030939
H	6.398182	-1.445024	2.186547
H	7.379071	-1.405547	0.717873
H	6.188658	-2.706280	0.975768
H	1.008436	5.211190	-0.252211
H	0.912316	5.567730	-2.700302
H	2.087637	4.010442	-4.216921
H	2.878296	-3.554808	4.148292
H	2.435157	-5.606856	2.846342
H	2.725453	-5.603204	0.397217
H	4.274846	-0.421064	2.638121
H	4.640341	-0.371302	5.044638
H	3.856279	-1.938707	5.264885
H	5.390943	-1.840499	4.388992
H	2.454216	0.525496	4.002298
H	1.762729	-0.271128	2.584573
H	1.695644	-1.075398	4.140557
H	3.275823	-2.564090	-1.576273
H	2.763929	-4.427950	-3.019288
H	3.097408	-5.616054	-1.758713
H	1.678027	-4.557612	-1.626637
H	5.459683	-4.655447	-1.199356
H	5.162005	-3.745965	-2.695159
H	5.660061	-2.890448	-1.229468
H	3.109275	2.546148	1.339811
H	2.608078	4.250132	3.012348
H	3.452660	5.009997	1.646660
H	1.702627	5.217436	1.846163
H	0.947300	2.354765	2.591661
H	0.123774	3.260811	1.316697
H	0.731819	1.670961	0.972657
H	4.268023	1.210193	-3.014752
H	4.914103	1.769217	-5.301233
H	3.726109	3.072442	-5.386983
H	5.111231	3.218326	-4.295067
H	2.987988	0.122802	-4.821750
H	1.946757	0.389301	-3.395576
H	1.795810	1.440910	-4.797394
H	-0.192429	-2.983394	-2.777520
H	1.355269	-2.105866	-2.709026
H	0.448928	-2.459479	-1.226236

161

Int2 (Enthalpies= -4103.400758 / Free Energies= -4103.593359)

C	3.150335	-2.343251	-2.643377
C	3.827367	-2.283775	-1.397624
C	3.963682	-3.442452	-0.598884
C	3.479698	-4.656966	-1.090228
C	2.858064	-4.734808	-2.330801
C	2.688089	-3.581550	-3.092177
N	4.215414	-1.022232	-0.904224
C	5.486441	-0.653918	-0.858861
C	6.557691	-1.587482	-1.381918
C	4.552499	-3.360860	0.797990
C	3.438334	-3.375281	1.853159
C	2.949763	-1.076887	-3.463352
C	4.261499	-0.579970	-4.084903
C	5.923320	0.580654	-0.341512
C	5.141706	1.612649	0.208935
C	5.851953	2.860365	0.679077
N	3.827831	1.540360	0.326893
C	3.039770	2.597870	0.827790
C	2.631351	2.573486	2.181989
C	1.633428	3.455687	2.599823
C	1.059052	4.362310	1.713676
C	1.509920	4.419753	0.398648
C	2.506593	3.558303	-0.063881

C	3.287581	1.624821	3.169674
C	3.881069	2.391211	4.357654
C	3.008815	3.669310	-1.491599
C	1.934102	3.284153	-2.511997
C	5.577030	-4.464366	1.074385
C	1.887557	-1.205958	-4.554996
C	3.551427	5.074732	-1.776992
C	2.337248	0.525940	3.655843
Ca	2.208947	-0.071916	-0.170352
C	0.255724	-1.084266	1.084417
C	0.106192	-1.747829	-0.197460
C	-0.026979	-1.010537	-1.360138
C	0.009239	0.484926	-1.324984
C	-0.123144	1.117429	-0.039650
C	-0.045397	0.386559	1.132176
Ca	-2.188892	-0.422028	-0.052150
N	-3.975087	1.011939	-0.526313
C	-3.334123	2.197422	-0.942801
C	-3.088952	3.239596	-0.019003
C	-2.300572	4.320145	-0.421617
C	-1.769185	4.385639	-1.704228
C	-2.012682	3.354490	-2.607584
C	-2.780811	2.246611	-2.247630
C	-3.610913	3.178546	1.405485
C	-2.468892	2.915148	2.392213
C	-3.045411	1.111510	-3.223880
C	-4.492968	1.137822	-3.732436
C	-5.275399	0.957864	-0.289657
C	-6.161820	2.138697	-0.626264
C	-5.914472	-0.148167	0.304669
C	-5.321234	-1.279891	0.890863
C	-6.249110	-2.282330	1.542917
N	-4.013647	-1.491961	0.898640
C	-3.428955	-2.605972	1.532053
C	-2.707481	-2.387842	2.734521
C	-2.023151	-3.461691	3.306017
C	-2.025375	-4.717663	2.705161
C	-2.705327	-4.911592	1.507143
C	-3.407630	-3.868831	0.898538
C	-2.701599	-1.007479	3.371395
C	-4.034178	-0.698632	4.065953
C	-4.064717	-4.063089	-0.455011
C	-3.016474	-4.013285	-1.573897
C	-4.382527	4.444757	1.790221
C	-2.084594	1.067920	-4.412097
C	-4.889505	-5.349671	-0.536196
C	-1.541996	-0.767667	4.336687
C	0.185980	-3.243622	-0.238614
H	-0.022656	-1.520001	-2.317665
H	-0.252104	1.044098	-2.210848
H	-0.042798	0.897782	2.087184
H	-0.251026	2.196134	0.006548
H	0.092296	-1.680708	1.974955
H	-7.203027	-1.821714	1.811881
H	-5.795076	-2.715782	2.438984
H	-6.460121	-3.112501	0.857823
H	-6.994396	-0.081032	0.387498
H	-6.321017	2.759060	0.263478
H	-7.144772	1.798461	-0.965645
H	-5.715439	2.773306	-1.395878
H	-1.468068	-3.315210	4.227540
H	-1.481670	-5.539795	3.162759
H	-2.676709	-5.887310	1.028999
H	-2.091647	5.118815	0.285549
H	-1.162510	5.236646	-2.002139
H	-1.580793	3.409756	-3.601753
H	-4.297327	2.330496	1.480758
H	-4.835751	4.329252	2.781683
H	-3.723504	5.319910	1.830278
H	-5.178683	4.662938	1.070578
H	-2.850607	2.830695	3.417036
H	-1.948090	1.986892	2.138663
H	-1.727552	3.720811	2.365652
H	-2.928249	0.163321	-2.673595
H	-2.260608	0.157283	-4.994905
H	-2.242376	1.919299	-5.084754

H	-1.035033	1.071039	-4.102569
H	-4.697049	2.082845	-4.249589
H	-4.665830	0.317520	-4.439445
H	-5.205633	1.031402	-2.912692
H	-2.604158	-0.265891	2.562728
H	-3.997654	0.292403	4.533710
H	-4.865009	-0.703747	3.357673
H	-4.237925	-1.439291	4.848273
H	-1.519132	0.288658	4.625905
H	-1.654339	-1.354311	5.256299
H	-0.574960	-1.015347	3.889768
H	-4.742105	-3.220524	-0.619769
H	-5.429095	-5.396636	-1.489035
H	-4.257392	-6.243092	-0.474867
H	-5.621844	-5.403467	0.276729
H	-3.493835	-4.090041	-2.558322
H	-2.444542	-3.076106	-1.546783
H	-2.295882	-4.833294	-1.476113
H	6.936708	2.770649	0.585578
H	5.603390	3.070285	1.725590
H	5.522946	3.729874	0.099174
H	6.992235	0.760041	-0.379158
H	6.806208	-2.347403	-0.631869
H	7.473903	-1.041668	-1.620933
H	6.214474	-2.119702	-2.274105
H	1.299685	3.433595	3.634780
H	0.278791	5.038363	2.052411
H	1.072988	5.143330	-0.284841
H	3.579143	-5.554513	-0.485082
H	2.489460	-5.688808	-2.698576
H	2.179735	-3.649676	-4.048945
H	5.063616	-2.398465	0.891262
H	6.056974	-4.302762	2.046432
H	5.108539	-5.455095	1.102768
H	6.357311	-4.488063	0.305658
H	3.857729	-3.264467	2.860714
H	2.720082	-2.560207	1.693493
H	2.879352	-4.318746	1.820237
H	2.604062	-0.280935	-2.782722
H	1.682369	-0.220469	-4.986423
H	2.226437	-1.856675	-5.370201
H	0.944954	-1.607998	-4.171460
H	4.690912	-1.352247	-4.734278
H	4.077939	0.313782	-4.693195
H	4.995953	-0.318548	-3.321455
H	4.113876	1.132122	2.646091
H	4.441772	1.711690	5.010256
H	4.560436	3.181654	4.020346
H	3.098272	2.862588	4.963483
H	2.835856	-0.110199	4.397023
H	1.444492	0.954718	4.126913
H	2.002193	-0.123199	2.837341
H	3.834371	2.959321	-1.606622
H	4.004045	5.114679	-2.774753
H	2.752366	5.824950	-1.744713
H	4.310338	5.369368	-1.043762
H	2.316959	3.389889	-3.534758
H	1.614144	2.247523	-2.364037
H	1.044193	3.915990	-2.412080
H	0.092709	-3.622366	-1.258717
H	1.153351	-3.573806	0.154465
H	-0.589458	-3.696416	0.389813

161

TS3 (Enthalpies= -4103.364503 / Free Energies= -4103.555644)

N	-4.133903	1.541733	0.273801
N	3.794317	1.648638	0.027944
C	-5.437236	1.391743	0.395110
C	-6.313795	2.607688	0.581613
C	-3.456320	2.768633	0.149997
C	-3.241264	3.300479	-1.149053
C	-2.312358	4.332318	-1.294356
H	-2.121876	4.750532	-2.277622
C	-1.615444	4.839131	-0.199417
H	-0.882009	5.626270	-0.340128
C	-1.873915	4.346633	1.073065

H	-1.339734	4.759743	1.924387
C	-2.800011	3.320708	1.273993
C	-4.023497	2.753905	-2.336593
H	-5.070600	2.666325	-2.017544
C	-3.571884	1.345749	-2.755031
H	-2.482363	1.291181	-2.868011
H	-3.912657	0.592829	-2.034616
C	-3.995893	3.683794	-3.550024
H	-2.999787	3.721730	-4.007281
H	-4.690098	3.316801	-4.313543
C	-3.126703	2.844054	2.676738
H	-3.844076	2.020860	2.583024
C	-1.901833	2.309182	3.424347
H	-1.483790	1.431961	2.922089
H	-1.108072	3.062709	3.492783
C	-3.801429	3.968773	3.472692
H	-4.676106	4.359612	2.942062
H	-4.126053	3.605718	4.454970
C	5.089824	1.404147	0.148646
C	6.042098	2.492034	0.605050
C	3.204703	2.820749	0.551123
C	3.041466	2.984836	1.946230
C	2.322233	4.087374	2.418008
H	2.190959	4.213717	3.490154
C	1.774948	5.021025	1.547458
H	1.225450	5.876493	1.932078
C	1.945962	4.856636	0.175580
H	1.528947	5.592796	-0.503883
C	2.647152	3.769106	-0.344278
C	3.593556	1.987570	2.948006
H	4.128820	1.208770	2.397046
C	2.461705	1.300793	3.717715
H	1.860647	2.026923	4.278022
H	1.797612	0.769489	3.032223
C	4.575143	2.649096	3.922555
H	4.062184	3.366956	4.573675
H	5.043704	1.894921	4.565887
C	2.850985	3.627915	-1.841588
H	2.784079	2.563184	-2.110219
C	1.797937	4.345155	-2.686004
H	0.779425	4.100580	-2.364492
H	1.911805	5.435223	-2.643185
C	4.259899	4.075864	-2.247534
H	5.022532	3.483733	-1.736131
H	4.404244	3.954202	-3.327436
Ca	-2.289073	0.167961	-0.068994
Ca	2.315771	0.234481	-1.176452
N	-4.100869	-1.257397	0.197811
N	3.810601	-1.354730	-0.306199
C	-6.052371	0.124067	0.404631
H	-7.131434	0.110083	0.511786
C	-5.410112	-1.125900	0.337879
C	-6.262894	-2.367538	0.435594
C	-3.419504	-2.494514	0.226060
C	-2.945838	-3.001413	1.460905
C	-2.009707	-4.037114	1.445933
H	-1.620751	-4.414775	2.388739
C	-1.556714	-4.586020	0.249783
H	-0.802715	-5.366131	0.256246
C	-2.079063	-4.128347	-0.955008
H	-1.747646	-4.579706	-1.887204
C	-3.016377	-3.093892	-0.991254
C	-3.439729	-2.439798	2.781240
H	-4.180917	-1.666353	2.555428
C	-2.314755	-1.774644	3.578290
H	-1.532403	-2.496561	3.840132
H	-1.845275	-0.974896	2.997499
C	-4.125498	-3.526747	3.617937
H	-3.412669	-4.304242	3.916825
H	-4.552704	-3.096552	4.531319
C	-3.610287	-2.644930	-2.314120
H	-4.325922	-1.844429	-2.095960
C	-2.558366	-2.081219	-3.273783
H	-2.060184	-1.195856	-2.865832
H	-1.781604	-2.823081	-3.493911
C	-4.381927	-3.793733	-2.975892

H	-5.139488	-4.205159	-2.300284
H	-4.884645	-3.446306	-3.886053
C	5.696047	0.152024	-0.098536
H	6.780259	0.158328	-0.043197
C	5.115128	-1.127370	-0.200835
C	6.073082	-2.300527	-0.181768
C	3.293087	-2.666898	-0.179398
C	3.171859	-3.513714	-1.301531
C	2.576359	-4.768831	-1.139966
H	2.484083	-5.427737	-2.000176
C	2.108367	-5.189252	0.097383
H	1.665794	-6.175996	0.209592
C	2.202383	-4.335498	1.193917
H	1.809606	-4.659899	2.152720
C	2.775351	-3.068447	1.077607
C	3.642158	-3.085130	-2.678764
H	4.162394	-2.126561	-2.571906
C	2.450212	-2.864107	-3.617821
H	1.871493	-3.788446	-3.736211
H	1.778216	-2.089336	-3.235991
C	4.626558	-4.087539	-3.291273
H	4.137220	-5.042971	-3.514818
H	5.033970	-3.697509	-4.231185
C	2.824655	-2.116058	2.259244
H	2.636133	-1.112305	1.856836
C	1.759900	-2.412994	3.316335
H	0.763862	-2.526650	2.876356
H	1.987030	-3.329904	3.874148
C	4.209697	-2.079951	2.919002
H	4.971762	-1.698342	2.236300
H	4.194634	-1.424326	3.797964
H	-3.110458	4.804566	3.634813
H	-4.289947	4.705333	-3.285813
H	-4.015382	1.067138	-3.717780
H	-3.708384	-4.612310	-3.255707
H	-3.021839	-1.789263	-4.223305
H	-4.931177	-4.015877	3.059761
H	-2.700776	-1.343097	4.509317
H	5.461553	-4.295187	-2.614074
H	2.789378	-2.538948	-4.607852
H	4.507599	-3.082562	3.249501
H	-2.173917	2.013966	4.444387
H	1.718364	-1.595565	4.043433
H	2.865318	0.576508	4.434796
H	5.365803	3.190545	3.394321
H	4.415740	5.131267	-1.993555
H	1.903928	4.044958	-3.733780
H	-7.328889	-2.128817	0.443374
H	-6.055523	-3.039005	-0.404730
H	-6.025948	-2.924443	1.349087
H	-7.353753	2.401070	0.316159
H	-6.288619	2.920579	1.633142
H	-5.951260	3.452675	-0.011934
H	6.911424	2.529085	-0.060262
H	5.565822	3.474515	0.621299
H	6.416488	2.274032	1.611014
H	7.061607	-2.007125	0.178948
H	5.690360	-3.110846	0.445475
H	6.185519	-2.707052	-1.193693
C	0.086876	1.242211	0.670040
C	-0.065852	-0.058721	1.254452
C	0.018232	-1.165015	0.347788
C	-0.052516	-1.005808	-1.019029
C	-0.008664	1.451233	-0.687874
C	-0.122914	0.333103	-1.654615
H	0.110243	-0.211816	2.309764
H	0.071372	-2.177685	0.735287
H	-0.044377	-1.887793	-1.652030
H	0.036144	2.465812	-1.070143
C	0.216593	0.521607	-2.994876
H	0.213451	2.111372	1.308705
H	2.038222	0.547612	-3.255051
H	0.139037	1.514361	-3.426204
H	0.110687	-0.296661	-3.699586

161

Int3 (Enthalpies= -4103.382121 / Free Energies= -4103.576388)

N	-4.035175	1.487186	-0.078980
N	3.731853	1.434899	0.373413
C	-5.328246	1.362660	0.125412
C	-6.240812	2.567849	0.091634
C	-3.346503	2.679562	-0.360748
C	-3.261021	3.147374	-1.696165
C	-2.344593	4.163613	-1.976853
H	-2.249271	4.535793	-2.991475
C	-1.533930	4.704958	-0.981495
H	-0.809936	5.474870	-1.230888
C	-1.643657	4.253446	0.329376
H	-0.992121	4.668384	1.091857
C	-2.542526	3.238676	0.663016
C	-4.147349	2.539841	-2.776617
H	-5.175433	2.552728	-2.391786
C	-3.805070	1.070932	-3.079060
H	-2.737743	0.934918	-3.293792
H	-4.114714	0.410322	-2.260449
C	-4.144519	3.340022	-4.079192
H	-3.172231	3.284948	-4.583193
H	-4.891791	2.930379	-4.766932
C	-2.674395	2.716899	2.081574
H	-2.919001	1.647198	2.012115
C	-1.399563	2.861304	2.906703
H	-0.527159	2.455380	2.388876
H	-1.179670	3.909627	3.136701
C	-3.855111	3.385686	2.797667
H	-4.794183	3.217842	2.264840
H	-3.963702	2.987646	3.813469
C	5.039108	1.222993	0.438127
C	5.966195	2.329173	0.900943
C	3.128989	2.561897	0.976459
C	2.637792	2.435896	2.301538
C	1.958954	3.510520	2.876042
H	1.588712	3.426743	3.893529
C	1.732574	4.685999	2.163965

H	1.201747	5.513475	2.628577
C	2.195656	4.792559	0.858914
H	2.023164	5.710302	0.301691
C	2.899995	3.749722	0.246447
C	2.869852	1.151130	3.074476
H	2.901112	0.347422	2.329861
C	1.766402	0.835212	4.085684
H	1.761357	1.546119	4.920542
H	0.768895	0.849361	3.634601
C	4.229208	1.158157	3.785066
H	4.294936	2.001184	4.483729
H	4.369052	0.230867	4.353465
C	3.416398	3.935811	-1.167583
H	3.879720	2.997748	-1.494408
C	2.287129	4.240495	-2.154920
H	1.524412	3.458238	-2.132945
H	1.803492	5.199367	-1.929225
C	4.483834	5.035704	-1.223821
H	5.295277	4.845311	-0.514834
H	4.913248	5.098683	-2.230319
Ca	-2.245009	0.060391	-0.485415
Ca	2.534158	0.303130	-1.343164
N	-3.967501	-1.268015	0.250460
N	3.822024	-1.453887	-0.441401
C	-5.924605	0.111205	0.388479
H	-6.993275	0.113627	0.575640
C	-5.262085	-1.118805	0.504605
C	-6.059444	-2.320836	0.947128
C	-3.267090	-2.469247	0.499145
C	-2.660785	-2.669225	1.762433
C	-1.765877	-3.730065	1.913088
H	-1.296729	-3.897739	2.878934
C	-1.458317	-4.571666	0.848320
H	-0.738199	-5.372892	0.976035
C	-2.088044	-4.390130	-0.378195
H	-1.859772	-5.064320	-1.200031
C	-3.013966	-3.362347	-0.568060
C	-2.974921	-1.757673	2.934769

H	-3.761166	-1.065628	2.615868
C	-1.760822	-0.916451	3.339594
H	-0.922499	-1.548109	3.653250
H	-1.405773	-0.297258	2.508668
C	-3.506658	-2.548841	4.135038
H	-2.740741	-3.216430	4.546491
H	-3.816888	-1.866687	4.934890
C	-3.760695	-3.225434	-1.881160
H	-4.504394	-2.431258	-1.750800
C	-2.839966	-2.810963	-3.030240
H	-2.389246	-1.825208	-2.870671
H	-2.019363	-3.526616	-3.159886
C	-4.511985	-4.516907	-2.225042
H	-5.162683	-4.829471	-1.401220
H	-5.131805	-4.372393	-3.117365
C	5.672519	0.017286	0.063547
H	6.756233	0.036592	0.121518
C	5.117892	-1.239408	-0.251232
C	6.095775	-2.385201	-0.409282
C	3.281890	-2.754806	-0.530973
C	3.133360	-3.397268	-1.778339
C	2.457860	-4.621443	-1.829419
H	2.337724	-5.119752	-2.788747
C	1.950812	-5.214950	-0.680395
H	1.438861	-6.172334	-0.738277
C	2.102659	-4.574171	0.547540
H	1.700006	-5.036248	1.443795
C	2.747840	-3.341700	0.643094
C	3.681097	-2.787919	-3.054957
H	4.214393	-1.867610	-2.789938
C	2.561023	-2.401688	-4.026111
H	1.966247	-3.278776	-4.311757
H	1.879912	-1.659354	-3.597732
C	4.685024	-3.725516	-3.735798
H	4.196292	-4.640175	-4.092571
H	5.141685	-3.232214	-4.601418
C	2.858942	-2.601428	1.962270
H	2.775237	-1.536260	1.713212

C	1.745952	-2.953302	2.948268
H	0.755666	-2.866244	2.490952
H	1.848149	-3.976042	3.331738
C	4.225651	-2.797097	2.629579
H	5.033783	-2.392459	2.017220
H	4.252914	-2.280492	3.596523
H	-3.694094	4.467915	2.868981
H	-4.383724	4.395274	-3.909573
H	-4.348386	0.724285	-3.965452
H	-3.818816	-5.340489	-2.431359
H	-3.395445	-2.758730	-3.973916
H	-4.368420	-3.164927	3.856692
H	-2.010765	-0.247207	4.170720
H	5.482968	-4.023652	-3.047631
H	2.978409	-1.955567	-4.934771
H	4.422179	-3.861669	2.805879
H	-1.506320	2.333897	3.860611
H	1.783457	-2.281058	3.811651
H	1.928451	-0.159160	4.514623
H	5.052679	1.238485	3.073232
H	4.054041	6.015172	-0.979147
H	2.679322	4.281285	-3.176044
H	-7.117076	-2.080228	1.076108
H	-5.967484	-3.133664	0.217960
H	-5.671989	-2.706734	1.896844
H	-6.949168	2.481981	-0.740774
H	-6.834114	2.629119	1.010227
H	-5.676616	3.496418	-0.026931
H	6.323148	2.881747	0.023225
H	5.455491	3.040496	1.554152
H	6.842204	1.929495	1.419135
H	7.018196	-2.209929	0.150214
H	5.654829	-3.332925	-0.088994
H	6.358733	-2.491422	-1.469104
C	0.142541	1.404899	-0.382899
C	0.287474	0.291744	0.487406
C	0.267407	-1.003837	-0.097683
C	0.026752	-1.195559	-1.447394

C -0.107951 1.244200 -1.735878

C -0.209462 -0.077498 -2.369237

H 0.551555 0.430067 1.526109

H 0.441388 -1.879203 0.519294

H 0.037252 -2.206007 -1.842832

H -0.223436 2.121154 -2.363138

C -0.498353 -0.241336 -3.693054

H 0.234281 2.410270 0.015921

H 3.148758 1.080082 -3.150614

H -0.584997 0.616511 -4.351396

H -0.525118 -1.226651 -4.144061

168

3opt (Enthalpies= -4295.034988 / Free Energies= -4295.235497)

Ca 3.783385 8.745539 9.698683

N 2.931156 9.332226 11.849580

N 4.305173 11.058593 9.782557

C 2.875956 10.465071 14.043704

H 2.674632 11.514858 14.275848

H 3.654810 10.121006 14.733804

H 1.974933 9.878786 14.233868

C 3.353113 10.323330 12.612375

C 4.252526 11.331814 12.190428

H 4.577292 12.001527 12.980410

C 4.620659 11.722174 10.894813

C 5.417184 13.003156 10.776479

H 6.372105 12.808745 10.275108

H 5.617671 13.445880 11.754543

H 4.889375 13.737124 10.160113

C 1.971701 8.401848 12.320401

C 2.317624 7.365496 13.215609

C 3.722089 7.205969 13.765500

H 4.328399 8.038090 13.395406

C 3.745352 7.231689 15.298551

H 3.254418 8.122422 15.702209

H 4.779477 7.211978 15.661961

H 3.233925 6.355647 15.714929

C 4.365345 5.914264 13.252275

H 3.823925 5.031638 13.611864

H 5.402773 5.841806 13.594541

H 4.377582 5.875431 12.161800

C 1.336858 6.432288 13.566261

H 1.594103 5.632594 14.256249

C 0.051249 6.498038 13.041835

H -0.694975 5.760748 13.326197

C -0.271532 7.507614 12.139057

H -1.271863 7.547936 11.719009

C 0.673039 8.461514 11.760756

C 0.318037 9.577110 10.795080

H 1.234921 9.852527 10.254681

C -0.108490 10.830277 11.569280

H -0.996011 10.619849 12.177881

H -0.348263 11.649901 10.882974

H 0.690370 11.167772 12.236082

C -0.732287 9.180021 9.758479

H -0.449463 8.262443 9.230820

H -0.846208 9.975729 9.015552

H -1.716061 9.019973 10.214833

C 4.481940 11.705116 8.529509

C 3.434151 12.500030 8.009910

C 2.202230 12.822986 8.838172

H 2.304731 12.308715 9.799021

C 2.097208 14.327533 9.115396

H 2.999749 14.710811 9.601754

H 1.243932 14.538293 9.771013

H 1.955062 14.893334 8.186818

C 0.917231 12.314867 8.178499

H 0.785102 12.737840 7.175538

H 0.041033 12.596191 8.773920

H 0.923736 11.225624 8.086221

C 3.551099 12.998840 6.710753

H 2.747082 13.605519 6.300088

C 4.674225 12.734729 5.933053

H 4.740539 13.120249 4.919174

C 5.712800 11.978138 6.463659

H 6.592329 11.771289 5.859064

C	5.638862	11.463607	7.759703
C	6.765361	10.611807	8.306353
H	6.558768	10.431140	9.365174
C	8.134375	11.287828	8.207433
H	8.430567	11.440925	7.163473
H	8.900007	10.660591	8.678573
H	8.135308	12.263809	8.705021
C	6.790214	9.260445	7.591745
H	5.766763	8.863826	7.527803
H	7.437198	8.551024	8.118270
H	7.149486	9.362415	6.565468
C	5.790408	7.301239	10.202160
C	6.198538	8.308313	11.103326
H	5.739949	9.302955	11.095995
C	7.190061	8.127716	12.068303
H	7.450266	8.945421	12.737102
C	7.840756	6.899921	12.172450
H	8.616227	6.747901	12.919116
C	7.471467	5.864891	11.318421
H	7.945056	4.890561	11.402392
C	6.468519	6.075827	10.369808
H	6.190118	5.188485	9.788256
Ca	5.298773	6.410529	7.869195
N	6.258143	5.809740	5.780768
N	4.741880	4.104692	7.770532
C	6.465110	4.658982	3.606887
H	6.697022	3.609251	3.404482
H	5.732037	4.982133	2.858900
H	7.369071	5.256395	3.472221
C	5.889446	4.812504	4.999931
C	4.959882	3.811792	5.371343
H	4.686438	3.134383	4.568426
C	4.500246	3.436413	6.643140
C	3.703006	2.152136	6.715149
H	2.784803	2.303278	7.292056
H	3.442080	1.786535	5.719453
H	4.270240	1.370906	7.231740
C	7.232973	6.752547	5.373953
C	6.933786	7.778503	4.448863
C	5.567754	7.914327	3.803640
H	4.952338	7.070525	4.129431
C	5.651037	7.890020	2.272862
H	6.185497	7.008568	1.905162
H	4.644646	7.889564	1.838014
H	6.172974	8.775752	1.891656
C	4.867142	9.192704	4.275031
H	5.428504	10.085278	3.975583
H	3.863816	9.260386	3.842505
H	4.756243	9.216784	5.360962
C	7.920468	8.727590	4.164169
H	7.699943	9.519097	3.452110
C	9.163580	8.692685	4.785618
H	9.913688	9.443914	4.552796
C	9.436607	7.697029	5.718869
H	10.404160	7.678553	6.211731
C	8.486810	6.723969	6.030083
C	8.802327	5.612473	7.013310
H	7.847911	5.241032	7.409930
C	9.442610	4.430997	6.274386
H	10.389863	4.735572	5.813424
H	9.644768	3.603371	6.963232
H	8.783088	4.063364	5.481791
C	9.657827	6.062794	8.197069
H	9.207011	6.913817	8.718512
H	9.759176	5.246126	8.919297
H	10.669461	6.348201	7.886421
C	4.476262	3.468944	9.013142
C	5.428858	2.575836	9.557386
C	6.655897	2.145392	8.771896
H	6.569083	2.552921	7.760012
C	6.753160	0.619371	8.660367
H	5.836374	0.184918	8.247905
H	7.587417	0.337066	8.007493
H	6.926240	0.155908	9.638721
C	7.938732	2.715800	9.383963
H	8.054613	2.398138	10.427198

H	8.819653	2.373311	8.828684
H	7.931497	3.808552	9.362812
C	5.234056	2.091311	10.852346
H	5.970558	1.416533	11.282706
C	4.120399	2.455325	11.602890
H	3.993418	2.076219	12.613385
C	3.171137	3.304946	11.048216
H	2.298615	3.593111	11.629621
C	3.327947	3.814621	9.757219
C	2.284181	4.751561	9.188098
H	2.592401	5.013217	8.172600
C	0.898743	4.107498	9.094613
H	0.513934	3.848252	10.087702
H	0.188600	4.802258	8.630913
H	0.923627	3.192236	8.493564
C	2.216076	6.037322	10.011205
H	3.237080	6.391754	10.212446
H	1.638787	6.803310	9.480418
H	1.749034	5.872155	10.984742
C	3.307078	7.856794	7.339441
C	2.986846	6.887700	6.362848
H	3.454169	5.896730	6.360770
C	2.075758	7.100768	5.327755
H	1.883304	6.310212	4.605566
C	1.422839	8.326346	5.220636
H	0.714536	8.506308	4.415722
C	1.705359	9.324690	6.148258
H	1.233692	10.299584	6.063577
C	2.623917	9.078862	7.170839
H	2.829361	9.942173	7.815283

168

3'opt (Enthalpies= -4295.018864 / Free Energies= -4295.219440)

Ca	1.579112	-0.470888	0.36563
Ca	-1.634397	0.530902	0.063248
N	2.249569	-2.677179	0.585996
N	3.915057	-0.234315	0.713660
N	-3.866653	-0.229142	-0.275261
N	-2.775678	2.571953	0.182557
C	0.696315	-0.267776	4.401544
C	0.833299	-0.232341	3.013155
C	-0.237029	-0.023947	2.115596
C	-1.481968	0.143803	2.763878
C	-1.657711	0.107673	4.147748
C	-0.559549	-0.097744	4.977348
C	3.404417	-4.690122	1.418902
H	2.683713	-5.234302	0.804762
H	3.219273	-4.940563	2.469249
H	4.412973	-5.036253	1.173012
C	3.290255	-3.197042	1.204698
C	4.376680	-2.424200	1.662879
H	5.145957	-2.980313	2.190398
C	4.715999	-1.099848	1.332814
C	6.136874	-0.700668	1.680471
H	6.241767	0.379926	1.798818
H	6.824439	-1.013370	0.887585
H	6.454617	-1.196366	2.602497
C	1.155568	-3.401725	0.066899
C	1.004034	-3.438177	-1.347364
C	-0.198310	-3.906475	-1.878423
H	-0.335351	-3.936881	-2.954351
C	-1.229725	-4.347015	-1.054137
H	-2.169284	-4.676810	-1.482327
C	-1.046569	-4.363813	0.321139
H	-1.841540	-4.740520	0.957005
C	0.135427	-3.906373	0.909807
C	4.489058	0.930464	0.142859
C	5.247507	0.830311	-1.045866
C	5.740745	1.998477	-1.631833
H	6.318767	1.927843	-2.549929
C	5.505020	3.243759	-1.065593
H	5.902047	4.142781	-1.529890
C	4.745660	3.335103	0.097144
H	4.553570	4.313500	0.523657
C	4.217959	2.197703	0.712059
C	-6.305794	-0.131503	-0.639341

H	-6.286186	-0.352924	-1.712069
H	-7.141066	0.544092	-0.443510
H	-6.493144	-1.081772	-0.135751
C	-4.987805	0.494368	-0.219619
C	-5.074355	1.845122	0.161267
H	-6.084709	2.231624	0.238709
C	-4.067270	2.819829	0.299991
C	-4.560022	4.223806	0.580988
H	-3.821982	4.977718	0.299211
H	-4.757418	4.328165	1.654299
H	-5.497301	4.426700	0.055385
C	-1.830079	3.621949	0.072413
C	-1.411431	3.989635	-1.230768
C	-0.397381	4.936994	-1.363567
H	-0.054570	5.218199	-2.354233
C	0.201154	5.511591	-0.245341
H	0.994345	6.244702	-0.368040
C	-0.219097	5.144542	1.027442
H	0.244705	5.601609	1.897751
C	-1.231662	4.198221	1.212943
H	-0.680444	-0.126976	6.056982
H	-2.650330	0.240117	4.572078
H	1.569703	-0.433646	5.027939
H	1.865864	-0.388537	2.670819
C	0.353777	0.752643	-1.462711
H	-2.418161	0.303803	2.210278
C	-4.023945	-1.555391	-0.761346
C	-3.816476	-1.800140	-2.136467
C	-4.171724	-3.048814	-2.653161
H	-4.051283	-3.247420	-3.713797
C	-4.679382	-4.043268	-1.825083
H	-4.976729	-5.002075	-2.242116
C	-4.744918	-3.831870	-0.450378
H	-5.072125	-4.644885	0.189184
C	-4.391496	-2.603916	0.116560
C	5.502482	-0.500762	-1.731421
C	-2.072431	3.364509	-2.446639
C	-3.211810	-0.725913	-3.030208
C	-4.340129	-2.426493	1.633277
C	-1.684034	3.844730	2.616605
C	3.365352	2.317962	1.965227
C	0.304306	-4.012357	2.414288
C	2.159118	-3.027544	-2.248318
C	0.539637	0.230230	-2.761015
C	1.439157	0.774958	-3.679905
C	2.213984	1.880778	-3.323242
C	1.148044	1.887570	-1.162811
C	2.066101	2.443823	-2.058717
H	-0.036656	-0.642370	-3.060782
H	1.551477	0.329875	-4.666628
H	2.933935	2.294818	-4.024116
H	1.044026	2.383033	-0.190984
H	2.665419	3.300326	-1.766525
C	4.738415	-0.577688	-3.056689
C	6.995298	-0.755002	-1.972049
H	5.121267	-1.298467	-1.086848
H	4.896496	-1.550103	-3.538067
H	3.665869	-0.433058	-2.907658
H	5.078107	0.200477	-3.750868
H	7.151476	-1.769515	-2.356888
H	7.404607	-0.056154	-2.711188
H	7.582764	-0.642841	-1.055260
C	2.884935	3.741209	2.233124
H	2.460077	1.708394	1.814931
C	4.058449	1.765251	3.215839
H	2.206775	3.742192	3.089044
H	3.721696	4.406599	2.477613
H	2.346554	4.157438	1.376405
H	3.405023	1.883528	4.088165
H	4.295676	0.704850	3.113485
H	4.989241	2.311338	3.410158
C	-0.827841	-3.344255	3.195063
C	0.400376	-5.486317	2.838443
H	1.234574	-3.503582	2.686205
H	-0.596343	-3.337899	4.265899
H	-0.979338	-2.313125	2.877649

H	-1.768797	-3.886049	3.064388
H	0.622423	-5.563169	3.909738
H	-0.554588	-5.995122	2.660170
H	1.170199	-6.031771	2.286911
C	1.789588	-2.917372	-3.726637
H	2.519435	-2.042889	-1.917858
C	3.340225	-3.999079	-2.108912
H	4.142018	-3.722818	-2.803175
H	3.756107	-3.992086	-1.101217
H	3.020877	-5.020733	-2.346730
H	2.631239	-2.493963	-4.283891
H	1.574305	-3.904466	-4.153633
H	0.921256	-2.276920	-3.897302
C	-4.375877	-3.760524	2.382974
H	-3.370918	-1.952877	1.847078
C	-5.420720	-1.523997	2.247063
H	-4.144326	-3.595850	3.440200
H	-5.372694	-4.216218	2.334308
H	-3.657154	-4.483776	1.988134
H	-5.304407	-1.513592	3.337688
H	-5.360580	-0.492280	1.899148
H	-6.423293	-1.910440	2.027745
C	-2.447865	-1.310888	-4.219228
C	-4.227293	0.314032	-3.517081
H	-2.472490	-0.178016	-2.426694
H	-1.866051	-0.523208	-4.709399
H	-1.760154	-2.101724	-3.901111
H	-3.125988	-1.732065	-4.970900
H	-3.745329	1.012033	-4.211483
H	-5.054702	-0.173221	-4.047097
H	-4.635724	0.899129	-2.690136
C	-1.180403	3.334752	-3.684924
H	-2.310036	2.324604	-2.185213
C	-3.402210	4.055745	-2.775638
H	-1.649651	2.721273	-4.462481
H	-1.040720	4.338093	-4.106464
H	-0.198976	2.911600	-3.464965
H	-3.843030	3.619777	-3.679872
H	-4.128724	3.948339	-1.967843
H	-3.242702	5.125739	-2.955735
C	-0.531918	3.342081	3.489754
C	-2.358905	5.042946	3.298055
H	-2.418114	3.035779	2.531640
H	-0.911883	2.972981	4.447939
H	0.011388	2.523807	3.010096
H	0.176139	4.151091	3.702381
H	-2.761286	4.752583	4.276042
H	-1.635524	5.850716	3.462023
H	-3.175722	5.450021	2.696428

168

3''opt (Enthalpies= -4295.015938 / Free Energies= -4295.219039)

Ca	2.121893	0.329290	0.141484
Ca	-2.218709	0.006244	-0.003760
N	3.562207	-1.022888	1.515750
N	4.235767	1.536572	0.034468
N	-3.550890	-1.242049	-1.549483
N	-4.224386	1.284456	-0.042027
C	-1.765774	0.995375	3.036149
C	-0.669660	1.226639	2.193285
C	0.108581	0.201321	1.599433
C	-0.367244	-1.106799	1.879813
C	-1.439651	-1.374798	2.742475
C	-2.139559	-0.318173	3.331797
C	5.508541	-1.825826	2.819238
H	5.652633	-1.421228	3.826735
H	6.502219	-2.039667	2.412347
H	4.954015	-2.761885	2.902486
C	4.801039	-0.811854	1.938144
C	5.587321	0.311253	1.619900
H	6.563270	0.327674	2.097404
C	5.352753	1.369582	0.729728
C	6.498370	2.361360	0.609925
H	6.335550	3.087957	-0.188474
H	7.447204	1.844686	0.432488
H	6.606148	2.910223	1.552916

C	2.829471	-2.141525	1.983174
C	2.500246	-3.163224	1.057570
C	1.657040	-4.200653	1.461632
H	1.395297	-4.984070	0.757773
C	1.151695	-4.251007	2.757854
H	0.496125	-5.064295	3.057504
C	1.497480	-3.257751	3.664884
H	1.100405	-3.296739	4.675949
C	2.328971	-2.194133	3.306191
C	4.206989	2.420886	-1.066026
C	4.870953	2.054466	-2.269497
C	4.696508	2.860412	-3.395696
H	5.188303	2.595554	-4.325770
C	3.914675	4.012705	-3.354739
H	3.793644	4.622473	-4.246055
C	3.309566	4.383478	-2.161497
H	2.723336	5.298982	-2.113212
C	3.440393	3.605592	-1.006442
C	-5.429318	-1.965723	-2.985658
H	-6.401721	-2.240578	-2.561748
H	-4.842279	-2.874446	-3.127704
H	-5.620499	-1.515960	-3.965257
C	-4.740844	-0.979258	-2.061563
C	-5.488506	0.184671	-1.793615
H	-6.412826	0.268050	-2.357949
C	-5.262142	1.217324	-0.872813
C	-6.324172	2.302725	-0.845327
H	-5.906384	3.260676	-0.526480
H	-7.133270	2.053750	-0.149400
H	-6.768980	2.420878	-1.837228
C	-4.272187	2.201790	1.031723
C	-3.365331	3.288978	1.065587
C	-3.356613	4.116244	2.191022
H	-2.675589	4.959662	2.234885
C	-4.201000	3.876558	3.271351
H	-4.170461	4.527139	4.141384
C	-5.083325	2.802669	3.230041
H	-5.738544	2.619600	4.077489

C	-5.145747	1.958924	2.119355
H	-2.962531	-0.512566	4.011208
H	-1.714683	-2.401657	2.966093
H	-2.327125	1.828175	3.452015
H	-0.407276	2.268048	2.014520
C	-0.315805	0.408874	-1.500171
H	0.141846	-1.968221	1.446746
C	-2.755986	-2.303002	-2.033021
C	-2.206358	-2.276660	-3.339413
C	-1.291101	-3.268720	-3.697932
H	-0.859053	-3.248195	-4.695133
C	-0.910363	-4.266623	-2.811063
H	-0.189854	-5.022493	-3.111100
C	-1.462902	-4.294830	-1.533608
H	-1.172267	-5.084180	-0.848494
C	-2.389366	-3.332795	-1.127465
C	5.816458	0.856301	-2.305486
C	-2.419858	3.540294	-0.100921
C	-2.573213	-1.224942	-4.373667
C	-3.067228	-3.428633	0.230828
C	-6.117788	0.788297	2.112633
C	2.828697	4.146976	0.276038
C	2.662693	-1.145344	4.353024
C	3.128307	-3.164742	-0.324887
C	0.386541	-0.780051	-1.837025
C	1.494065	-0.811206	-2.697867
C	1.979536	0.383559	-3.238834
C	0.236186	1.583682	-2.063837
C	1.352466	1.586787	-2.910154
H	0.034649	-1.739169	-1.449783
H	1.963895	-1.758059	-2.952508
H	2.835156	0.388416	-3.905948
H	-0.229296	2.548258	-1.854132
H	1.742617	2.517970	-3.309479
C	3.337162	-1.782832	5.575874
H	3.363159	-0.431341	3.909788
C	1.426977	-0.352858	4.793215
H	3.683485	-1.006001	6.267811

H	2.633916	-2.420845	6.124174
H	4.194100	-2.402606	5.295369
H	1.704853	0.379823	5.561016
H	0.975325	0.178119	3.953763
H	0.662762	-1.012462	5.219988
C	2.376041	-4.011166	-1.347077
H	3.135376	-2.131485	-0.698864
C	4.595574	-3.605678	-0.248927
H	4.666843	-4.609443	0.186322
H	5.040222	-3.632579	-1.250514
H	5.186326	-2.921896	0.364225
H	2.447656	-5.080752	-1.115486
H	1.316475	-3.746043	-1.400303
H	2.814295	-3.871199	-2.341600
C	3.901632	4.692760	1.225210
H	2.203123	4.995987	-0.031345
C	1.911360	3.188291	1.032497
H	3.436872	5.223818	2.064922
H	4.499374	3.874332	1.635385
H	4.573367	5.385724	0.708275
H	1.360716	3.727349	1.810078
H	1.157960	2.742277	0.369961
H	2.487640	2.413186	1.548840
C	6.703442	0.819895	-3.550490
H	6.492415	0.967364	-1.449896
C	5.135362	-0.504709	-2.124591
H	5.894507	-1.293985	-2.092866
H	4.581502	-0.548509	-1.184659
H	4.454682	-0.726621	-2.950748
H	7.440153	0.014945	-3.453384
H	6.121267	0.619348	-4.458468
H	7.246847	1.760176	-3.693825
C	-3.202383	-1.875672	-5.614542
H	-3.308068	-0.546976	-3.930524
C	-1.372152	-0.371174	-4.793950
H	-3.573506	-1.105511	-6.301064
H	-2.461698	-2.472679	-6.159816
H	-4.034073	-2.537970	-5.357506
H	-1.678480	0.351243	-5.560544
H	-0.958972	0.178830	-3.947901
H	-0.572565	-0.989352	-5.218227
C	-2.354462	-4.356996	1.210960
H	-3.082514	-2.428101	0.691623
C	-4.534320	-3.850753	0.078536
H	-4.596690	-4.835719	-0.398728
H	-5.019500	-3.912314	1.060001
H	-5.090734	-3.136312	-0.530986
H	-2.420403	-5.402701	0.887693
H	-1.296594	-4.100797	1.326239
H	-2.832044	-4.295780	2.195072
C	-1.322079	4.556907	0.209634
H	-1.909466	2.591700	-0.332609
C	-3.154790	3.965889	-1.377849
H	-2.433650	4.162560	-2.180084
H	-3.837920	3.190490	-1.726384
H	-3.725796	4.885035	-1.200544
H	-0.594826	4.580225	-0.608986
H	-1.734022	5.568240	0.310890
H	-0.784763	4.315374	1.130742
C	-7.500314	1.172870	2.647959
H	-6.247388	0.455431	1.080689
C	-5.559058	-0.416309	2.874273
H	-8.210352	0.354473	2.482473
H	-7.480905	1.372100	3.725822
H	-7.887196	2.069507	2.151343
H	-6.276353	-1.245906	2.860517
H	-4.628017	-0.765730	2.419360
H	-5.351851	-0.161256	3.920839

168

TS4 (Enthalpies= -4294.994521 / Free Energies= -4295.193015)

Ca	2.311092	0.249058	0.014926
Ca	-2.445711	-0.148946	0.178616
N	3.691070	-1.064277	1.463085
N	4.429442	1.431361	0.037546
N	-3.679159	-1.354885	-1.469999

N	-4.421584	1.189181	-0.086440
C	-1.759212	0.982645	2.648474
C	-0.723803	1.207584	1.747985
C	0.025884	0.158331	1.107323
C	-0.340086	-1.154435	1.571278
C	-1.359274	-1.389369	2.485234
C	-2.122854	-0.328713	3.007679
C	5.541248	-1.930007	2.851522
H	5.419825	-1.601073	3.889543
H	6.613637	-2.035169	2.664856
H	5.066080	-2.909200	2.759897
C	4.935677	-0.914135	1.901456
C	5.786512	0.149119	1.555631
H	6.770077	0.123236	2.015410
C	5.565564	1.225440	0.680030
C	6.738179	2.176740	0.520288
H	6.542497	2.950219	-0.225472
H	7.645546	1.631937	0.238257
H	6.947394	2.669391	1.476905
C	2.904570	-2.136871	1.944494
C	2.590766	-3.195706	1.056481
C	1.723913	-4.202997	1.486136
H	1.475635	-5.019541	0.815564
C	1.171353	-4.177667	2.763904
H	0.497831	-4.968815	3.082392
C	1.484913	-3.134353	3.626611
H	1.040788	-3.109833	4.618330
C	2.345705	-2.104215	3.242969
C	4.327159	2.369798	-1.007821
C	4.870520	2.053354	-2.282762
C	4.570867	2.893972	-3.356059
H	4.963497	2.666045	-4.341637
C	3.786379	4.033942	-3.192358
H	3.564739	4.670314	-4.044854
C	3.312590	4.362312	-1.928473
H	2.728511	5.269666	-1.787878
C	3.568350	3.546489	-0.822046
C	-5.488599	-2.196858	-2.932925
H	-6.329668	-2.692758	-2.433390
H	-4.768583	-2.962229	-3.228426
H	-5.888802	-1.719598	-3.833308
C	-4.881741	-1.168812	-1.992964
C	-5.704063	-0.064865	-1.713806
H	-6.652019	-0.051900	-2.244569
C	-5.492901	1.024599	-0.850790
C	-6.613347	2.050307	-0.840171
H	-6.317780	2.974150	-0.339023
H	-7.498149	1.655310	-0.328286
H	-6.912453	2.284697	-1.866820
C	-4.390590	2.202416	0.897179
C	-3.470011	3.270919	0.755567
C	-3.311791	4.165237	1.815262
H	-2.606688	4.985209	1.726254
C	-4.036430	4.021847	2.995866
H	-3.889360	4.721344	3.814490
C	-4.955543	2.986280	3.115211
H	-5.527171	2.882677	4.034364
C	-5.159501	2.072699	2.077966
H	-2.896423	-0.506827	3.744371
H	-1.561023	-2.407558	2.807032
H	-2.303596	1.825899	3.066584
H	-0.508436	2.244050	1.495680
C	-0.207573	0.208610	-0.886141
H	0.199639	-2.019485	1.189122
C	-2.795266	-2.320505	-1.989355
C	-2.224346	-2.147431	-3.277656
C	-1.252813	-3.055811	-3.697084
H	-0.803260	-2.942842	-4.678091
C	-0.834549	-4.103556	-2.882351
H	-0.073010	-4.795421	-3.231578
C	-1.396002	-4.263080	-1.620928
H	-1.065477	-5.085144	-0.994110
C	-2.380363	-3.386755	-1.155499
C	5.820650	0.870088	-2.448139
C	-2.690619	3.433948	-0.538698
C	-2.652166	-0.994334	-4.174317

C	-3.054043	-3.612013	0.187638
C	-6.186430	0.965764	2.248391
C	3.067234	4.009461	0.537129
C	2.636890	-0.975996	4.216441
C	3.233879	-3.249984	-0.318518
C	0.366112	-0.965586	-1.476594
C	1.358762	-0.921592	-2.453598
C	1.858243	0.307232	-2.915655
C	0.289768	1.431077	-1.443811
C	1.284233	1.487576	-2.413670
H	0.016396	-1.946214	-1.155490
H	1.742660	-1.851195	-2.867069
H	2.631061	0.356777	-3.673102
H	-0.111880	2.373699	-1.079907
H	1.637171	2.447801	-2.779526
C	3.111200	-1.506930	5.574827
H	3.439090	-0.364164	3.793837
C	1.422651	-0.059377	4.396695
H	3.443275	-0.678781	6.211789
H	2.302563	-2.022694	6.105987
H	3.940434	-2.214703	5.469922
H	1.653116	0.740877	5.111096
H	1.123825	0.397001	3.450908
H	0.557651	-0.615268	4.775200
C	2.475563	-4.119738	-1.317256
H	3.247112	-2.228611	-0.724349
C	4.698834	-3.695847	-0.231885
H	4.768897	-4.683623	0.238830
H	5.136871	-3.762079	-1.234809
H	5.297941	-2.992187	0.349830
H	2.549852	-5.184073	-1.063061
H	1.415608	-3.854950	-1.366131
H	2.905516	-3.998394	-2.317938
C	4.210776	4.453403	1.455496
H	2.451053	4.896060	0.334642
C	2.164739	3.012254	1.266265
H	3.811171	4.937400	2.354959
H	4.804352	3.592675	1.775139

H	4.874301	5.162124	0.949232
H	1.626209	3.498831	2.087074
H	1.400571	2.610150	0.591508
H	2.748977	2.200578	1.713592
C	6.605293	0.905679	-3.760471
H	6.559975	0.956243	-1.643592
C	5.174391	-0.510551	-2.277457
H	5.943217	-1.288221	-2.351029
H	4.706110	-0.613195	-1.296161
H	4.426829	-0.702897	-3.051697
H	7.362451	0.114079	-3.758015
H	5.956081	0.730956	-4.627337
H	7.116751	1.863600	-3.904687
C	-2.314463	-1.215033	-5.649289
H	-3.741246	-0.908329	-4.112069
C	-2.080453	0.346497	-3.702697
H	-2.775834	-0.426718	-6.254233
H	-1.233631	-1.168453	-5.828331
H	-2.681102	-2.181858	-6.012657
H	-2.415919	1.152309	-4.366759
H	-2.419764	0.586229	-2.692134
H	-0.987179	0.329696	-3.700232
C	-2.247432	-4.494508	1.137278
H	-3.183497	-2.637059	0.683704
C	-4.466537	-4.179966	0.001955
H	-4.423643	-5.142346	-0.521349
H	-4.948423	-4.338598	0.974271
H	-5.089879	-3.498879	-0.580497
H	-2.225639	-5.535142	0.792415
H	-1.214831	-4.147425	1.238521
H	-2.709720	-4.494719	2.130700
C	-1.524374	4.416403	-0.436247
H	-2.273670	2.450750	-0.806004
C	-3.602442	3.845697	-1.701888
H	-3.014261	3.968852	-2.618784
H	-4.367608	3.092452	-1.894196
H	-4.098105	4.798312	-1.480309
H	-0.953748	4.416773	-1.371059

H	-1.880807	5.440815	-0.275121
H	-0.839556	4.165257	0.380992
C	-7.549391	1.522170	2.675235
H	-6.317247	0.471742	1.282980
C	-5.708125	-0.114384	3.222137
H	-8.304516	0.727401	2.667161
H	-7.517879	1.933954	3.690709
H	-7.882348	2.320711	2.003899
H	-6.484534	-0.875585	3.366216
H	-4.813906	-0.609116	2.833251
H	-5.461998	0.311187	4.202761

168

13opt (Enthalpies= -4295.037609 / Free Energies= -4295.232122)

Ca	2.755252	0.238398	-0.266111
Ca	-2.839634	-0.223520	0.388114
N	3.939245	-1.108349	1.279534
N	4.813360	1.328758	0.011244
N	-3.891868	-1.438589	-1.320632
N	-4.723811	1.102519	-0.082571
C	-1.821279	1.079162	2.550356
C	-0.932462	1.239882	1.509677
C	-0.364715	0.089872	0.799395
C	-0.600145	-1.197687	1.453330
C	-1.478960	-1.316565	2.503812
C	-2.233917	-0.209581	2.991867
C	5.631833	-2.079036	2.787328
H	5.388712	-1.760435	3.807400
H	6.716063	-2.208879	2.732417
H	5.147031	-3.044869	2.625669
C	5.166274	-1.040038	1.786812
C	6.105613	-0.040842	1.474580
H	7.073384	-0.133327	1.957820
C	5.943611	1.076878	0.637347
C	7.135244	2.002014	0.502234
H	6.918429	2.846751	-0.156101
H	8.001850	1.459254	0.108275
H	7.423636	2.394065	1.484073
C	3.048829	-2.113385	1.726479
C	2.730915	-3.175503	0.843804
C	1.800304	-4.133640	1.252342
H	1.551179	-4.955841	0.589108
C	1.191718	-4.056637	2.502350
H	0.478666	-4.817087	2.809106
C	1.491070	-2.995749	3.349327
H	0.992711	-2.922902	4.312091
C	2.409461	-2.010321	2.982936
C	4.623844	2.346066	-0.934747
C	5.083879	2.159029	-2.265522
C	4.682565	3.077415	-3.236206
H	5.013419	2.953763	-4.262067
C	3.863828	4.159752	-2.919028
H	3.562975	4.859299	-3.694299
C	3.446261	4.346324	-1.607306
H	2.823751	5.201952	-1.353434
C	3.806138	3.450289	-0.596292
C	-5.509105	-2.295636	-2.980475
H	-6.400179	-2.808539	-2.598781
H	-4.744952	-3.046747	-3.189926
H	-5.796146	-1.811286	-3.919597
C	-5.039946	-1.275340	-1.960035
C	-5.913048	-0.196627	-1.736934
H	-6.826155	-0.210697	-2.324647
C	-5.763227	0.911823	-0.883586
C	-6.891239	1.923551	-0.917429
H	-6.542502	2.921887	-0.641872
H	-7.680004	1.644579	-0.209309
H	-7.342437	1.964336	-1.912628
C	-4.687700	2.188362	0.822171
C	-3.772366	3.245807	0.589211
C	-3.631880	4.233255	1.565462
H	-2.938469	5.052669	1.406212
C	-4.357544	4.185666	2.752884
H	-4.224657	4.960059	3.503854
C	-5.252893	3.145970	2.969862
H	-5.816442	3.110963	3.899069

C	-5.440664	2.141340	2.017608
H	-2.875875	-0.314731	3.857275
H	-1.614021	-2.294399	2.959133
H	-2.261169	1.964387	3.004004
H	-0.671596	2.244734	1.197755
C	0.189305	0.178653	-0.489324
H	-0.043349	-2.068354	1.126126
C	-2.910778	-2.345440	-1.764400
C	-2.216908	-2.111421	-2.980557
C	-1.205677	-3.001490	-3.341851
H	-0.670842	-2.853053	-4.273212
C	-0.854202	-4.075115	-2.528967
H	-0.072159	-4.760775	-2.842032
C	-1.501343	-4.262974	-1.313799
H	-1.208864	-5.092693	-0.678332
C	-2.534112	-3.411985	-0.911998
C	6.024627	1.005247	-2.599347
C	-2.970113	3.298772	-0.701146
C	-2.557683	-0.914905	-3.858454
C	-3.312402	-3.677795	0.365277
C	-6.418366	1.014457	2.302159
C	3.368364	3.754109	0.830388
C	2.671921	-0.840328	3.913087
C	3.445969	-3.293516	-0.491672
C	0.603543	-1.002698	-1.245957
C	1.398515	-0.898467	-2.366242
C	1.893700	0.353090	-2.828598
C	0.511214	1.452061	-1.136333
C	1.324469	1.515223	-2.248685
H	0.236704	-1.978635	-0.947061
H	1.669176	-1.809851	-2.893629
H	2.504590	0.425292	-3.719624
H	0.099645	2.371095	-0.736146
H	1.575236	2.489108	-2.660438
C	2.983043	-1.290464	5.344408
H	3.544850	-0.300101	3.534014
C	1.493032	0.137502	3.901203
H	3.288825	-0.432000	5.953573
H	2.103507	-1.733944	5.825381
H	3.786015	-2.035019	5.371004
H	1.710710	1.010187	4.529248
H	1.274661	0.486296	2.889262
H	0.578089	-0.334559	4.274099
C	2.691760	-4.138983	-1.513939
H	3.534024	-2.282764	-0.913591
C	4.878020	-3.816205	-0.323566
H	4.872959	-4.795535	0.169563
H	5.358523	-3.929168	-1.302802
H	5.487168	-3.132309	0.271060
H	2.710777	-5.203434	-1.249957
H	1.647008	-3.828737	-1.598029
H	3.161802	-4.041501	-2.499040
C	4.551973	4.184374	1.704903
H	2.693327	4.616979	0.749971
C	2.574655	2.649352	1.538387
H	4.195827	4.558902	2.672136
H	5.215257	3.337080	1.898465
H	5.134256	4.975823	1.221726
H	2.124905	3.030538	2.461540
H	1.736846	2.301951	0.919711
H	3.219954	1.812031	1.825064
C	6.673986	1.140058	-3.977303
H	6.838730	1.040368	-1.864872
C	5.385412	-0.386557	-2.470025
H	6.092171	-1.154099	-2.806862
H	5.139769	-0.618667	-1.429790
H	4.480817	-0.464552	-3.079488
H	7.431814	0.359797	-4.106186
H	5.940202	1.018594	-4.783567

References

1. A. S. S. Wilson, M. S. Hill, M. F. Mahon, C. Dinoi, L. Maron, *Science*, 2017, **358**, 1168-1171.
2. F. F. Blicke and F. D. Smith, *J. Am. Chem. Soc.*, 1929, **51**, 3479-3483.
3. K. G. Pearce, C. Dinoi, M. S. Hill, M. F. Mahon, L. Maron, R. S. Schwamm and A. S. S. Wilson, *Angew. Chem. Int. Ed.*, 2022, **61**, e202200305.
4. Dolomanov, O. V.; Bourhis, L.J.; Gildea, R.J.; Howard, J. A. K.; Puschmann, H. *J. Appl. Cryst.* **2009**, *42*, 339-341.
5. Sheldrick, G. M. *Acta Cryst.* **2015**, *A71*, 3-8.
6. Sheldrick, G. M. *Acta Cryst.* **201**, *C71*, 3-8.
7. Gaussian09, revision D.01. Frisch, M. J.; Trucks, G. W.; Schlegel, H. B.; Scuseria, G. E.; Robb, M. A.; Cheeseman, J. R.; Scalmani, G.; Barone, V.; Mennucci, B.; Petersson, G. A.; Nakatsuji, H.; Caricato, M.; Li, X.; Hratchian, H. P.; Izmaylov, A. F.; Bloino, J.; Zheng, G.; Sonnenberg, J. L.; Hada, M.; Ehara, M.; Toyota, K.; Fukuda, R.; Hasegawa, J.; Ishida, M.; Nakajima, T.; Honda, Y.; Kitao, O.; Nakai, H.; Vreven, T.; Montgomery, Jr., J. A.; Peralta, J. E.; Ogliaro, F.; Bearpark, M.; Heyd, J. J.; Brothers, E.; Kudin, K. N.; Staroverov, V. N.; Keith, T.; Kobayashi, R.; Normand, J.; Raghavachari, K.; Rendell, A.; Burant, J. C.; Iyengar, S. S.; Tomasi, J.; Cossi, M.; Rega, N.; Millam, J. M.; Klene, M.; Knox, J. E.; Cross, J. B.; Bakken, V.; Adamo, C.; Jaramillo, J.; Gomperts, R.; Stratmann, R. E.; Yazyev, O.; Austin, A. J.; Cammi, R.; Pomelli, C.; Ochterski, J. W.; Martin, R. L.; Morokuma, K.; Zakrzewski, V. G.; Voth, G. A.; Salvador, P.; Dannenberg, J. J.; Dapprich, S.; Daniels, A. D.; Farkas, O.; Foresman, J. B.; Ortiz, J. V.; Cioslowski, J.; Fox, D. J.; Gaussian, Inc., Wallingford CT, 2013.
8. (a) Perdew, J. P.; Chevary, J. A.; Vosko, S. H.; Jackson, K. A.; Pederson, M. R.; Singh, D. J.; Fiolhais, C. Atoms, Molecules, Solids, and Surfaces: Applications of the Generalized Gradient Approximation for Exchange and Correlation. *Phys. Rev. B* **1992**, *46*, 6671–6687. (b) Becke, A. D. Density-functional Thermochemistry. III. The Role of Exact Exchange. *J. Chem. Phys.* **1993**, *98*, 5648–5652.
9. Reed, A. E.; L. A. Curtiss, L. A.; Weinhold, F. Intermolecular interactions from a natural bond orbital, donor-acceptor viewpoint. *Chem. Rev.* **1988**, *88*, 899–926; b) Reed, A. E.; Weinhold, F. Natural bond orbital analysis of near-Hartree–Fock water dimer. *J. Chem. Phys.* **1983**, *78*, 4066–4073.
10. Grimme, S.; Ehrlich, S.; Goerigk, L. Effect of the Damping Function in Dispersion Corrected Density Functional Theory. *J. Comput. Chem.* **2011**, *32*, 1456–1465.
11. Marenich, A. V.; Cramer, C. J.; Truhlar, D. G. Universal Solvation Model Based on Solute Electron Density and on a Continuum Model of the Solvent Defined by the Bulk Dielectric Constant and Atomic Surface Tensions. *J. Phys. Chem. B* **2009**, *113*, 6378–6396.
