# Supplementary material for: Variable Ca‐Caryl Hapticity and its Consequences in Arylcalcium Dimers
Source: Adv Sci (Weinh). 2023 Sep 15;10(31):2304765. doi: 10.1002/advs.202304765 (PMC10625118; doi:10.1002/advs.202304765)

# checkCIF/PLATON report

You have not supplied any structure factors. As a result the full set of tests cannot be run.

THIS REPORT IS FOR GUIDANCE ONLY. IF USED AS PART OF A REVIEW PROCEDURE FOR PUBLICATION, IT SHOULD NOT REPLACE THE EXPERTISE OF AN EXPERIENCED CRYSTALLOGRAPHIC REFEREE.

No syntax errors found. CIF dictionary Interpreting this report

# Datablock: e21msh10-cpd-4

---

```
Bond precision:   C-C = 0.0044 A          Wavelength=0.71073

Cell:             a=12.4131(4)     b=41.3998(10)       c=13.7992(5)
                  alpha=90         beta=104.632(4)     gamma=90
Temperature:      150 K

                 Calculated                    Reported
Volume           6861.4(4)                     6861.4(4)
Space group      P 21/n                        P 1 21/n 1
Hall group       -P 2yn                        -P 2yn
Moiety formula   C65 H90 Ca2 N4 [+ solvent]    C65 H90 Ca2 N4
Sum formula      C65 H90 Ca2 N4 [+ solvent]    C70 H102 Ca2 N4
Mr               1007.57                       1079.71
Dx,g cm-3        0.975                         1.045
Z                4                             4
Mu (mm-1)        0.202                         0.206
F000             2192.0                        2360.0
F000'            2194.46
h,k,lmax         16,53,17                      16,53,17
Nref             15718                         14869
Tmin,Tmax        0.958,0.968                   0.706,1.000
Tmin'            0.944

Correction method= # Reported T Limits: Tmin=0.706 Tmax=1.000
AbsCorr = MULTI-SCAN

Data completeness= 0.946           Theta(max)= 27.483

R(reflections)= 0.0683( 8218)                        wR2(reflections)=
                                                     0.1723( 14869)
S = 1.002                    Npar= 669
```

The following ALERTS were generated. Each ALERT has the format
**test-name_ALERT_alert-type_alert-level.**
Click on the hyperlinks for more details of the test.

## Alert level B

```
PLAT029_ALERT_3_B _diffrn_measured_fraction_theta_full value Low .      0.957 Why?
```

**Author Response: diffraction very weak at higher angles, most likely due to the very disordered solvent content residing in structural channels.**

## Alert level C

```
PLAT222_ALERT_3_C NonSolvent Resd 1  H   Uiso(max)/Uiso(min) Range        6.2 Ratio
PLAT230_ALERT_2_C Hirshfeld Test Diff for    C10      --C14      .        6.5 s.u.
PLAT230_ALERT_2_C Hirshfeld Test Diff for    C41      --C52      .        5.5 s.u.
PLAT230_ALERT_2_C Hirshfeld Test Diff for    C42      --C43      .        5.5 s.u.
PLAT245_ALERT_2_C U(iso) H2        Smaller than U(eq) C2        by      0.012 Ang**2
```

## Alert level G

```
FORMU01_ALERT_1_G  There is a discrepancy between the atom counts in the
            _chemical_formula_sum and _chemical_formula_moiety. This is
            usually due to the moiety formula being in the wrong format.
            Atom count from _chemical_formula_sum:   C70 H102 Ca2 N4
            Atom count from _chemical_formula_moiety:C65 H90 Ca2 N4
FORMU01_ALERT_2_G  There is a discrepancy between the atom counts in the
            _chemical_formula_sum and the formula from the _atom_site* data.
            Atom count from _chemical_formula_sum:C70 H102 Ca2 N4
            Atom count from the _atom_site data:  C65 H90 Ca2 N4
CELLZ01_ALERT_1_G Difference between formula and atom_site contents detected.
CELLZ01_ALERT_1_G ALERT: Large difference may be due to a
            symmetry error - see SYMMG tests
           From the CIF: _cell_formula_units_Z    4
           From the CIF: _chemical_formula_sum  C70 H102 Ca2 N4
           TEST: Compare cell contents of formula and atom_site data

           atom    Z*formula  cif sites diff
           C        280.00    260.00   20.00
           H        408.00    360.00   48.00
           Ca         8.00      8.00    0.00
           N         16.00     16.00    0.00
PLAT013_ALERT_1_G N.O.K.   _shelx_hkl_checksum Found in CIF ......     Please Check
PLAT041_ALERT_1_G Calc. and Reported SumFormula    Strings  Differ     Please Check
PLAT164_ALERT_4_G Nr. of Refined C-H H-Atoms in Heavy-Atom Struct.          1 Note
PLAT303_ALERT_2_G Full Occupancy Atom H1        with # Connections       2.00 Check
PLAT606_ALERT_4_G Solvent Accessible VOID(S) in Structure ........          ! Info
PLAT868_ALERT_4_G ALERTS Due to the Use of _smtbx_masks Suppressed          ! Info
PLAT933_ALERT_2_G Number of HKL-OMIT Records in Embedded .res File          3 Note
PLAT941_ALERT_3_G Average HKL Measurement Multiplicity ..........         2.2 Low
```

0 **ALERT level A** = Most likely a serious problem - resolve or explain

1 **ALERT level B** = A potentially serious problem, consider carefully
5 **ALERT level C** = Check. Ensure it is not caused by an omission or oversight
12 **ALERT level G** = General information/check it is not something unexpected

5 ALERT type 1 CIF construction/syntax error, inconsistent or missing data
7 ALERT type 2 Indicator that the structure model may be wrong or deficient
3 ALERT type 3 Indicator that the structure quality may be low
3 ALERT type 4 Improvement, methodology, query or suggestion
0 ALERT type 5 Informative message, check

---

# Datablock: e21msh08-cpd-5

---

Bond precision: C-C = 0.0023 A Wavelength=0.71073

| | | | |
|---|---|---|---|
| Cell: | a=12.0271(3) | b=28.4491(7) | c=19.9507(5) |
| | alpha=90 | beta=98.561(3) | gamma=90 |
| Temperature: | 150 K | | |

| | Calculated | Reported |
|---|---|---|
| Volume | 6750.3(3) | 6750.3(3) |
| Space group | P 21/n | P 1 21/n 1 |
| Hall group | -P 2yn | -P 2yn |
| Moiety formula | C65 H90 Ca2 N4, C6 H14 | C65 H90 Ca2 N4, C6 H14 |
| Sum formula | C71 H104 Ca2 N4 | C71 H104 Ca2 N4 |
| Mr | 1093.74 | 1093.74 |
| Dx,g cm-3 | 1.076 | 1.076 |
| Z | 4 | 4 |
| Mu (mm-1) | 0.210 | 0.210 |
| F000 | 2392.0 | 2392.0 |
| F000’ | 2394.51 | |
| h,k,lmax | 17,40,28 | 16,40,28 |
| Nref | 20244 | 18018 |
| Tmin,Tmax | 0.912,0.946 | 0.922,1.000 |
| Tmin’ | 0.890 | |

Correction method= # Reported T Limits: Tmin=0.922 Tmax=1.000
AbsCorr = MULTI-SCAN

Data completeness= 0.890 Theta(max)= 30.310

R(reflections)= 0.0467( 13455) wR2(reflections)= 0.1231( 18018)

S = 1.017 Npar= 804

The following ALERTS were generated. Each ALERT has the format
**test-name_ALERT_alert-type_alert-level.**
Click on the hyperlinks for more details of the test.

## Alert level C

```
PLAT220_ALERT_2_C NonSolvent   Resd 1  C   Ueq(max)/Ueq(min) Range        3.6 Ratio
PLAT242_ALERT_2_C Low    'MainMol' Ueq as Compared to Neighbors of        C11 Check
PLAT242_ALERT_2_C Low    'MainMol' Ueq as Compared to Neighbors of        C63 Check
PLAT260_ALERT_2_C Large Average Ueq of Residue Including      C66      0.119 Check
PLAT260_ALERT_2_C Large Average Ueq of Residue Including     C66A      0.120 Check
PLAT362_ALERT_2_C Short  C(sp3)-C(sp2) Bond  C30      - C31      .       1.41 Ang.
```

## Alert level G

```
PLAT002_ALERT_2_G Number of Distance or Angle Restraints on AtSite         20 Note
PLAT003_ALERT_2_G Number of Uiso or Uij Restrained non-H Atoms ...         16 Report
PLAT013_ALERT_1_G N.O.K.   _shelx_hkl_checksum Found in CIF ......     Please Check
PLAT164_ALERT_4_G Nr. of Refined C-H H-Atoms in Heavy-Atom Struct.          2 Note
PLAT172_ALERT_4_G The CIF-Embedded .res File Contains DFIX Records          7 Report
PLAT176_ALERT_4_G The CIF-Embedded .res File Contains SADI Records         11 Report
PLAT178_ALERT_4_G The CIF-Embedded .res File Contains SIMU Records          2 Report
PLAT188_ALERT_3_G A Non-default SIMU Restraint Value has been used     0.0200 Report
PLAT188_ALERT_3_G A Non-default SIMU Restraint Value has been used     0.0200 Report
PLAT191_ALERT_3_G A Non-default SADI Restraint Value has been used     0.0050 Report
PLAT191_ALERT_3_G A Non-default SADI Restraint Value has been used     0.0400 Report
PLAT191_ALERT_3_G A Non-default SADI Restraint Value has been used     0.0400 Report
PLAT191_ALERT_3_G A Non-default SADI Restraint Value has been used     0.0400 Report
PLAT191_ALERT_3_G A Non-default SADI Restraint Value has been used     0.0400 Report
PLAT300_ALERT_4_G Atom Site Occupancy of C64       Constrained at       0.55 Check
PLAT300_ALERT_4_G Atom Site Occupancy of C65       Constrained at       0.55 Check
PLAT300_ALERT_4_G Atom Site Occupancy of C64A      Constrained at       0.45 Check
PLAT300_ALERT_4_G Atom Site Occupancy of C65A      Constrained at       0.45 Check
PLAT300_ALERT_4_G Atom Site Occupancy of H63       Constrained at       0.55 Check
PLAT300_ALERT_4_G Atom Site Occupancy of H64A      Constrained at       0.55 Check
PLAT300_ALERT_4_G Atom Site Occupancy of H64B      Constrained at       0.55 Check
PLAT300_ALERT_4_G Atom Site Occupancy of H64C      Constrained at       0.55 Check
PLAT300_ALERT_4_G Atom Site Occupancy of H65A      Constrained at       0.55 Check
PLAT300_ALERT_4_G Atom Site Occupancy of H65B      Constrained at       0.55 Check
PLAT300_ALERT_4_G Atom Site Occupancy of H65C      Constrained at       0.55 Check
PLAT300_ALERT_4_G Atom Site Occupancy of H63A      Constrained at       0.45 Check
PLAT300_ALERT_4_G Atom Site Occupancy of H64D      Constrained at       0.45 Check
PLAT300_ALERT_4_G Atom Site Occupancy of H64E      Constrained at       0.45 Check
PLAT300_ALERT_4_G Atom Site Occupancy of H64F      Constrained at       0.45 Check
PLAT300_ALERT_4_G Atom Site Occupancy of H65D      Constrained at       0.45 Check
PLAT300_ALERT_4_G Atom Site Occupancy of H65E      Constrained at       0.45 Check
PLAT300_ALERT_4_G Atom Site Occupancy of H65F      Constrained at       0.45 Check
PLAT300_ALERT_4_G Atom Site Occupancy of C66       Constrained at        0.5 Check
PLAT300_ALERT_4_G Atom Site Occupancy of C67       Constrained at        0.5 Check
PLAT300_ALERT_4_G Atom Site Occupancy of C68       Constrained at        0.5 Check
PLAT300_ALERT_4_G Atom Site Occupancy of C69       Constrained at        0.5 Check
PLAT300_ALERT_4_G Atom Site Occupancy of C70       Constrained at        0.5 Check
PLAT300_ALERT_4_G Atom Site Occupancy of C71       Constrained at        0.5 Check
PLAT300_ALERT_4_G Atom Site Occupancy of H66A      Constrained at        0.5 Check
PLAT300_ALERT_4_G Atom Site Occupancy of H66B      Constrained at        0.5 Check
PLAT300_ALERT_4_G Atom Site Occupancy of H66C      Constrained at        0.5 Check
```

```
PLAT300_ALERT_4_G Atom Site Occupancy of H67A       Constrained at        0.5 Check
PLAT300_ALERT_4_G Atom Site Occupancy of H67B       Constrained at        0.5 Check
PLAT300_ALERT_4_G Atom Site Occupancy of H68A       Constrained at        0.5 Check
PLAT300_ALERT_4_G Atom Site Occupancy of H68B       Constrained at        0.5 Check
PLAT300_ALERT_4_G Atom Site Occupancy of H69A       Constrained at        0.5 Check
PLAT300_ALERT_4_G Atom Site Occupancy of H69B       Constrained at        0.5 Check
PLAT300_ALERT_4_G Atom Site Occupancy of H70A       Constrained at        0.5 Check
PLAT300_ALERT_4_G Atom Site Occupancy of H70B       Constrained at        0.5 Check
PLAT300_ALERT_4_G Atom Site Occupancy of H71A       Constrained at        0.5 Check
PLAT300_ALERT_4_G Atom Site Occupancy of H71B       Constrained at        0.5 Check
PLAT300_ALERT_4_G Atom Site Occupancy of H71C       Constrained at        0.5 Check
PLAT300_ALERT_4_G Atom Site Occupancy of C66A       Constrained at        0.5 Check
PLAT300_ALERT_4_G Atom Site Occupancy of C67A       Constrained at        0.5 Check
PLAT300_ALERT_4_G Atom Site Occupancy of C68A       Constrained at        0.5 Check
PLAT300_ALERT_4_G Atom Site Occupancy of C69A       Constrained at        0.5 Check
PLAT300_ALERT_4_G Atom Site Occupancy of C70A       Constrained at        0.5 Check
PLAT300_ALERT_4_G Atom Site Occupancy of C71A       Constrained at        0.5 Check
PLAT300_ALERT_4_G Atom Site Occupancy of H66D       Constrained at        0.5 Check
PLAT300_ALERT_4_G Atom Site Occupancy of H66E       Constrained at        0.5 Check
PLAT300_ALERT_4_G Atom Site Occupancy of H66F       Constrained at        0.5 Check
PLAT300_ALERT_4_G Atom Site Occupancy of H67C       Constrained at        0.5 Check
PLAT300_ALERT_4_G Atom Site Occupancy of H67D       Constrained at        0.5 Check
PLAT300_ALERT_4_G Atom Site Occupancy of H68C       Constrained at        0.5 Check
PLAT300_ALERT_4_G Atom Site Occupancy of H68D       Constrained at        0.5 Check
PLAT300_ALERT_4_G Atom Site Occupancy of H69C       Constrained at        0.5 Check
PLAT300_ALERT_4_G Atom Site Occupancy of H69D       Constrained at        0.5 Check
PLAT300_ALERT_4_G Atom Site Occupancy of H70C       Constrained at        0.5 Check
PLAT300_ALERT_4_G Atom Site Occupancy of H70D       Constrained at        0.5 Check
PLAT300_ALERT_4_G Atom Site Occupancy of H71D       Constrained at        0.5 Check
PLAT300_ALERT_4_G Atom Site Occupancy of H71E       Constrained at        0.5 Check
PLAT300_ALERT_4_G Atom Site Occupancy of H71F       Constrained at        0.5 Check
PLAT301_ALERT_3_G Main Residue  Disorder ..............(Resd  1  )         3% Note
PLAT302_ALERT_4_G Anion/Solvent/Minor-Residue Disorder (Resd  2  )       100% Note
PLAT302_ALERT_4_G Anion/Solvent/Minor-Residue Disorder (Resd  3  )       100% Note
PLAT303_ALERT_2_G Full Occupancy Atom H1        with # Connections       2.00 Check
PLAT790_ALERT_4_G Centre of Gravity not Within Unit Cell: Resd.  #          2 Note
              C6 H14
PLAT790_ALERT_4_G Centre of Gravity not Within Unit Cell: Resd.  #          3 Note
              C6 H14
PLAT860_ALERT_3_G Number of Least-Squares Restraints .............        243 Note
PLAT933_ALERT_2_G Number of HKL-OMIT Records in Embedded .res File          3 Note
PLAT941_ALERT_3_G Average HKL Measurement Multiplicity ...........        3.7 Low
```

---

```
   0 ALERT level A = Most likely a serious problem - resolve or explain
   0 ALERT level B = A potentially serious problem, consider carefully
   6 ALERT level C = Check. Ensure it is not caused by an omission or oversight
  81 ALERT level G = General information/check it is not something unexpected

   1 ALERT type 1 CIF construction/syntax error, inconsistent or missing data
  10 ALERT type 2 Indicator that the structure model may be wrong or deficient
  10 ALERT type 3 Indicator that the structure quality may be low
  66 ALERT type 4 Improvement, methodology, query or suggestion
   0 ALERT type 5 Informative message, check
```

---

# Datablock: e22msh05-cpd-7

---

| | | | |
|---|---|---|---|
| Bond precision: | C-C = 0.0027 A | | Wavelength=0.71073 |
| Cell: | a=11.1859(5) | b=13.2501(5) | c=14.0992(6) |
| | alpha=102.251(3) | beta=110.173(4) | gamma=99.588(3) |
| Temperature: | 150 K | | |

| | Calculated | Reported |
|---|---|---|
| Volume | 1850.22(15) | 1850.22(14) |
| Space group | P -1 | P -1 |
| Hall group | -P 1 | -P 1 |
| Moiety formula | C72 H96 Ca2 N4, 2(C6 H6) | C72 H96 Ca2 N4, 2(C6 H6) |
| Sum formula | C84 H108 Ca2 N4 | C84 H108 Ca2 N4 |
| Mr | 1253.90 | 1253.90 |
| Dx,g cm-3 | 1.125 | 1.125 |
| Z | 1 | 1 |
| Mu (mm-1) | 0.200 | 0.200 |
| F000 | 680.0 | 680.0 |
| F000' | 680.66 | |
| h,k,lmax | 15,18,20 | 15,18,18 |
| Nref | 11097 | 9421 |
| Tmin,Tmax | 0.921,0.957 | 0.878,1.000 |
| Tmin' | 0.900 | |

Correction method= # Reported T Limits: Tmin=0.878 Tmax=1.000
AbsCorr = MULTI-SCAN

Data completeness= 0.849 Theta(max)= 30.320

R(reflections)= 0.0454( 7549) wR2(reflections)= 0.1200( 9421)

S = 1.053 Npar= 455

---

The following ALERTS were generated. Each ALERT has the format
**test-name_ALERT_alert-type_alert-level.**
Click on the hyperlinks for more details of the test.

---

**Alert level C**

```
PLAT220_ALERT_2_C NonSolvent   Resd 1  C   Ueq(max)/Ueq(min) Range        4.0 Ratio
PLAT222_ALERT_3_C NonSolvent Resd 1  H   Uiso(max)/Uiso(min) Range        4.7 Ratio
PLAT242_ALERT_2_C Low    'MainMol' Ueq as Compared to Neighbors of        C24 Check
PLAT250_ALERT_2_C Large U3/U1 Ratio for Average U(i,j) Tensor ....        2.9 Note
```

### Alert level G

```
PLAT002_ALERT_2_G Number of Distance or Angle Restraints on AtSite         12 Note
PLAT003_ALERT_2_G Number of Uiso or Uij Restrained non-H Atoms ...         12 Report
PLAT013_ALERT_1_G N.O.K.   _shelx_hkl_checksum Found in CIF ......     Please Check
PLAT176_ALERT_4_G The CIF-Embedded .res File Contains SADI Records         12 Report
PLAT178_ALERT_4_G The CIF-Embedded .res File Contains SIMU Records          1 Report
PLAT188_ALERT_3_G A Non-default SIMU Restraint Value has been used     0.0150 Report
PLAT191_ALERT_3_G A Non-default SADI Restraint Value has been used     0.0400 Report
PLAT191_ALERT_3_G A Non-default SADI Restraint Value has been used     0.0400 Report
PLAT191_ALERT_3_G A Non-default SADI Restraint Value has been used     0.0400 Report
PLAT191_ALERT_3_G A Non-default SADI Restraint Value has been used     0.0400 Report
PLAT191_ALERT_3_G A Non-default SADI Restraint Value has been used     0.0400 Report
PLAT191_ALERT_3_G A Non-default SADI Restraint Value has been used     0.0400 Report
PLAT300_ALERT_4_G Atom Site Occupancy of C37        Constrained at       0.75 Check
PLAT300_ALERT_4_G Atom Site Occupancy of C38        Constrained at       0.75 Check
PLAT300_ALERT_4_G Atom Site Occupancy of C39        Constrained at       0.75 Check
PLAT300_ALERT_4_G Atom Site Occupancy of C40        Constrained at       0.75 Check
PLAT300_ALERT_4_G Atom Site Occupancy of C41        Constrained at       0.75 Check
PLAT300_ALERT_4_G Atom Site Occupancy of C42        Constrained at       0.75 Check
PLAT300_ALERT_4_G Atom Site Occupancy of H37        Constrained at       0.75 Check
PLAT300_ALERT_4_G Atom Site Occupancy of H38        Constrained at       0.75 Check
PLAT300_ALERT_4_G Atom Site Occupancy of H39        Constrained at       0.75 Check
PLAT300_ALERT_4_G Atom Site Occupancy of H40        Constrained at       0.75 Check
PLAT300_ALERT_4_G Atom Site Occupancy of H41        Constrained at       0.75 Check
PLAT300_ALERT_4_G Atom Site Occupancy of H42        Constrained at       0.75 Check
PLAT300_ALERT_4_G Atom Site Occupancy of C37A       Constrained at       0.25 Check
PLAT300_ALERT_4_G Atom Site Occupancy of C38A       Constrained at       0.25 Check
PLAT300_ALERT_4_G Atom Site Occupancy of C39A       Constrained at       0.25 Check
PLAT300_ALERT_4_G Atom Site Occupancy of C40A       Constrained at       0.25 Check
PLAT300_ALERT_4_G Atom Site Occupancy of C41A       Constrained at       0.25 Check
PLAT300_ALERT_4_G Atom Site Occupancy of C42A       Constrained at       0.25 Check
PLAT300_ALERT_4_G Atom Site Occupancy of H37A       Constrained at       0.25 Check
PLAT300_ALERT_4_G Atom Site Occupancy of H38A       Constrained at       0.25 Check
PLAT300_ALERT_4_G Atom Site Occupancy of H39A       Constrained at       0.25 Check
PLAT300_ALERT_4_G Atom Site Occupancy of H40A       Constrained at       0.25 Check
PLAT300_ALERT_4_G Atom Site Occupancy of H41A       Constrained at       0.25 Check
PLAT300_ALERT_4_G Atom Site Occupancy of H42A       Constrained at       0.25 Check
PLAT302_ALERT_4_G Anion/Solvent/Minor-Residue Disorder (Resd  2  )       100% Note
PLAT302_ALERT_4_G Anion/Solvent/Minor-Residue Disorder (Resd  3  )       100% Note
PLAT303_ALERT_2_G Full Occupancy Atom H31       with # Connections       2.00 Check
PLAT303_ALERT_2_G Full Occupancy Atom H35       with # Connections       2.00 Check
PLAT343_ALERT_2_G Unusual          Angle Range in Main Residue for        C30 Check
PLAT413_ALERT_2_G Short Inter XH3 .. XHn     H26A     ..H39A     .       2.09 Ang.
                                               1-x,2-y,2-z  =      2_677 Check
PLAT860_ALERT_3_G Number of Least-Squares Restraints ............        192 Note
PLAT941_ALERT_3_G Average HKL Measurement Multiplicity ..........        1.8 Low
PLAT952_ALERT_5_G Calculated (ThMax) and CIF-Reported Lmax Differ.          2 Units
```

---

```
   0 ALERT level A = Most likely a serious problem - resolve or explain
   0 ALERT level B = A potentially serious problem, consider carefully
   4 ALERT level C = Check. Ensure it is not caused by an omission or oversight
  45 ALERT level G = General information/check it is not something unexpected

   1 ALERT type 1 CIF construction/syntax error, inconsistent or missing data
```

```
 9 ALERT type 2 Indicator that the structure model may be wrong or deficient
10 ALERT type 3 Indicator that the structure quality may be low
28 ALERT type 4 Improvement, methodology, query or suggestion
 1 ALERT type 5 Informative message, check
```

---

# Datablock: s22msh16-cpd-8

---

Bond precision: C-C = 0.0047 A Wavelength=1.54184

| | | | |
|---|---|---|---|
| Cell: | a=14.31188(14) | b=14.31188(14) | c=31.9348(4) |
| | alpha=90 | beta=90 | gamma=90 |
| Temperature: | 150 K | | |

| | Calculated | Reported |
|---|---|---|
| Volume | 6541.20(15) | 6541.20(15) |
| Space group | P 43 21 2 | P 43 21 2 |
| Hall group | P 4nw 2abw | P 4nw 2abw |
| Moiety formula | C72 H96 Ca2 N4 | C36 H48 Ca N2 |
| Sum formula | C72 H96 Ca2 N4 | C36 H48 Ca N2 |
| Mr | 1097.69 | 548.84 |
| Dx,g cm-3 | 1.115 | 1.115 |
| Z | 4 | 8 |
| Mu (mm-1) | 1.824 | 1.824 |
| F000 | 2384.0 | 2384.0 |
| F000’ | 2392.32 | |
| h,k,lmax | 18,18,40 | 17,17,39 |
| Nref | 6888[ 3977] | 6340 |
| Tmin,Tmax | 0.691,0.846 | 0.645,1.000 |
| Tmin’ | 0.507 | |

Correction method= # Reported T Limits: Tmin=0.645 Tmax=1.000
AbsCorr = MULTI-SCAN

Data completeness= 1.59/0.92 Theta(max)= 76.620

R(reflections)= 0.0408( 5350) wR2(reflections)= 0.1115( 6340)

S = 1.009 Npar= 369

---

The following ALERTS were generated. Each ALERT has the format
**test-name_ALERT_alert-type_alert-level.**
Click on the hyperlinks for more details of the test.

---

### Alert level C

```
PLAT042_ALERT_1_C Calc. and Reported MoietyFormula Strings  Differ     Please Check
PLAT220_ALERT_2_C NonSolvent   Resd 1  C   Ueq(max)/Ueq(min) Range        3.6 Ratio
PLAT222_ALERT_3_C NonSolvent Resd 1  H   Uiso(max)/Uiso(min) Range        4.2 Ratio
PLAT242_ALERT_2_C Low    'MainMol' Ueq as Compared to Neighbors of        C38 Check
```

---

### Alert level G

```
PLAT013_ALERT_1_G N.O.K.   _shelx_hkl_checksum Found in CIF ......     Please Check
PLAT045_ALERT_1_G Calculated and Reported Z Differ by a Factor ...      0.500 Check
PLAT300_ALERT_4_G Atom Site Occupancy of H34A       Constrained at        0.5 Check
PLAT300_ALERT_4_G Atom Site Occupancy of H34B       Constrained at        0.5 Check
PLAT300_ALERT_4_G Atom Site Occupancy of H34C       Constrained at        0.5 Check
PLAT300_ALERT_4_G Atom Site Occupancy of H39A       Constrained at        0.5 Check
PLAT300_ALERT_4_G Atom Site Occupancy of H39B       Constrained at        0.5 Check
PLAT300_ALERT_4_G Atom Site Occupancy of H39C       Constrained at        0.5 Check
PLAT303_ALERT_2_G Full Occupancy Atom H31       with # Connections       2.00 Check
PLAT343_ALERT_2_G Unusual          Angle Range in Main Residue for        C30 Check
PLAT343_ALERT_2_G Unusual sp?      Angle Range in Main Residue for        C35 Check
PLAT367_ALERT_2_G Long?  C(sp?)-C(sp?) Bond  C33      - C34      .       1.50 Ang.
PLAT367_ALERT_2_G Long?  C(sp?)-C(sp?) Bond  C38      - C39      .       1.52 Ang.
PLAT764_ALERT_4_G Overcomplete CIF Bond List Detected (Rep/Expd) .       1.11 Ratio
PLAT789_ALERT_4_G Atoms with Negative _atom_site_disorder_group  #          6 Check
PLAT822_ALERT_4_G CIF-embedded .res Contains Negative PART Numbers          2 Check
PLAT933_ALERT_2_G Number of HKL-OMIT Records in Embedded .res File          9 Note
```

---

0 **ALERT level A** = Most likely a serious problem - resolve or explain
0 **ALERT level B** = A potentially serious problem, consider carefully
4 **ALERT level C** = Check. Ensure it is not caused by an omission or oversight
17 **ALERT level G** = General information/check it is not something unexpected

3 ALERT type 1 CIF construction/syntax error, inconsistent or missing data
8 ALERT type 2 Indicator that the structure model may be wrong or deficient
1 ALERT type 3 Indicator that the structure quality may be low
9 ALERT type 4 Improvement, methodology, query or suggestion
0 ALERT type 5 Informative message, check

---

# Datablock: s22msh81-cpd-9

---

Bond precision: C-C = 0.0055 A Wavelength=1.54184

| | | | |
|---|---|---|---|
| Cell: | a=12.1327(6) | b=14.7627(9) | c=19.3476(7) |
| | alpha=105.414(5) | beta=90.169(4) | gamma=93.676(5) |
| Temperature: | 150 K | | |

| | Calculated | Reported |
|---|---|---|
| Volume | 3333.1(3) | 3333.1(3) |
| Space group | P -1 | P -1 |
| Hall group | -P 1 | -P 1 |
| Moiety formula | C65 H90 Ca2 N4, C7 H8 | C65 H90 Ca2 N4, C7 H8 |
| Sum formula | C72 H98 Ca2 N4 | C72 H98 Ca2 N4 |
| Mr | 1099.70 | 1099.70 |
| Dx,g cm-3 | 1.096 | 1.096 |
| Z | 2 | 2 |
| Mu (mm-1) | 1.790 | 1.790 |
| F000 | 1196.0 | 1196.0 |
| F000' | 1200.16 | |
| h,k,lmax | 14,18,23 | 14,18,23 |
| Nref | 13130 | 13018 |
| Tmin,Tmax | 0.784,0.939 | 0.701,0.940 |
| Tmin' | 0.503 | |

Correction method= # Reported T Limits: Tmin=0.701 Tmax=0.940
AbsCorr = ANALYTICAL

Data completeness= 0.991 Theta(max)= 72.128

R(reflections)= 0.0599( 5291) wR2(reflections)= 0.1518( 13018)

S = 0.782 Npar= 820

---

The following ALERTS were generated. Each ALERT has the format
**test-name_ALERT_alert-type_alert-level.**
Click on the hyperlinks for more details of the test.

---

**Alert level B**

PLAT230_ALERT_2_B Hirshfeld Test Diff for C30 --C35 . 8.3 s.u.

**Author Response: sample quality plus unusual bridging mode of the arene**

---

**Alert level C**

GOODF01_ALERT_2_C The least squares goodness of fit parameter lies
outside the range 0.80 <> 2.00
Goodness of fit given = 0.782
PLAT026_ALERT_3_C Ratio Observed / Unique Reflections (too) Low .. 41% Check
PLAT230_ALERT_2_C Hirshfeld Test Diff for C31 --C32 . 6.0 s.u.

**Author Response: sample quality plus unusual bridging mode of the arene**

```
PLAT234_ALERT_4_C Large Hirshfeld Difference C66      --C67      .       0.16 Ang.
PLAT242_ALERT_2_C Low    'MainMol' Ueq as Compared to Neighbors of        C11 Check
PLAT242_ALERT_2_C Low    'MainMol' Ueq as Compared to Neighbors of        C50 Check
PLAT245_ALERT_2_C U(iso) H12C      Smaller than U(eq) C12       by      0.019 Ang**2
PLAT711_ALERT_1_C BOND    Unknown or Inconsistent Label ..........          H Check
                    CA2         H
```

---

### Alert level G

```
PLAT002_ALERT_2_G Number of Distance or Angle Restraints on AtSite         15 Note
PLAT003_ALERT_2_G Number of Uiso or Uij Restrained non-H Atoms ...         20 Report
PLAT012_ALERT_1_G N.O.K.   _shelx_res_checksum Found in CIF ......     Please Check
PLAT013_ALERT_1_G N.O.K.   _shelx_hkl_checksum Found in CIF ......     Please Check
PLAT164_ALERT_4_G Nr. of Refined C-H H-Atoms in Heavy-Atom Struct.          6 Note
PLAT172_ALERT_4_G The CIF-Embedded .res File Contains DFIX Records          1 Report
PLAT176_ALERT_4_G The CIF-Embedded .res File Contains SADI Records          4 Report
PLAT178_ALERT_4_G The CIF-Embedded .res File Contains SIMU Records          2 Report
PLAT188_ALERT_3_G A Non-default SIMU Restraint Value has been used     0.0200 Report
PLAT188_ALERT_3_G A Non-default SIMU Restraint Value has been used     0.0200 Report
PLAT191_ALERT_3_G A Non-default SADI Restraint Value has been used     0.0050 Report
PLAT191_ALERT_3_G A Non-default SADI Restraint Value has been used     0.0050 Report
PLAT191_ALERT_3_G A Non-default SADI Restraint Value has been used     0.0050 Report
PLAT191_ALERT_3_G A Non-default SADI Restraint Value has been used     0.0100 Report
PLAT300_ALERT_4_G Atom Site Occupancy of C63A       Constrained at        0.6 Check
PLAT300_ALERT_4_G Atom Site Occupancy of C64A       Constrained at        0.6 Check
PLAT300_ALERT_4_G Atom Site Occupancy of C65A       Constrained at        0.6 Check
PLAT300_ALERT_4_G Atom Site Occupancy of C63        Constrained at        0.4 Check
PLAT300_ALERT_4_G Atom Site Occupancy of C64        Constrained at        0.4 Check
PLAT300_ALERT_4_G Atom Site Occupancy of C65        Constrained at        0.4 Check
PLAT300_ALERT_4_G Atom Site Occupancy of H63A       Constrained at        0.6 Check
PLAT300_ALERT_4_G Atom Site Occupancy of H64D       Constrained at        0.6 Check
PLAT300_ALERT_4_G Atom Site Occupancy of H64E       Constrained at        0.6 Check
PLAT300_ALERT_4_G Atom Site Occupancy of H64F       Constrained at        0.6 Check
PLAT300_ALERT_4_G Atom Site Occupancy of H65D       Constrained at        0.6 Check
PLAT300_ALERT_4_G Atom Site Occupancy of H65E       Constrained at        0.6 Check
PLAT300_ALERT_4_G Atom Site Occupancy of H65F       Constrained at        0.6 Check
PLAT300_ALERT_4_G Atom Site Occupancy of H63        Constrained at        0.4 Check
PLAT300_ALERT_4_G Atom Site Occupancy of H64A       Constrained at        0.4 Check
PLAT300_ALERT_4_G Atom Site Occupancy of H64B       Constrained at        0.4 Check
PLAT300_ALERT_4_G Atom Site Occupancy of H64C       Constrained at        0.4 Check
PLAT300_ALERT_4_G Atom Site Occupancy of H65A       Constrained at        0.4 Check
PLAT300_ALERT_4_G Atom Site Occupancy of H65B       Constrained at        0.4 Check
PLAT300_ALERT_4_G Atom Site Occupancy of H65C       Constrained at        0.4 Check
PLAT300_ALERT_4_G Atom Site Occupancy of C66        Constrained at        0.6 Check
PLAT300_ALERT_4_G Atom Site Occupancy of C67        Constrained at        0.6 Check
PLAT300_ALERT_4_G Atom Site Occupancy of C68        Constrained at        0.6 Check
PLAT300_ALERT_4_G Atom Site Occupancy of C69        Constrained at        0.6 Check
PLAT300_ALERT_4_G Atom Site Occupancy of C70        Constrained at        0.6 Check
PLAT300_ALERT_4_G Atom Site Occupancy of C71        Constrained at        0.6 Check
PLAT300_ALERT_4_G Atom Site Occupancy of C72        Constrained at        0.6 Check
PLAT300_ALERT_4_G Atom Site Occupancy of H66        Constrained at        0.6 Check
PLAT300_ALERT_4_G Atom Site Occupancy of H67        Constrained at        0.6 Check
PLAT300_ALERT_4_G Atom Site Occupancy of H68        Constrained at        0.6 Check
PLAT300_ALERT_4_G Atom Site Occupancy of H70        Constrained at        0.6 Check
PLAT300_ALERT_4_G Atom Site Occupancy of H71        Constrained at        0.6 Check
PLAT300_ALERT_4_G Atom Site Occupancy of H72A       Constrained at        0.6 Check
PLAT300_ALERT_4_G Atom Site Occupancy of H72B       Constrained at        0.6 Check
```

```
PLAT300_ALERT_4_G Atom Site Occupancy of H72C       Constrained at        0.6 Check
PLAT300_ALERT_4_G Atom Site Occupancy of C66A       Constrained at        0.4 Check
PLAT300_ALERT_4_G Atom Site Occupancy of C67A       Constrained at        0.4 Check
PLAT300_ALERT_4_G Atom Site Occupancy of C68A       Constrained at        0.4 Check
PLAT300_ALERT_4_G Atom Site Occupancy of C69A       Constrained at        0.4 Check
PLAT300_ALERT_4_G Atom Site Occupancy of C70A       Constrained at        0.4 Check
PLAT300_ALERT_4_G Atom Site Occupancy of C71A       Constrained at        0.4 Check
PLAT300_ALERT_4_G Atom Site Occupancy of C72A       Constrained at        0.4 Check
PLAT300_ALERT_4_G Atom Site Occupancy of H66A       Constrained at        0.4 Check
PLAT300_ALERT_4_G Atom Site Occupancy of H67A       Constrained at        0.4 Check
PLAT300_ALERT_4_G Atom Site Occupancy of H68A       Constrained at        0.4 Check
PLAT300_ALERT_4_G Atom Site Occupancy of H70A       Constrained at        0.4 Check
PLAT300_ALERT_4_G Atom Site Occupancy of H71A       Constrained at        0.4 Check
PLAT300_ALERT_4_G Atom Site Occupancy of H72D       Constrained at        0.4 Check
PLAT300_ALERT_4_G Atom Site Occupancy of H72E       Constrained at        0.4 Check
PLAT300_ALERT_4_G Atom Site Occupancy of H72F       Constrained at        0.4 Check
PLAT301_ALERT_3_G Main Residue  Disorder .............(Resd  1  )          4% Note
PLAT302_ALERT_4_G Anion/Solvent/Minor-Residue Disorder (Resd  2  )       100% Note
PLAT302_ALERT_4_G Anion/Solvent/Minor-Residue Disorder (Resd  3  )       100% Note
PLAT303_ALERT_2_G Full Occupancy Atom H1        with # Connections       2.00 Check
PLAT343_ALERT_2_G Unusual sp2      Angle Range in Main Residue for        C30 Check
PLAT380_ALERT_4_G Incorrectly? Oriented X(sp2)-Methyl Moiety .....        C72 Check
PLAT790_ALERT_4_G Centre of Gravity not Within Unit Cell: Resd.  #          2 Note
              C7 H8
PLAT790_ALERT_4_G Centre of Gravity not Within Unit Cell: Resd.  #          3 Note
              C7 H8
PLAT860_ALERT_3_G Number of Least-Squares Restraints ............         300 Note
PLAT870_ALERT_4_G ALERTS Related to Twinning Effects Suppressed ..          ! Info
PLAT933_ALERT_2_G Number of HKL-OMIT Records in Embedded .res File          6 Note
PLAT941_ALERT_3_G Average HKL Measurement Multiplicity ..........         1.0 Low
```

---

   0 **ALERT level A** = Most likely a serious problem - resolve or explain
   1 **ALERT level B** = A potentially serious problem, consider carefully
   8 **ALERT level C** = Check. Ensure it is not caused by an omission or oversight
  76 **ALERT level G** = General information/check it is not something unexpected

   3 ALERT type 1 CIF construction/syntax error, inconsistent or missing data
  11 ALERT type 2 Indicator that the structure model may be wrong or deficient
  10 ALERT type 3 Indicator that the structure quality may be low
  61 ALERT type 4 Improvement, methodology, query or suggestion
   0 ALERT type 5 Informative message, check

---

# Datablock: e21msh25-cpd-10

---

| | | | |
|---|---|---|---|
| Bond precision: | C-C = 0.0022 A | | Wavelength=0.71073 |
| Cell: | a=14.7119(1) | b=22.1897(2) | c=23.3651(3) |
| | alpha=90 | beta=99.106(1) | gamma=90 |
| Temperature: | 150 K | | |

| | Calculated | Reported |
|---|---|---|
| Volume | 7531.47(13) | 7531.47(13) |
| Space group | P 21/n | P 1 21/n 1 |
| Hall group | -P 2yn | -P 2yn |
| Moiety formula | C72 H104 Ca2 N4, 1.5(C6 H6) | C72 H104 Ca2 N4, 1.5(C6 H6) |
| Sum formula | C81 H113 Ca2 N4 | C81 H113 Ca2 N4 |
| Mr | 1222.92 | 1222.91 |
| Dx,g cm-3 | 1.079 | 1.079 |
| Z | 4 | 4 |
| Mu (mm-1) | 0.194 | 0.194 |
| F000 | 2668.0 | 2668.0 |
| F000’ | 2670.61 | |
| h,k,lmax | 20,31,33 | 20,31,33 |
| Nref | 22778 | 20379 |
| Tmin,Tmax | 0.910,0.949 | 0.932,1.000 |
| Tmin’ | 0.878 | |

Correction method= # Reported T Limits: Tmin=0.932 Tmax=1.000
AbsCorr = MULTI-SCAN

Data completeness= 0.895 Theta(max)= 30.402

R(reflections)= 0.0443( 15938) wR2(reflections)= 0.1210( 20379)

S = 1.030 Npar= 856

---

The following ALERTS were generated. Each ALERT has the format
**test-name_ALERT_alert-type_alert-level.**
Click on the hyperlinks for more details of the test.

---

**Alert level C**

```
PLAT220_ALERT_2_C NonSolvent   Resd 1  C   Ueq(max)/Ueq(min) Range       4.4 Ratio
PLAT222_ALERT_3_C NonSolvent Resd 1  H   Uiso(max)/Uiso(min) Range       4.4 Ratio
PLAT242_ALERT_2_C Low    'MainMol' Ueq as Compared to Neighbors of       C12 Check
PLAT242_ALERT_2_C Low    'MainMol' Ueq as Compared to Neighbors of       C27 Check
PLAT331_ALERT_2_C Small Aver Phenyl C-C Dist C79      --C81_a    .      1.36 Ang.
```

---

**Alert level G**

```
PLAT012_ALERT_1_G No      _shelx_res_checksum Found in CIF ......     Please Check
PLAT063_ALERT_4_G Crystal Size Possibly too Large for Beam Size ..       0.67 mm
PLAT142_ALERT_4_G s.u. on b - Axis Small or Missing .............    0.00020 Ang.
PLAT164_ALERT_4_G Nr. of Refined C-H H-Atoms in Heavy-Atom Struct.          3 Note
PLAT300_ALERT_4_G Atom Site Occupancy of C73        Constrained at       0.65 Check
PLAT300_ALERT_4_G Atom Site Occupancy of C74        Constrained at       0.65 Check
PLAT300_ALERT_4_G Atom Site Occupancy of C75        Constrained at       0.65 Check
PLAT300_ALERT_4_G Atom Site Occupancy of C76        Constrained at       0.65 Check
PLAT300_ALERT_4_G Atom Site Occupancy of C77        Constrained at       0.65 Check
```

```
PLAT300_ALERT_4_G Atom Site Occupancy of C78        Constrained at       0.65 Check
PLAT300_ALERT_4_G Atom Site Occupancy of H73        Constrained at       0.65 Check
PLAT300_ALERT_4_G Atom Site Occupancy of H74        Constrained at       0.65 Check
PLAT300_ALERT_4_G Atom Site Occupancy of H75        Constrained at       0.65 Check
PLAT300_ALERT_4_G Atom Site Occupancy of H76        Constrained at       0.65 Check
PLAT300_ALERT_4_G Atom Site Occupancy of H77        Constrained at       0.65 Check
PLAT300_ALERT_4_G Atom Site Occupancy of H78        Constrained at       0.65 Check
PLAT300_ALERT_4_G Atom Site Occupancy of C73A       Constrained at       0.35 Check
PLAT300_ALERT_4_G Atom Site Occupancy of C74A       Constrained at       0.35 Check
PLAT300_ALERT_4_G Atom Site Occupancy of C75A       Constrained at       0.35 Check
PLAT300_ALERT_4_G Atom Site Occupancy of C76A       Constrained at       0.35 Check
PLAT300_ALERT_4_G Atom Site Occupancy of C77A       Constrained at       0.35 Check
PLAT300_ALERT_4_G Atom Site Occupancy of C78A       Constrained at       0.35 Check
PLAT300_ALERT_4_G Atom Site Occupancy of H73A       Constrained at       0.35 Check
PLAT300_ALERT_4_G Atom Site Occupancy of H74A       Constrained at       0.35 Check
PLAT300_ALERT_4_G Atom Site Occupancy of H75A       Constrained at       0.35 Check
PLAT300_ALERT_4_G Atom Site Occupancy of H76A       Constrained at       0.35 Check
PLAT300_ALERT_4_G Atom Site Occupancy of H77A       Constrained at       0.35 Check
PLAT300_ALERT_4_G Atom Site Occupancy of H78A       Constrained at       0.35 Check
PLAT302_ALERT_4_G Anion/Solvent/Minor-Residue Disorder (Resd  2  )       100% Note
PLAT302_ALERT_4_G Anion/Solvent/Minor-Residue Disorder (Resd  4  )       100% Note
PLAT303_ALERT_2_G Full Occupancy Atom H1        with # Connections       2.00 Check
PLAT304_ALERT_4_G Non-Integer Number of Atoms in ..... (Resd  2  )       7.80 Check
PLAT304_ALERT_4_G Non-Integer Number of Atoms in ..... (Resd  4  )       4.20 Check
PLAT343_ALERT_2_G Unusual sp?      Angle Range in Main Residue for        C59 Check
PLAT779_ALERT_4_G Suspect or Irrelevant (Bond) Angle(s) in CIF ...      41.67 Deg.
              N1   -C6   -CA1     1_555   1_555   1_555 ......     #   62 Check
PLAT779_ALERT_4_G Suspect or Irrelevant (Bond) Angle(s) in CIF ...      40.17 Deg.
              N2   -C18  -CA1     1_555   1_555   1_555 ......     #   83 Check
PLAT779_ALERT_4_G Suspect or Irrelevant (Bond) Angle(s) in CIF ...      42.93 Deg.
              N4   -C35  -CA2     1_555   1_555   1_555 ......     #  111 Check
PLAT779_ALERT_4_G Suspect or Irrelevant (Bond) Angle(s) in CIF ...      39.89 Deg.
              N3   -C47  -CA2     1_555   1_555   1_555 ......     #  132 Check
PLAT941_ALERT_3_G Average HKL Measurement Multiplicity ..........         4.1 Low
```

---

```
   0 ALERT level A = Most likely a serious problem - resolve or explain
   0 ALERT level B = A potentially serious problem, consider carefully
   5 ALERT level C = Check. Ensure it is not caused by an omission or oversight
  39 ALERT level G = General information/check it is not something unexpected

   1 ALERT type 1 CIF construction/syntax error, inconsistent or missing data
   6 ALERT type 2 Indicator that the structure model may be wrong or deficient
   2 ALERT type 3 Indicator that the structure quality may be low
  35 ALERT type 4 Improvement, methodology, query or suggestion
   0 ALERT type 5 Informative message, check
```

---

# Datablock: s21msh59-cpd-11

---

```
Bond precision:   C-C = 0.0020 A          Wavelength=1.54184
```

| | | | |
|---|---|---|---|
| Cell: | a=13.2053(1) | b=13.7705(2) | c=23.6922(3) |
| | alpha=95.630(1) | beta=91.556(1) | gamma=93.176(1) |
| Temperature: | 150 K | | |

| | Calculated | Reported |
|---|---|---|
| Volume | 4278.60(9) | 4278.60(9) |
| Space group | P -1 | P -1 |
| Hall group | -P 1 | -P 1 |
| Moiety formula | C78 H108 Ca2 N4, 3(C6 H6) | C78 H108 Ca2 N4, 3(C6 H6) |
| Sum formula | C96 H126 Ca2 N4 | C96 H126 Ca2 N4 |
| Mr | 1416.17 | 1416.16 |
| Dx,g cm-3 | 1.099 | 1.099 |
| Z | 2 | 2 |
| Mu (mm-1) | 1.496 | 1.496 |
| F000 | 1540.0 | 1540.0 |
| F000' | 1544.99 | |
| h,k,lmax | 16,17,29 | 16,17,29 |
| Nref | 17245 | 17101 |
| Tmin,Tmax | 0.596,0.757 | 0.829,1.000 |
| Tmin' | 0.507 | |

Correction method= # Reported T Limits: Tmin=0.829 Tmax=1.000
AbsCorr = MULTI-SCAN

Data completeness= 0.992 Theta(max)= 73.520

R(reflections)= 0.0375( 16498) wR2(reflections)= 0.1028( 17101)

S = 1.027 Npar= 1052

---

The following ALERTS were generated. Each ALERT has the format
**test-name_ALERT_alert-type_alert-level.**
Click on the hyperlinks for more details of the test.

---

**Alert level C**

```
PLAT220_ALERT_2_C NonSolvent   Resd 1  C   Ueq(max)/Ueq(min) Range        4.0 Ratio
PLAT241_ALERT_2_C High   'MainMol' Ueq as Compared to Neighbors of        C33 Check
PLAT250_ALERT_2_C Large U3/U1 Ratio for Average U(i,j) Tensor ....        2.1 Note
```

---

**Alert level G**

```
PLAT003_ALERT_2_G Number of Uiso or Uij Restrained non-H Atoms ...         36 Report
PLAT012_ALERT_1_G N.O.K.   _shelx_res_checksum Found in CIF ......     Please Check
PLAT013_ALERT_1_G N.O.K.   _shelx_hkl_checksum Found in CIF ......     Please Check
PLAT154_ALERT_1_G The s.u.'s on the Cell Angles are Equal ..(Note)      0.001 Degree
PLAT164_ALERT_4_G Nr. of Refined C-H H-Atoms in Heavy-Atom Struct.          5 Note
```

```
PLAT178_ALERT_4_G The CIF-Embedded .res File Contains SIMU Records          3 Report
PLAT188_ALERT_3_G A Non-default SIMU Restraint Value has been used     0.0200 Report
PLAT188_ALERT_3_G A Non-default SIMU Restraint Value has been used     0.0200 Report
PLAT188_ALERT_3_G A Non-default SIMU Restraint Value has been used     0.0200 Report
PLAT300_ALERT_4_G Atom Site Occupancy of C79        Constrained at        0.5 Check
PLAT300_ALERT_4_G Atom Site Occupancy of C80        Constrained at        0.5 Check
PLAT300_ALERT_4_G Atom Site Occupancy of C81        Constrained at        0.5 Check
PLAT300_ALERT_4_G Atom Site Occupancy of C82        Constrained at        0.5 Check
PLAT300_ALERT_4_G Atom Site Occupancy of C83        Constrained at        0.5 Check
PLAT300_ALERT_4_G Atom Site Occupancy of C84        Constrained at        0.5 Check
PLAT300_ALERT_4_G Atom Site Occupancy of H79        Constrained at        0.5 Check
PLAT300_ALERT_4_G Atom Site Occupancy of H80        Constrained at        0.5 Check
PLAT300_ALERT_4_G Atom Site Occupancy of H81        Constrained at        0.5 Check
PLAT300_ALERT_4_G Atom Site Occupancy of H82        Constrained at        0.5 Check
PLAT300_ALERT_4_G Atom Site Occupancy of H83        Constrained at        0.5 Check
PLAT300_ALERT_4_G Atom Site Occupancy of H84        Constrained at        0.5 Check
PLAT300_ALERT_4_G Atom Site Occupancy of C79A       Constrained at        0.5 Check
PLAT300_ALERT_4_G Atom Site Occupancy of C80A       Constrained at        0.5 Check
PLAT300_ALERT_4_G Atom Site Occupancy of C81A       Constrained at        0.5 Check
PLAT300_ALERT_4_G Atom Site Occupancy of C82A       Constrained at        0.5 Check
PLAT300_ALERT_4_G Atom Site Occupancy of C83A       Constrained at        0.5 Check
PLAT300_ALERT_4_G Atom Site Occupancy of C84A       Constrained at        0.5 Check
PLAT300_ALERT_4_G Atom Site Occupancy of H79A       Constrained at        0.5 Check
PLAT300_ALERT_4_G Atom Site Occupancy of H80A       Constrained at        0.5 Check
PLAT300_ALERT_4_G Atom Site Occupancy of H81A       Constrained at        0.5 Check
PLAT300_ALERT_4_G Atom Site Occupancy of H82A       Constrained at        0.5 Check
PLAT300_ALERT_4_G Atom Site Occupancy of H83A       Constrained at        0.5 Check
PLAT300_ALERT_4_G Atom Site Occupancy of H84A       Constrained at        0.5 Check
PLAT300_ALERT_4_G Atom Site Occupancy of C85        Constrained at       0.55 Check
PLAT300_ALERT_4_G Atom Site Occupancy of C86        Constrained at       0.55 Check
PLAT300_ALERT_4_G Atom Site Occupancy of C87        Constrained at       0.55 Check
PLAT300_ALERT_4_G Atom Site Occupancy of C88        Constrained at       0.55 Check
PLAT300_ALERT_4_G Atom Site Occupancy of C89        Constrained at       0.55 Check
PLAT300_ALERT_4_G Atom Site Occupancy of C90        Constrained at       0.55 Check
PLAT300_ALERT_4_G Atom Site Occupancy of H85        Constrained at       0.55 Check
PLAT300_ALERT_4_G Atom Site Occupancy of H86        Constrained at       0.55 Check
PLAT300_ALERT_4_G Atom Site Occupancy of H87        Constrained at       0.55 Check
PLAT300_ALERT_4_G Atom Site Occupancy of H88        Constrained at       0.55 Check
PLAT300_ALERT_4_G Atom Site Occupancy of H89        Constrained at       0.55 Check
PLAT300_ALERT_4_G Atom Site Occupancy of H90        Constrained at       0.55 Check
PLAT300_ALERT_4_G Atom Site Occupancy of C91        Constrained at        0.5 Check
PLAT300_ALERT_4_G Atom Site Occupancy of C92        Constrained at        0.5 Check
PLAT300_ALERT_4_G Atom Site Occupancy of C93        Constrained at        0.5 Check
PLAT300_ALERT_4_G Atom Site Occupancy of C94        Constrained at        0.5 Check
PLAT300_ALERT_4_G Atom Site Occupancy of C95        Constrained at        0.5 Check
PLAT300_ALERT_4_G Atom Site Occupancy of C96        Constrained at        0.5 Check
PLAT300_ALERT_4_G Atom Site Occupancy of H91        Constrained at        0.5 Check
PLAT300_ALERT_4_G Atom Site Occupancy of H92        Constrained at        0.5 Check
PLAT300_ALERT_4_G Atom Site Occupancy of H93        Constrained at        0.5 Check
PLAT300_ALERT_4_G Atom Site Occupancy of H94        Constrained at        0.5 Check
PLAT300_ALERT_4_G Atom Site Occupancy of H95        Constrained at        0.5 Check
PLAT300_ALERT_4_G Atom Site Occupancy of H96        Constrained at        0.5 Check
PLAT300_ALERT_4_G Atom Site Occupancy of C91A       Constrained at        0.5 Check
PLAT300_ALERT_4_G Atom Site Occupancy of C92A       Constrained at        0.5 Check
PLAT300_ALERT_4_G Atom Site Occupancy of C93A       Constrained at        0.5 Check
PLAT300_ALERT_4_G Atom Site Occupancy of C94A       Constrained at        0.5 Check
PLAT300_ALERT_4_G Atom Site Occupancy of C95A       Constrained at        0.5 Check
```

```
PLAT300_ALERT_4_G Atom Site Occupancy of C96A       Constrained at        0.5 Check
PLAT300_ALERT_4_G Atom Site Occupancy of H91A       Constrained at        0.5 Check
PLAT300_ALERT_4_G Atom Site Occupancy of H92A       Constrained at        0.5 Check
PLAT300_ALERT_4_G Atom Site Occupancy of H93A       Constrained at        0.5 Check
PLAT300_ALERT_4_G Atom Site Occupancy of H94A       Constrained at        0.5 Check
PLAT300_ALERT_4_G Atom Site Occupancy of H95A       Constrained at        0.5 Check
PLAT300_ALERT_4_G Atom Site Occupancy of H96A       Constrained at        0.5 Check
PLAT300_ALERT_4_G Atom Site Occupancy of C85A       Constrained at       0.45 Check
PLAT300_ALERT_4_G Atom Site Occupancy of C86A       Constrained at       0.45 Check
PLAT300_ALERT_4_G Atom Site Occupancy of C87A       Constrained at       0.45 Check
PLAT300_ALERT_4_G Atom Site Occupancy of C88A       Constrained at       0.45 Check
PLAT300_ALERT_4_G Atom Site Occupancy of C89A       Constrained at       0.45 Check
PLAT300_ALERT_4_G Atom Site Occupancy of C90A       Constrained at       0.45 Check
PLAT300_ALERT_4_G Atom Site Occupancy of H85A       Constrained at       0.45 Check
PLAT300_ALERT_4_G Atom Site Occupancy of H86A       Constrained at       0.45 Check
PLAT300_ALERT_4_G Atom Site Occupancy of H87A       Constrained at       0.45 Check
PLAT300_ALERT_4_G Atom Site Occupancy of H88A       Constrained at       0.45 Check
PLAT300_ALERT_4_G Atom Site Occupancy of H89A       Constrained at       0.45 Check
PLAT300_ALERT_4_G Atom Site Occupancy of H90A       Constrained at       0.45 Check
PLAT302_ALERT_4_G Anion/Solvent/Minor-Residue Disorder (Resd  2  )       100% Note
PLAT302_ALERT_4_G Anion/Solvent/Minor-Residue Disorder (Resd  3  )       100% Note
PLAT302_ALERT_4_G Anion/Solvent/Minor-Residue Disorder (Resd  4  )       100% Note
PLAT302_ALERT_4_G Anion/Solvent/Minor-Residue Disorder (Resd  5  )       100% Note
PLAT302_ALERT_4_G Anion/Solvent/Minor-Residue Disorder (Resd  6  )       100% Note
PLAT302_ALERT_4_G Anion/Solvent/Minor-Residue Disorder (Resd  7  )       100% Note
PLAT304_ALERT_4_G Non-Integer Number of Atoms in ..... (Resd  4  )       6.60 Check
PLAT304_ALERT_4_G Non-Integer Number of Atoms in ..... (Resd  7  )       5.40 Check
PLAT343_ALERT_2_G Unusual sp?      Angle Range in Main Residue for        C36 Check
PLAT860_ALERT_3_G Number of Least-Squares Restraints ............         546 Note
PLAT941_ALERT_3_G Average HKL Measurement Multiplicity ..........         3.5 Low
```

---

   0 **ALERT level A** = Most likely a serious problem - resolve or explain
   0 **ALERT level B** = A potentially serious problem, consider carefully
   3 **ALERT level C** = Check. Ensure it is not caused by an omission or oversight
  92 **ALERT level G** = General information/check it is not something unexpected

   3 ALERT type 1 CIF construction/syntax error, inconsistent or missing data
   5 ALERT type 2 Indicator that the structure model may be wrong or deficient
   5 ALERT type 3 Indicator that the structure quality may be low
  82 ALERT type 4 Improvement, methodology, query or suggestion
   0 ALERT type 5 Informative message, check

---

# Datablock: s21msh68-cpd-12

---

Bond precision: C-C = 0.0019 A Wavelength=1.54184

| | | | |
|---|---|---|---|
| Cell: | a=12.7553(2) | b=13.2827(2) | c=26.9923(5) |
| | alpha=80.009(2) | beta=81.863(2) | gamma=64.624(2) |
| Temperature: | 150 K | | |

| | Calculated | Reported |
|---|---|---|
| Volume | 4057.65(14) | 4057.65(13) |
| Space group | P -1 | P -1 |
| Hall group | -P 1 | -P 1 |
| Moiety formula | C79 H110 Ca2 N4 [+ solvent] | C79 H110 Ca2 N4 |
| Sum formula | C79 H110 Ca2 N4 [+ solvent] | C88 H119 Ca2 N4 |
| Mr | 1195.88 | 1313.02 |
| Dx,g cm-3 | 0.979 | 1.075 |
| Z | 2 | 2 |
| Mu (mm-1) | 1.502 | 1.542 |
| F000 | 1304.0 | 1430.0 |
| F000' | 1308.40 | |
| h,k,lmax | 15,16,33 | 15,16,33 |
| Nref | 16231 | 16100 |
| Tmin,Tmax | 0.778,0.853 | 0.987,1.000 |
| Tmin' | 0.709 | |

Correction method= # Reported T Limits: Tmin=0.987 Tmax=1.000
AbsCorr = MULTI-SCAN

Data completeness= 0.992 Theta(max)= 73.038

R(reflections)= 0.0347( 14864) wR2(reflections)= 0.0961( 16100)

S = 1.045 Npar= 835

---

The following ALERTS were generated. Each ALERT has the format
**test-name_ALERT_alert-type_alert-level.**
Click on the hyperlinks for more details of the test.

---

## Alert level C

```
PLAT220_ALERT_2_C NonSolvent   Resd 1  C   Ueq(max)/Ueq(min) Range        3.4 Ratio
PLAT222_ALERT_3_C NonSolvent Resd 1  H   Uiso(max)/Uiso(min) Range        4.3 Ratio
PLAT242_ALERT_2_C Low    'MainMol' Ueq as Compared to Neighbors of        C24 Check
PLAT242_ALERT_2_C Low    'MainMol' Ueq as Compared to Neighbors of        C36 Check
PLAT242_ALERT_2_C Low    'MainMol' Ueq as Compared to Neighbors of        C65 Check
```

---

## Alert level G

```
FORMU01_ALERT_1_G  There is a discrepancy between the atom counts in the
            _chemical_formula_sum and _chemical_formula_moiety. This is
            usually due to the moiety formula being in the wrong format.
            Atom count from _chemical_formula_sum:   C88 H119 Ca2 N4
            Atom count from _chemical_formula_moiety:C79 H110 Ca2 N4
FORMU01_ALERT_2_G  There is a discrepancy between the atom counts in the
            _chemical_formula_sum and the formula from the _atom_site* data.
            Atom count from _chemical_formula_sum:C88 H119 Ca2 N4
            Atom count from the _atom_site data:  C79 H110 Ca2 N4
```

```
CELLZ01_ALERT_1_G Difference between formula and atom_site contents detected.
CELLZ01_ALERT_1_G ALERT: Large difference may be due to a
            symmetry error - see SYMMG tests
           From the CIF: _cell_formula_units_Z    2
           From the CIF: _chemical_formula_sum  C88 H119 Ca2 N4
           TEST: Compare cell contents of formula and atom_site data

           atom    Z*formula  cif sites diff
           C        176.00    158.00   18.00
           H        238.00    220.00   18.00
           Ca         4.00      4.00    0.00
           N          8.00      8.00    0.00
PLAT012_ALERT_1_G N.O.K.   _shelx_res_checksum Found in CIF ......     Please Check
PLAT041_ALERT_1_G Calc. and Reported SumFormula    Strings  Differ     Please Check
PLAT051_ALERT_1_G Mu(calc) and Mu(CIF) Ratio Differs from 1.0 by .       2.62 %
PLAT154_ALERT_1_G The s.u.'s on the Cell Angles are Equal ..(Note)      0.002 Degree
PLAT164_ALERT_4_G Nr. of Refined C-H H-Atoms in Heavy-Atom Struct.          3 Note
PLAT300_ALERT_4_G Atom Site Occupancy of C37       Constrained at       0.55 Check
PLAT300_ALERT_4_G Atom Site Occupancy of C38       Constrained at       0.55 Check
PLAT300_ALERT_4_G Atom Site Occupancy of C39       Constrained at       0.55 Check
PLAT300_ALERT_4_G Atom Site Occupancy of C37A      Constrained at       0.45 Check
PLAT300_ALERT_4_G Atom Site Occupancy of C38A      Constrained at       0.45 Check
PLAT300_ALERT_4_G Atom Site Occupancy of C39A      Constrained at       0.45 Check
PLAT300_ALERT_4_G Atom Site Occupancy of H37A      Constrained at       0.55 Check
PLAT300_ALERT_4_G Atom Site Occupancy of H37B      Constrained at       0.55 Check
PLAT300_ALERT_4_G Atom Site Occupancy of H37C      Constrained at       0.55 Check
PLAT300_ALERT_4_G Atom Site Occupancy of H38A      Constrained at       0.55 Check
PLAT300_ALERT_4_G Atom Site Occupancy of H38B      Constrained at       0.55 Check
PLAT300_ALERT_4_G Atom Site Occupancy of H38C      Constrained at       0.55 Check
PLAT300_ALERT_4_G Atom Site Occupancy of H39A      Constrained at       0.55 Check
PLAT300_ALERT_4_G Atom Site Occupancy of H39B      Constrained at       0.55 Check
PLAT300_ALERT_4_G Atom Site Occupancy of H39C      Constrained at       0.55 Check
PLAT300_ALERT_4_G Atom Site Occupancy of H37D      Constrained at       0.45 Check
PLAT300_ALERT_4_G Atom Site Occupancy of H37E      Constrained at       0.45 Check
PLAT300_ALERT_4_G Atom Site Occupancy of H37F      Constrained at       0.45 Check
PLAT300_ALERT_4_G Atom Site Occupancy of H38D      Constrained at       0.45 Check
PLAT300_ALERT_4_G Atom Site Occupancy of H38E      Constrained at       0.45 Check
PLAT300_ALERT_4_G Atom Site Occupancy of H38F      Constrained at       0.45 Check
PLAT300_ALERT_4_G Atom Site Occupancy of H39D      Constrained at       0.45 Check
PLAT300_ALERT_4_G Atom Site Occupancy of H39E      Constrained at       0.45 Check
PLAT300_ALERT_4_G Atom Site Occupancy of H39F      Constrained at       0.45 Check
PLAT301_ALERT_3_G Main Residue  Disorder .............(Resd  1  )          4% Note
PLAT343_ALERT_2_G Unusual          Angle Range in Main Residue for        C30 Check
PLAT343_ALERT_2_G Unusual sp?      Angle Range in Main Residue for        C44 Check
PLAT606_ALERT_4_G Solvent Accessible VOID(S) in Structure ........          ! Info
PLAT802_ALERT_4_G CIF Input Record(s) with more than 80 Characters          2 Info
PLAT868_ALERT_4_G ALERTS Due to the Use of _smtbx_masks Suppressed          ! Info
PLAT933_ALERT_2_G Number of HKL-OMIT Records in Embedded .res File          1 Note
PLAT941_ALERT_3_G Average HKL Measurement Multiplicity ..........         3.8 Low
```

---

0 **ALERT level A** = Most likely a serious problem - resolve or explain
0 **ALERT level B** = A potentially serious problem, consider carefully
5 **ALERT level C** = Check. Ensure it is not caused by an omission or oversight
41 **ALERT level G** = General information/check it is not something unexpected

7 ALERT type 1 CIF construction/syntax error, inconsistent or missing data

```
  8 ALERT type 2 Indicator that the structure model may be wrong or deficient
  3 ALERT type 3 Indicator that the structure quality may be low
 28 ALERT type 4 Improvement, methodology, query or suggestion
  0 ALERT type 5 Informative message, check
```

---

## Datablock: s22msh86-cpd-14

---

Bond precision: C-C = 0.0066 A Wavelength=1.54184

| | | | |
|---|---|---|---|
| Cell: | a=16.0402(5) | b=16.2877(6) | c=24.6017(7) |
| | alpha=90 | beta=95.011(3) | gamma=90 |
| Temperature: | 150 K | | |

| | Calculated | Reported |
|---|---|---|
| Volume | 6402.8(4) | 6402.8(4) |
| Space group | P 21/c | P 1 21/c 1 |
| Hall group | -P 2ybc | -P 2ybc |
| Moiety formula | C128 H174 Ca4 Cl2 N8, 2(C6 H6) | C128 H174 Ca4 Cl2 N8, 2(C6 H6) |
| Sum formula | C140 H186 Ca4 Cl2 N8 | C140 H186 Ca4 Cl2 N8 |
| Mr | 2212.19 | 2212.18 |
| Dx,g cm-3 | 1.148 | 1.147 |
| Z | 2 | 2 |
| Mu (mm-1) | 2.242 | 2.242 |
| F000 | 2392.0 | 2392.0 |
| F000’ | 2401.66 | |
| h,k,lmax | 19,20,30 | 19,20,30 |
| Nref | 12922 | 12759 |
| Tmin,Tmax | 0.786,0.922 | 0.761,1.000 |
| Tmin’ | 0.713 | |

Correction method= # Reported T Limits: Tmin=0.761 Tmax=1.000
AbsCorr = MULTI-SCAN

Data completeness= 0.987 Theta(max)= 73.603

R(reflections)= 0.0767( 8372) wR2(reflections)= 0.2322( 12759)

S = 1.021 Npar= 764

---

```
The following ALERTS were generated. Each ALERT has the format
       test-name_ALERT_alert-type_alert-level.
Click on the hyperlinks for more details of the test.
```

---

### Alert level B

```
PLAT230_ALERT_2_B Hirshfeld Test Diff for    C33      --C34      .       12.3 s.u.
```

**Author Response: sample quality plus unusual bridging mode of the arene**

---

### Alert level C

```
PLAT230_ALERT_2_C Hirshfeld Test Diff for    C32      --C33      .        6.0 s.u.
```

**Author Response: sample quality plus unusual bridging mode of the arene**

```
PLAT242_ALERT_2_C Low    'MainMol' Ueq as Compared to Neighbors of        C59 Check
PLAT250_ALERT_2_C Large U3/U1 Ratio for Average U(i,j) Tensor ....        2.2 Note
PLAT250_ALERT_2_C Large U3/U1 Ratio for Average U(i,j) Tensor ....        2.8 Note
PLAT341_ALERT_3_C Low Bond Precision on  C-C Bonds ...............    0.00664 Ang.
```

---

### Alert level G

```
PLAT002_ALERT_2_G Number of Distance or Angle Restraints on AtSite          5 Note
PLAT003_ALERT_2_G Number of Uiso or Uij Restrained non-H Atoms ...         15 Report
PLAT083_ALERT_2_G SHELXL Second Parameter in WGHT  Unusually Large       6.22 Why ?
PLAT176_ALERT_4_G The CIF-Embedded .res File Contains SADI Records          2 Report
PLAT178_ALERT_4_G The CIF-Embedded .res File Contains SIMU Records          2 Report
PLAT188_ALERT_3_G A Non-default SIMU Restraint Value has been used     0.0200 Report
PLAT188_ALERT_3_G A Non-default SIMU Restraint Value has been used     0.0200 Report
PLAT191_ALERT_3_G A Non-default SADI Restraint Value has been used     0.0050 Report
PLAT191_ALERT_3_G A Non-default SADI Restraint Value has been used     0.0100 Report
PLAT300_ALERT_4_G Atom Site Occupancy of C60        Constrained at       0.57 Check
PLAT300_ALERT_4_G Atom Site Occupancy of C61        Constrained at       0.57 Check
PLAT300_ALERT_4_G Atom Site Occupancy of C60A       Constrained at       0.43 Check
PLAT300_ALERT_4_G Atom Site Occupancy of C61A       Constrained at       0.43 Check
PLAT300_ALERT_4_G Atom Site Occupancy of H59        Constrained at       0.57 Check
PLAT300_ALERT_4_G Atom Site Occupancy of H60A       Constrained at       0.57 Check
PLAT300_ALERT_4_G Atom Site Occupancy of H60B       Constrained at       0.57 Check
PLAT300_ALERT_4_G Atom Site Occupancy of H60C       Constrained at       0.57 Check
PLAT300_ALERT_4_G Atom Site Occupancy of H61A       Constrained at       0.57 Check
PLAT300_ALERT_4_G Atom Site Occupancy of H61B       Constrained at       0.57 Check
PLAT300_ALERT_4_G Atom Site Occupancy of H61C       Constrained at       0.57 Check
PLAT300_ALERT_4_G Atom Site Occupancy of H59A       Constrained at       0.43 Check
PLAT300_ALERT_4_G Atom Site Occupancy of H60D       Constrained at       0.43 Check
PLAT300_ALERT_4_G Atom Site Occupancy of H60E       Constrained at       0.43 Check
PLAT300_ALERT_4_G Atom Site Occupancy of H60F       Constrained at       0.43 Check
PLAT300_ALERT_4_G Atom Site Occupancy of H61D       Constrained at       0.43 Check
PLAT300_ALERT_4_G Atom Site Occupancy of H61E       Constrained at       0.43 Check
PLAT300_ALERT_4_G Atom Site Occupancy of H61F       Constrained at       0.43 Check
PLAT300_ALERT_4_G Atom Site Occupancy of C65        Constrained at        0.5 Check
PLAT300_ALERT_4_G Atom Site Occupancy of C66        Constrained at        0.5 Check
PLAT300_ALERT_4_G Atom Site Occupancy of C67        Constrained at        0.5 Check
```

```
PLAT300_ALERT_4_G Atom Site Occupancy of C68        Constrained at        0.5 Check
PLAT300_ALERT_4_G Atom Site Occupancy of C69        Constrained at        0.5 Check
PLAT300_ALERT_4_G Atom Site Occupancy of C70        Constrained at        0.5 Check
PLAT300_ALERT_4_G Atom Site Occupancy of H65        Constrained at        0.5 Check
PLAT300_ALERT_4_G Atom Site Occupancy of H66        Constrained at        0.5 Check
PLAT300_ALERT_4_G Atom Site Occupancy of H67        Constrained at        0.5 Check
PLAT300_ALERT_4_G Atom Site Occupancy of H68        Constrained at        0.5 Check
PLAT300_ALERT_4_G Atom Site Occupancy of H69        Constrained at        0.5 Check
PLAT300_ALERT_4_G Atom Site Occupancy of H70        Constrained at        0.5 Check
PLAT300_ALERT_4_G Atom Site Occupancy of C65A       Constrained at        0.5 Check
PLAT300_ALERT_4_G Atom Site Occupancy of C66A       Constrained at        0.5 Check
PLAT300_ALERT_4_G Atom Site Occupancy of C67A       Constrained at        0.5 Check
PLAT300_ALERT_4_G Atom Site Occupancy of C68A       Constrained at        0.5 Check
PLAT300_ALERT_4_G Atom Site Occupancy of C69A       Constrained at        0.5 Check
PLAT300_ALERT_4_G Atom Site Occupancy of C70A       Constrained at        0.5 Check
PLAT300_ALERT_4_G Atom Site Occupancy of H65A       Constrained at        0.5 Check
PLAT300_ALERT_4_G Atom Site Occupancy of H66A       Constrained at        0.5 Check
PLAT300_ALERT_4_G Atom Site Occupancy of H67A       Constrained at        0.5 Check
PLAT300_ALERT_4_G Atom Site Occupancy of H68A       Constrained at        0.5 Check
PLAT300_ALERT_4_G Atom Site Occupancy of H69A       Constrained at        0.5 Check
PLAT300_ALERT_4_G Atom Site Occupancy of H70A       Constrained at        0.5 Check
PLAT301_ALERT_3_G Main Residue  Disorder ..............(Resd  1  )         3% Note
PLAT302_ALERT_4_G Anion/Solvent/Minor-Residue Disorder (Resd  2  )       100% Note
PLAT302_ALERT_4_G Anion/Solvent/Minor-Residue Disorder (Resd  3  )       100% Note
PLAT410_ALERT_2_G Short Intra H...H Contact  H47      ..H59      .       2.10 Ang.
                                                      x,y,z  =      1_555 Check
PLAT764_ALERT_4_G Overcomplete CIF Bond List Detected (Rep/Expd) .       1.17 Ratio
PLAT860_ALERT_3_G Number of Least-Squares Restraints .............        175 Note
PLAT933_ALERT_2_G Number of HKL-OMIT Records in Embedded .res File          4 Note
PLAT941_ALERT_3_G Average HKL Measurement Multiplicity ...........        4.1 Low
```

---

0 **ALERT level A** = Most likely a serious problem - resolve or explain
1 **ALERT level B** = A potentially serious problem, consider carefully
5 **ALERT level C** = Check. Ensure it is not caused by an omission or oversight
59 **ALERT level G** = General information/check it is not something unexpected

0 ALERT type 1 CIF construction/syntax error, inconsistent or missing data
10 ALERT type 2 Indicator that the structure model may be wrong or deficient
8 ALERT type 3 Indicator that the structure quality may be low
47 ALERT type 4 Improvement, methodology, query or suggestion
0 ALERT type 5 Informative message, check

---

It is advisable to attempt to resolve as many as possible of the alerts in all categories. Often the minor alerts point to easily fixed oversights, errors and omissions in your CIF or refinement strategy, so attention to these fine details can be worthwhile. In order to resolve some of the more serious problems it may be necessary to carry out additional measurements or structure refinements. However, the purpose of your study may justify the reported deviations and the more serious of these should normally be commented upon in the discussion or experimental section of a paper or in the "special_details" fields of the CIF. checkCIF was carefully designed to identify outliers and unusual parameters, but every test has its limitations and alerts that are not important in a particular case may appear. Conversely, the absence of alerts does not guarantee there are no aspects of the results needing attention. It is up to the individual to critically assess their own results and, if necessary, seek expert advice.

**Publication of your CIF in IUCr journals**

A basic structural check has been run on your CIF. These basic checks will be run on all CIFs submitted for publication in IUCr journals (*Acta Crystallographica*, *Journal of Applied Crystallography*, *Journal of Synchrotron Radiation*); however, if you intend to submit to *Acta Crystallographica Section C* or *E* or *IUCrData*, you should make sure that full publication checks are run on the final version of your CIF prior to submission.

**Publication of your CIF in other journals**

Please refer to the *Notes for Authors* of the relevant journal for any special instructions relating to CIF submission.

---

**PLATON version of 10/05/2023; check.def file version of 10/05/2023**

**Datablock e21msh10-cpd-4** - ellipsoid plot

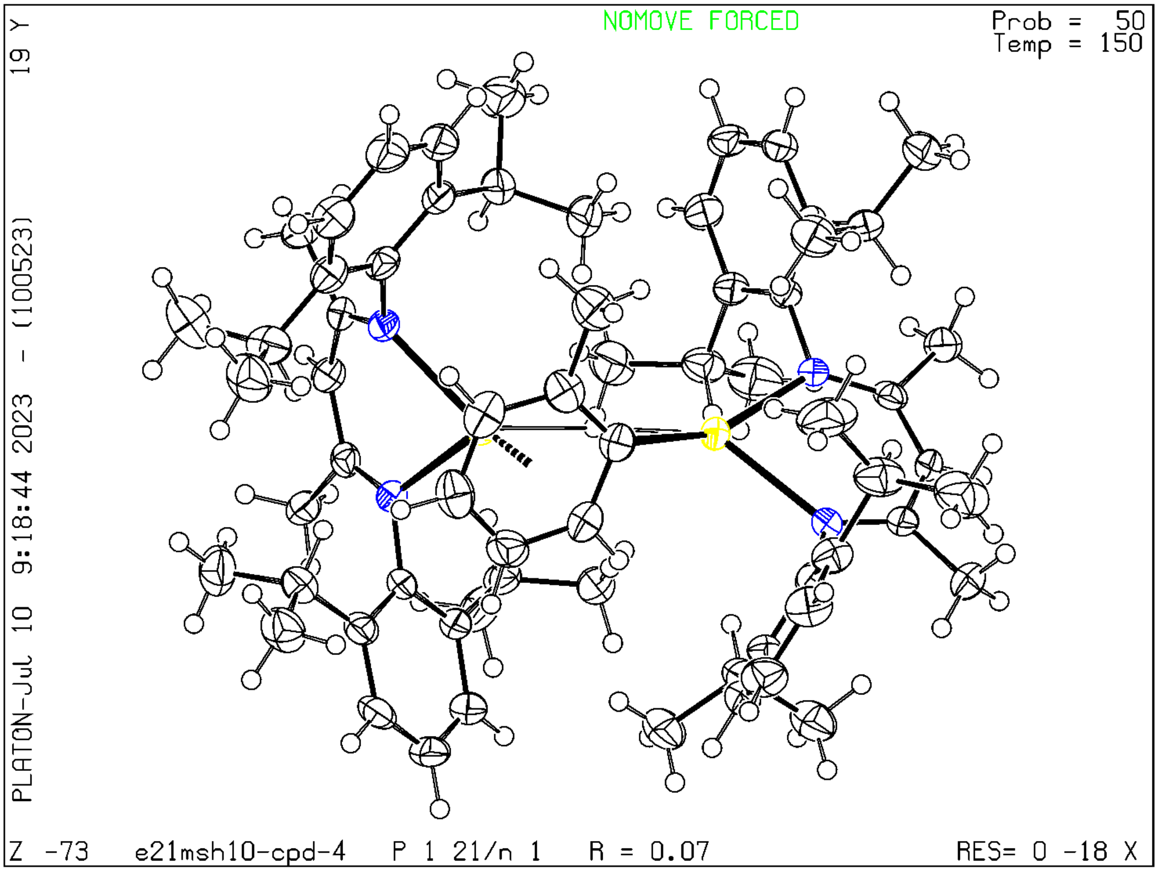

**Datablock e21msh08-cpd-5** - ellipsoid plot

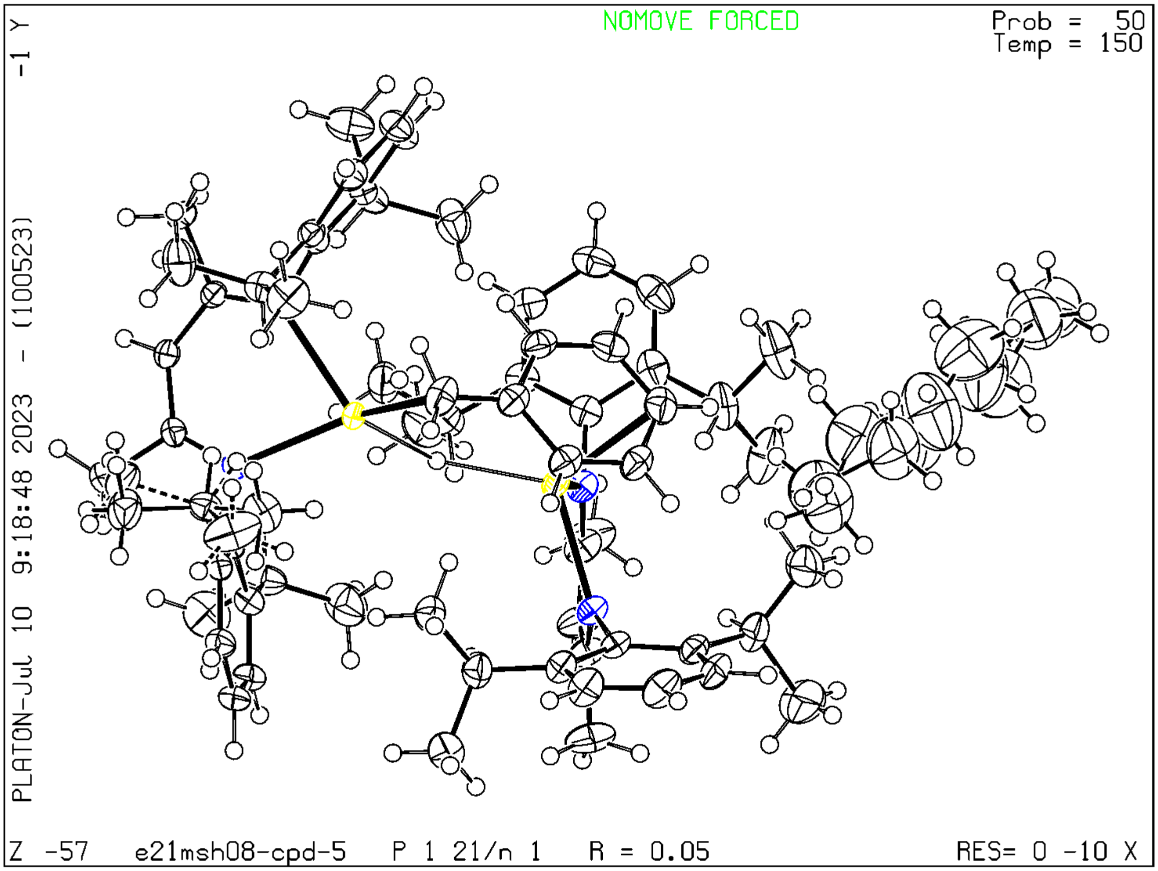

**Datablock e22msh05-cpd-7** - ellipsoid plot

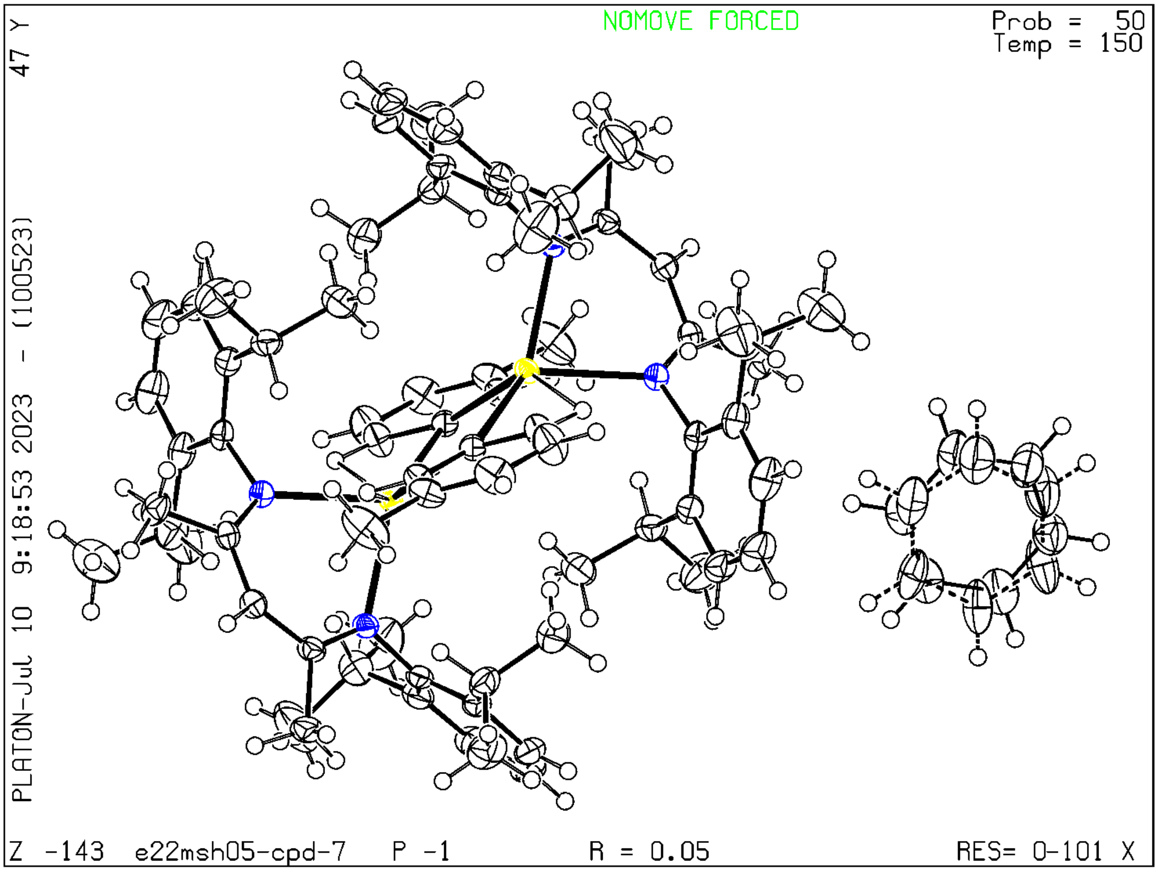

**Datablock s22msh16-cpd-8** - ellipsoid plot

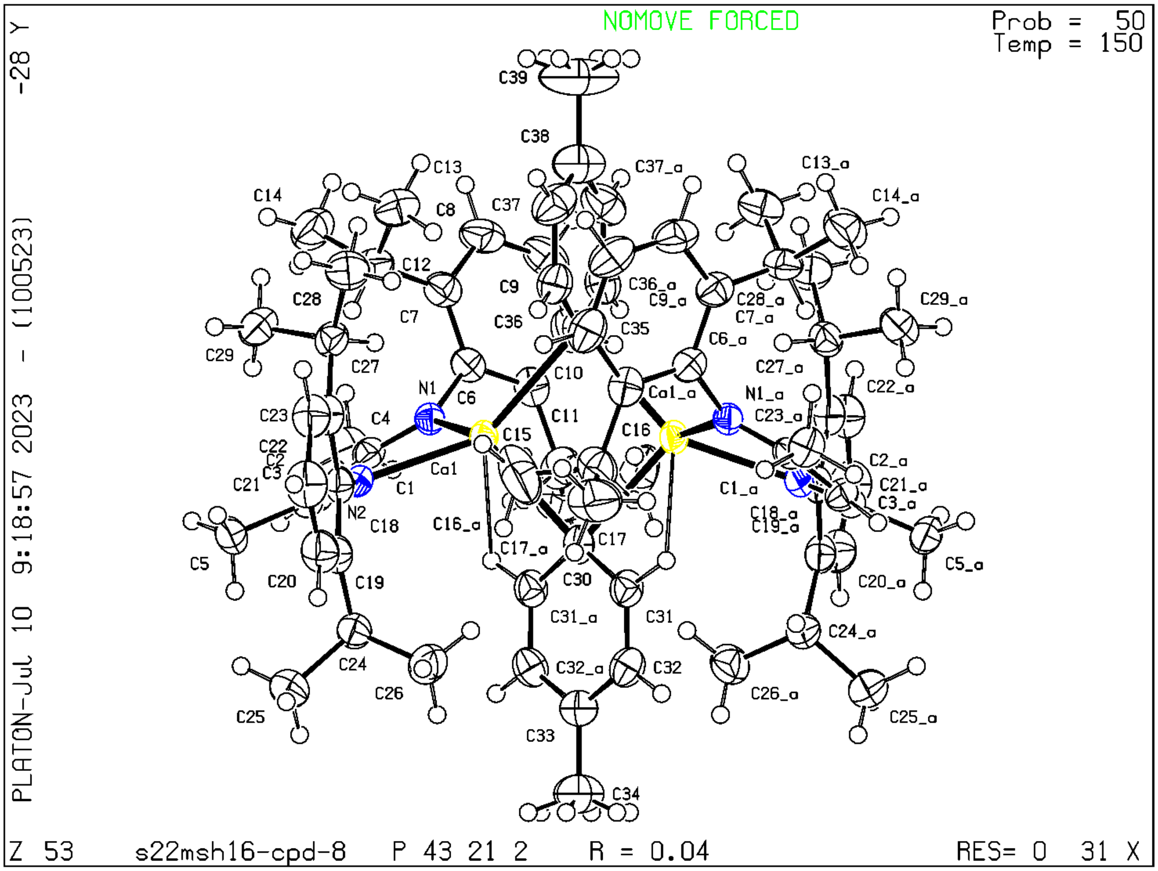

**Datablock s22msh81-cpd-9** - ellipsoid plot

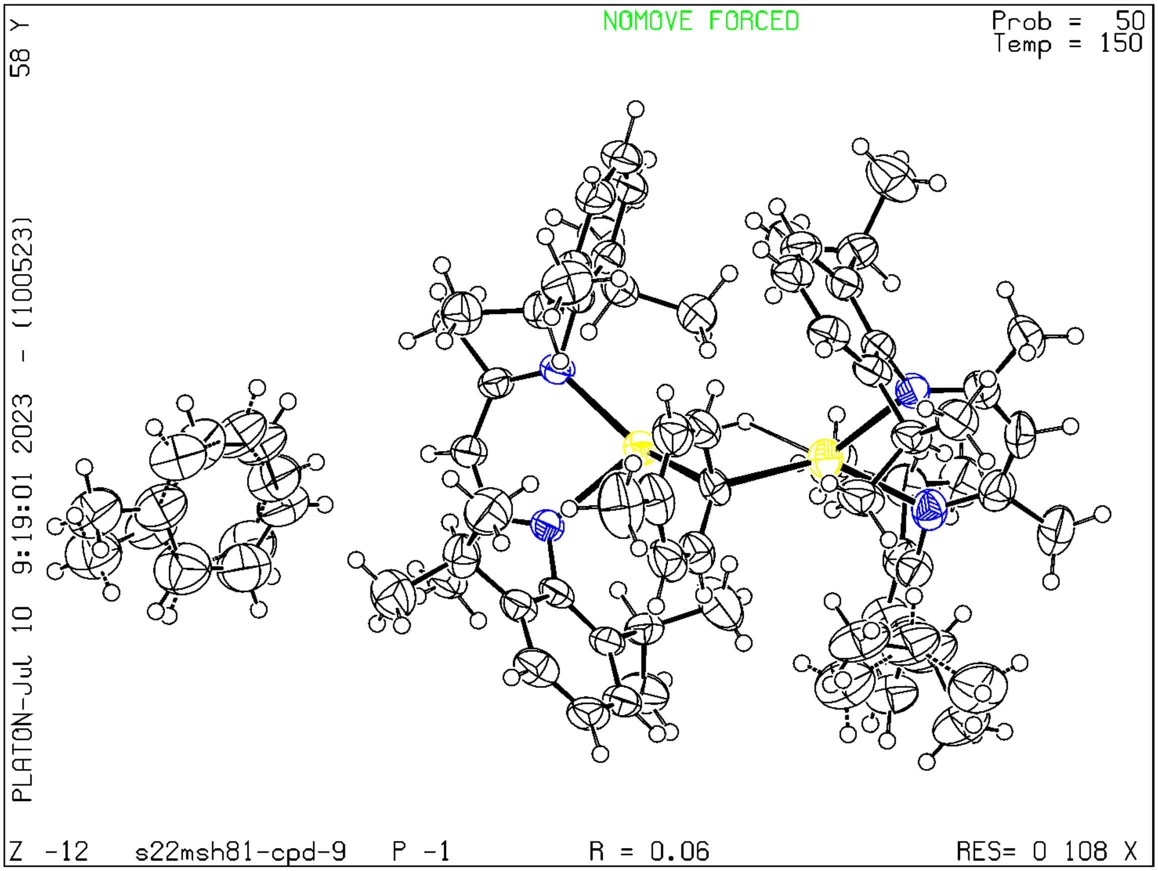

**Datablock e21msh25-cpd-10** - ellipsoid plot

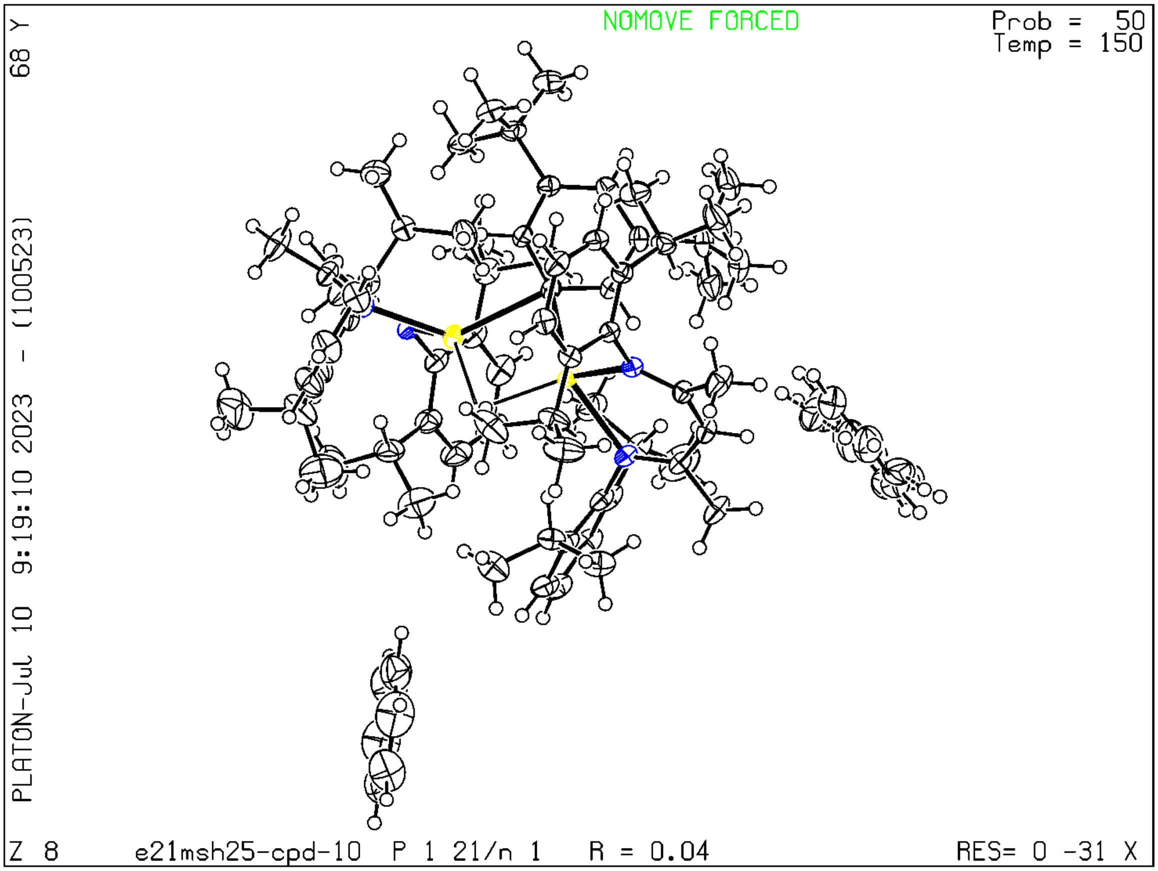

**Datablock s21msh59-cpd-11** - ellipsoid plot

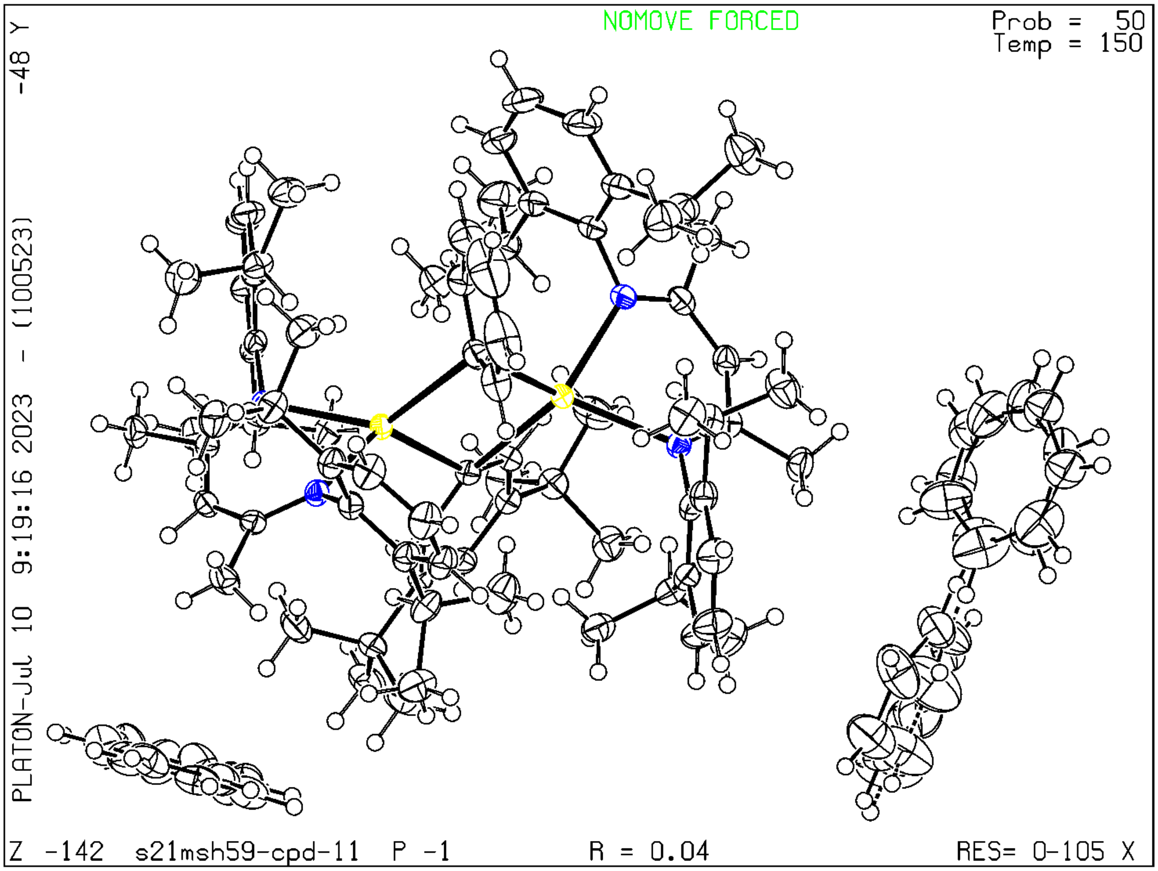

**Datablock s21msh68-cpd-12** - ellipsoid plot

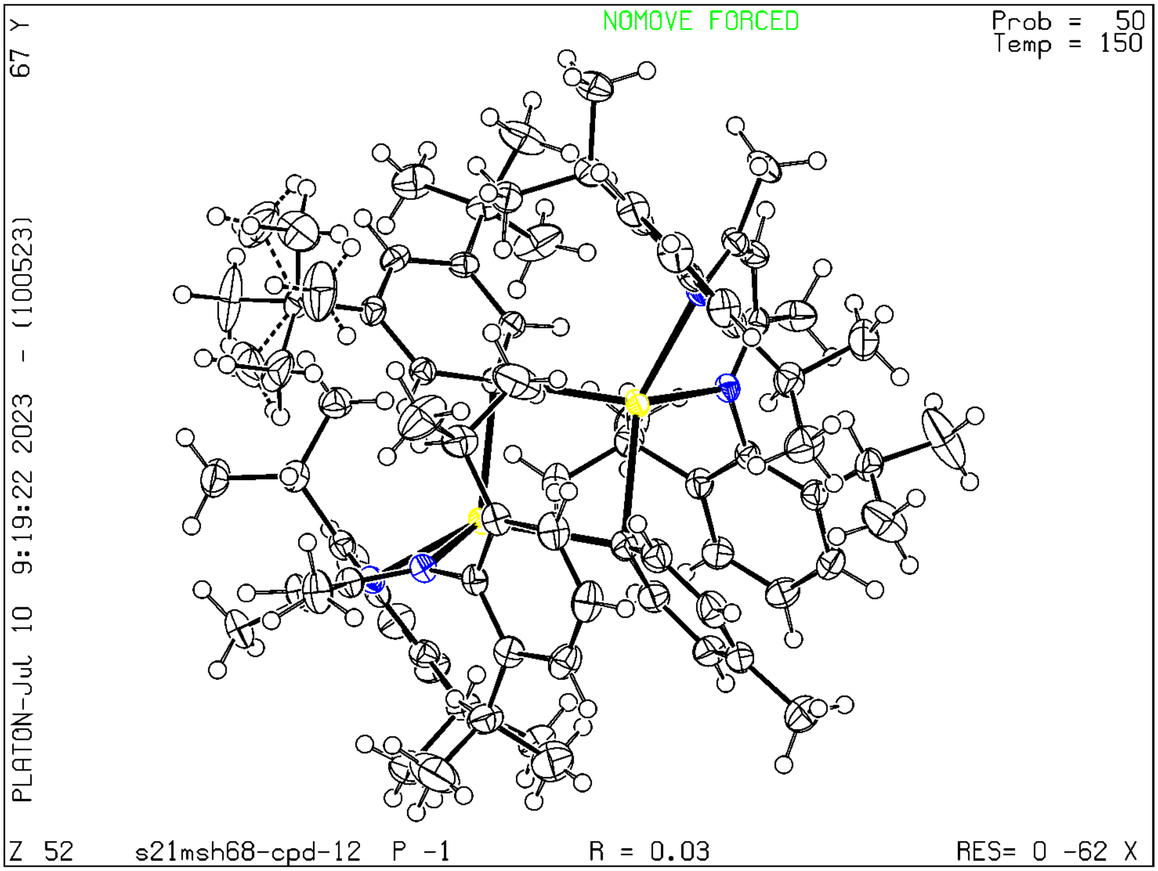

**Datablock s22msh86-cpd-14** - ellipsoid plot

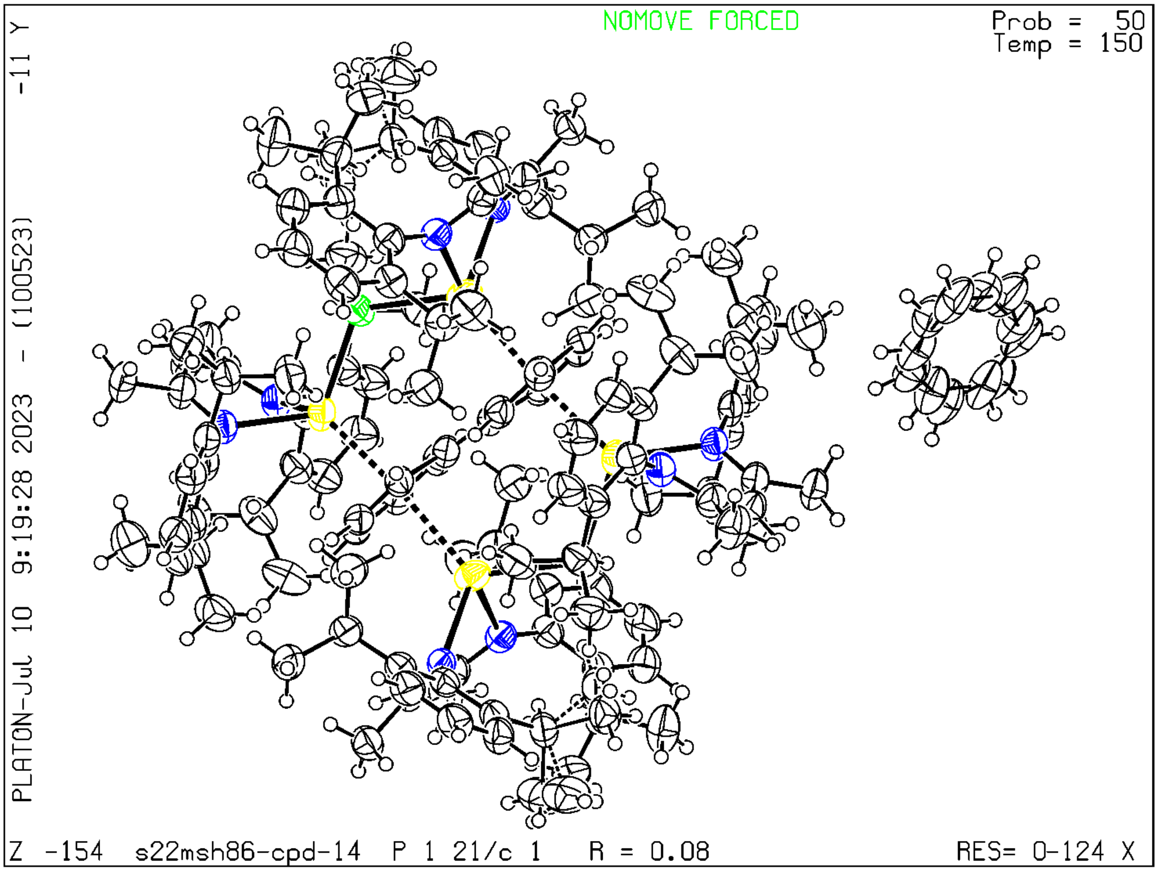

Supplement: Supplementary file 2 — Supporting Information [file checkcif-updated.pdf › checkcif-updated.pdf]
